# A Multicatalytic Cascade for the Stereoselective Synthesis of 1,4-Chiral Nitro Alcohols

**DOI:** 10.1021/acs.orglett.5c05184

**Published:** 2026-01-02

**Authors:** Christian Ascaso-Alegre, Álvaro Linacero-Gracia, Patricia Ferreira, Raquel P. Herrera, Juan Mangas-Sánchez

**Affiliations:** † Institute of Chemical Synthesis and Homogeneous Catalysis (ISQCH), 16765 Spanish National Research Council (CSIC)-University of Zaragoza, Pedro Cerbuna 12, 50009 Zaragoza, Spain; ‡ Department of Biochemistry and Molecular and Cellular Biology and Institute of Biocomputation and Physics of Complex Systems (BIFI, GBsC-CSIC Joint Unit), University of Zaragoza, Pedro Cerbuna 12, 50009 Zaragoza, Spain; § Department of Organic and Inorganic Chemistry, IUQOEM, 16763 University of Oviedo, Julián Clavería 8, 33006 Oviedo, Spain

## Abstract

Artificial cascades offer a powerful strategy to access high-value chemicals efficiently. Here, we report the integration of metal catalysis, organocatalysis, and biocatalysis in a one-pot stereodivergent process to synthesize chiral 1,4-nitro alcohols bearing two stereocenters. Optimization of each step enabled the efficient and selective formation of all four diastereomers with excellent stereoselectivity (up to 97:3 dr and er) and good yields. This work highlights the potential of multicatalytic cascades for the synthesis of stereochemically rich molecules.

## Graphical abstract

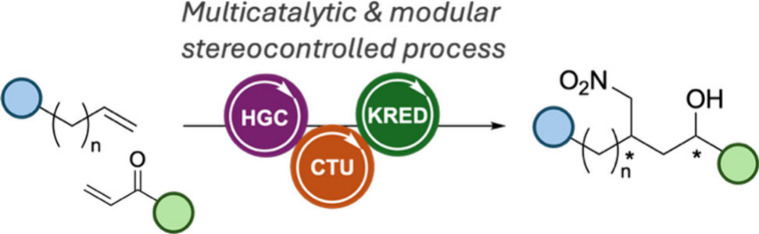

Artificial chemoenzymatic cascades, which mirror Nature’s multicatalytic pathways for metabolite synthesis, combine multiple consecutive reaction steps to access complex molecules.^1^ The design of synthetic routes to access high-value chemicals using these approaches provides many practical and environmental benefits. In contrast to the iterative cycles of reaction, isolation, and purification of each of the synthetic steps in classical synthetic chemistry, the combination of several reactions in a sequential or concurrent manner in the same reaction vessel allows for access to more molecularly complex chemicals, circumventing the need for intermediate isolation procedures that are time-consuming, generate additional waste, and require the use of large amounts of solvents and energy.^2–6^ Also, hazardous or unstable intermediate species can be readily converted in the reaction mixture, leading to higher yields and safer synthetic methods. Those advantages have drawn an increasing amount of interest to study the synthetic value of combining different catalytic strategies in cascade processes.^7–16^ For instance, in our continuous search for efficient strategies to obtain chiral compounds, we have recently described a new method to make chiral 1,4-nitro alcohols from simple and readily available aldehydes (Scheme 1).^17^ This one-pot sequential approach involved a Wittig olefination step of benzaldehyde derivatives, followed by an organocatalytic conjugate addition of MeNO_2_ and a ketoreductase-mediated bioreduction. Through this strategy, we could access a set of enantioenriched 1,4-nitro alcohols in excellent diastereomeric (dr) and enantiomeric (er) ratios. However, this approach presented several limitations. The starting Wittig olefination step suffers from a low atom economy and is not catalytic, presenting limitations from a sustainable chemistry perspective.^18^ Moreover, initial purification of the starting materials was necessary to maximize yields and avoid possible interactions between the carboxylic acid and the organocatalyst.^13,19^ With this, we envisaged an alternative route consisting of an initial Ru-mediated cross-metathesis (CM) step starting from styrenes and alkyl vinyl ketones to generate the corresponding enones and, in this manner, the possibility to synthesize chiral 1,4-nitro alcohol scaffolds through a fully multicatalytic three-step, one-pot approach.

**Scheme 1:**
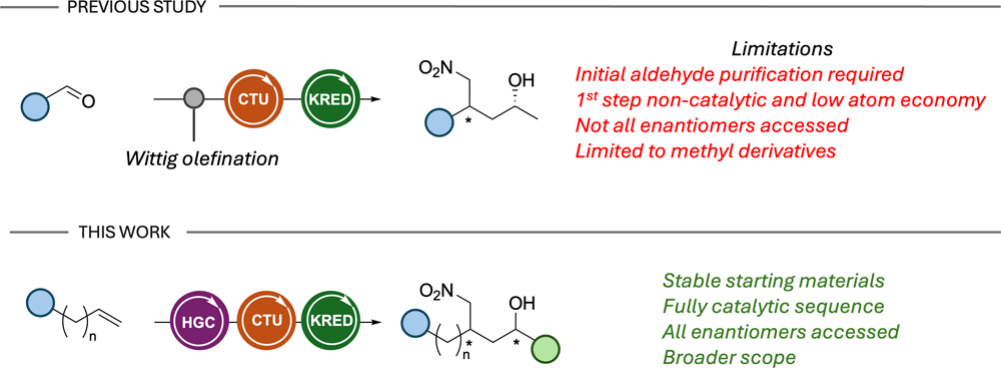
Previous and Current Contributions to the Synthesis of Chiral 1,4-Nitro Alcohols via Three-Step, One-Pot Sequential Cascades^a^

a Abbreviations: CTU, chiral thiourea; KRED, ketoreductase; HGC, Hoveyda–Grubbs catalyst.

To establish a basis for the envisaged process, we initially tested a series of Ru-based catalysts for the CM reaction between styrene (**1a**) and methyl vinyl ketone (MVK, **2a**) to access 4-phenyl-3-buten-2-one (**3aa**). The catalyst panel included commercially available first- and second-generation Grubbs catalysts (**I**–**VI**) and Hoveyda–Grubbs catalysts (**VII** and **VIII**) (Figure S1). Based on previous reports indicating that halogenated solvents and ethers enhance catalyst activation,^20^ and from our own experience, we selected methyl *tert*-butyl ether (MTBE) as the solvent for the initial screening (Table S1). Among the catalysts tested, second-generation Grubbs catalyst **IV** and Hoveyda–Grubbs catalyst **VIII** provided the highest analytical yields (87% and 82%, respectively) and were selected for further optimization. Next, and considering the optimized conditions for the conjugate addition and bioreduction processes,^17^ we evaluated the best-performing catalysts (**IV** and **VIII**) under identical conditions in cyclohexane (Table 1). Catalyst **VIII** outperformed **IV**, affording an isolated yield of 78% compared to 54%. Then, the effect of catalyst loading was studied (entries 2–5), noting a modest reduction in yield at 1.5 mol %. Interestingly, analysis of the reaction mixture revealed incomplete consumption of MVK while **1a** was no longer detectable. This suggests that styrene may be undergoing homodimerization, a plausible scenario supported by the known dimerization tendencies of these alkenes.^21^ Given that MVK also functions as a Michael acceptor in the subsequent organocatalytic step, we aimed to ensure its full conversion in the CM step. Increasing the number of equivalents of **1a** led to no improvement in product yield but did enhance the formation of the homodimer byproduct (entries 6 and 7). To address this, we modified the addition strategy for **1a**, achieving better results when it was introduced stepwise (entries 8 and 9). While we hypothesized that increasing the reaction temperature might favor the CM pathway, it instead resulted in lower yields. After the optimization process, the optimal reaction conditions were determined to be 2 mol % catalyst **VIII**, 3 equiv of **1a** added stepwise, and 1 equiv of **2a** in cyclohexane at 50 °C for 24 h.

**Table 1:**
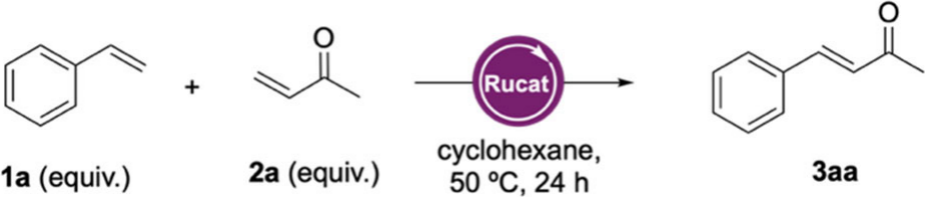
Optimization of the Catalytic Cross-Metathesis Step between Styrene (**1a**) and MVK (**2a**)­^a^

entry	catalyst	loading (mol %)	**1a** (equiv)	**2a** (equiv)	isolated yield (%)^b^
1	**IV**	3	1	1	54
2	**VIII**	3	1	1	78
3	**VIII**	2.5	1	1	78
4	**VIII**	2	1	1	79
5	**VIII**	1.5	1	1	74
6	**VIII**	2	1.2	1	76
7	**VIII**	2	1.5	1	77
8^c^	**VIII**	2	2	1	83
9^d^	**VIII**	2	3	1	86
10^e^	**VIII**	2	1	3	32
11^d^ ^,^ ^f^	**VIII**	2	3	1	72

a Conditions: cyclohexane at 50 °C for 24 h.

b Yields are for the isolated product after column chromatography.

c Stepwise addition of **1a** (1 equiv at time zero and 1 equiv after 4 h).

d Stepwise addition of **1a** (1.5 equiv at time zero and 1.5 equiv after 4 h).

e Stepwise addition of **2a** (1.5 equiv at time zero and 1.5 equiv after 4 h).

f Reaction temperature of 60 °C.

We next examined the combination of the CM process with the asymmetric conjugate addition of nitromethane over **3aa**. For that, we used the chiral cyclohexanediamine thiourea (*R*,*R*)-**IX** as the catalyst to access chiral nitro ketone **4aa**, initially under our previously optimized conditions,^17^ i.e., 15 mol % (*R*,*R*)-**IX** and 10 equiv of MeNO_2_ at room temperature for 120 h. To our delight, we obtained (*R*)-**4aa** in 64% isolated yield and 95:5 er, although we observed a decrease in stereoselectivity compared to our original process (99:1 er). This result hinted at a possible interaction between **VIII** and (*R*,*R*)-**IX**, so we decided to test different strategies to remove or inactivate **VIII** after the first step. Filtration through a pad of Celite resulted in no change in the er of the product. Next, we examined the addition of different ligands to inactivate **VIII** such as imidazole, 2-mercaptonicotinic acid, and cysteine (Table S2).^22^ Reactions were run for 1 h after the addition of catalytic amounts (6 mol %) of these species prior to the organocatalytic step. In all cases, an increase in the er of (*R*)-**4aa** was found, with imidazole being the additive that afforded the best results (64% yield, 97:3 er). Finally, MeNO_2_ and (*R*,*R*)-**IX** loadings were also adjusted, which had a positive effect when using 20 mol % (*R*,*R*)-**IX**, affording (*R*)-**4aa** in 84% isolated yield and 97:3 er. Once the integration of both steps was optimized, we explored the reaction scope starting from different styrene derivatives bearing electron-withdrawing (EWG) and electron-donating (EDG) groups (Scheme 2). For derivatives bearing a strong EWG on the aromatic ring, such as (*R*)-**4da**, the organocatalytic step proceeds almost quantitatively, while the metathesis step limits the overall yield (71% isolated yield). This is attributed to the favored homodimerization of styrene derivative **1d** in these substrates, resulting in significant formation of the homodimerization byproduct and the MeNO_2_–MVK addition product due to unreacted MVK remaining from the first step. In contrast, for derivatives (*R*)-**4ba**, (*R*)-**4ca**, and (*R*)-**4ea**, the organocatalytic step affords lower conversion; however, the CM step proceeded more efficiently, with minimal detectable residual MVK. Notably, in the case of (*R*)-**4ba**, the product was obtained in a remarkably high yield (89% isolated yield). To broaden the scope, we also evaluated a selection of allylbenzene derivatives (**1f**–**h**). Given their distinct reactivity profiles, CM and conjugate addition steps were individually examined using isolated yield and NMR conversion, respectively (Scheme S1). Allylbenzene **1f** and its 3,4-dimethoxy analogue **1g** gave improved CM yields (71% in both cases) compared to **1a**, though subsequent organocatalytic conversion was lower (36% and 38% conversion, respectively), resulting in a modest overall isolated yield of (*S*)-**4ga** (24%) while (*S*)-**4fa** could not be isolated in pure form (Scheme 2). Interestingly, for **1h**, the CM step was less efficient (57% yield), yet the conjugate addition proceeded with a higher conversion (53%) (Scheme S1), likely due to increased electrophilicity caused by the electron-withdrawing fluorine, affording (*S*)-**4ha** in 26% overall yield. In all cases, compounds (*R*)-**4aa–ea**, (*S*)-**4ga**, and (*S*)-**4ha** were obtained with excellent enantiomeric ratios (up to >99:1), highlighting the compatibility of both catalytic systems in the synthesis of chiral 1,4-nitroketones **4**.

**Scheme 2:**
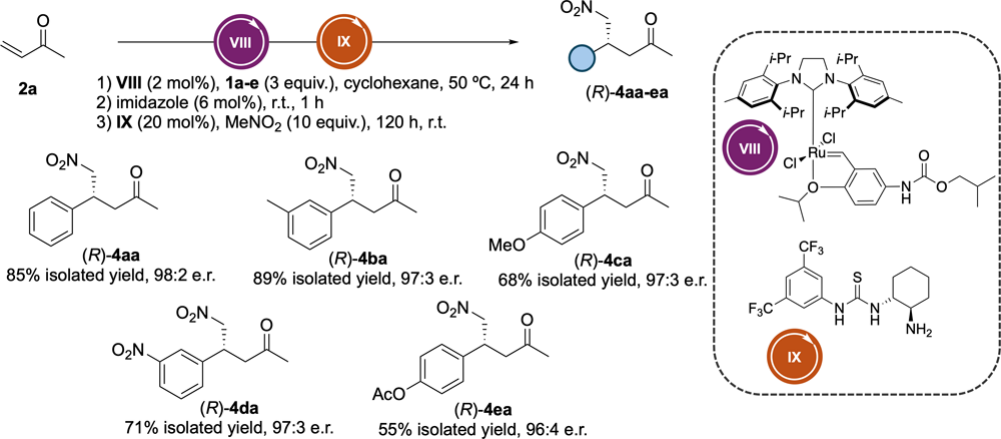
Chiral 1,4-Nitro Ketone Synthesis via a One-Pot Sequential Approach Starting from Different Styrenes **1a**–**h** and MVK (**2a**)­^a^

a The process involves a Ru-catalysed cross-metathesis step followed by an asymmetric conjugate addition of MeNO_2_ catalyzed by chiral thiourea (*R*,*R*)-**IX**. Yields are for the isolated product after column chromatography. Enantiomeric ratios were determined by HPLC on a chiral stationary phase.

Following our previous work, we explored the integration of a bioreduction step to stereoselectively access all four possible diastereomers of chiral 1,4-nitro alcohols **5** (Scheme 3). For the synthesis of (*R*)-configured products, we had previously identified the commercially available ketoreductase Evo200 as a robust and suitable catalyst. Gratifyingly, starting from substrates **1a** and **2a** and combining Hoveyda–Grubbs catalyst **VIII** with organocatalyst (*R*,*R*)-**IX** under our optimized bioreduction conditions (1 mg mL^–1^ Evo200, 100 mM NaP_i_ buffer (pH 6), stepwise addition over 72 h at 40 °C, 5% (v/v) *i*PrOH, 10% (v/v) DMSO, 0.1 mM NAD^+^), we successfully obtained (2*R*,4*R*)-**5aa** in 76% isolated yield, with excellent stereoselectivity (97:3 dr and er (Scheme 3)). To the best of our knowledge, this represents the first sequential one-pot cascade integrating metal catalysis, organocatalysis, and biocatalysis to furnish a chiral compound bearing two stereocenters in a fully stereocontrolled manner. For the synthesis of (*S*)-configured products, we turned to alcohol dehydrogenase ADH-A from *Rhodococcus ruber*, for its broad substrate scope, NAD^+^ preference, and tolerance to organic solvents.^23^ ADH-A was overexpressed in *Escherichia coli* and used as a cell-free extract (CFE, 10 mg mL^–1^) in 100 mM Tris-HCl buffer (pH 7) after the CM–Michael addition sequence involving **VIII** and (*R*,*R*)-**IX**. This approach afforded (2*S*,4*R*)-**5aa** in 45% yield, again with high stereoselectivity (97:3 dr and er). With the full cascade optimized, we then accessed the two remaining diastereomers by employing (*S*,*S*)-**IX** in the conjugate addition step. Using Evo200, we isolated (2*R*,4*S*)-**5aa** in 54% yield; with ADH-A, we obtained (2*S*,4*S*)-**5aa** in 62% yield, both with 97:3 dr and er. To broaden the scope, we also evaluated our process starting from ethyl-vinyl ketones **2b** and **1a**. For the resulting ethyl-substituted nitro alcohols (**5ab**), only two stereoisomers, (3*R*,5*R*)-**5ab** and (3*R*,5*S*)-**5ab**, could be obtained, as ADH-A failed to accept the corresponding nitro ketones as substrates, likely due to steric factors as previously suggested (Scheme 3).^24,25^ These were isolated in moderate yields (42–50%) due to the reduced ability of Evo200 to accept bulkier substrates, although with excellent stereoselectivity (≥97:3 dr and er). Finally, with regard to allylbenzene derivatives, we selected **1h** as the model substrate. In this case, as the CM was the limiting step, the initial optimization was performed to minimize homodimerization. By adopting a stepwise addition strategy, adding 2 equiv of **1h** in two portions, we improved the yield of enone **3ha** to 90%. This enabled full cascade integration of substrate **1h**, affording all four diastereomers of **5ha** in modest overall yields (22–28%), although in high diastereomeric and enantiomeric ratios (up to 96:4). Finally, we performed a 1 mmol scale-up to access chiral 1,4-nitroalcohol (2*R*,4*R*)-**5aa** from **1a** and **2a**, using **VIII**, (*R*,*R*)-**IX**, and Evo200 as the catalysts. Following the optimal procedure, (2*R*,4*R*)-**5aa** was obtained in 43% isolated yield.

**Scheme 3:**
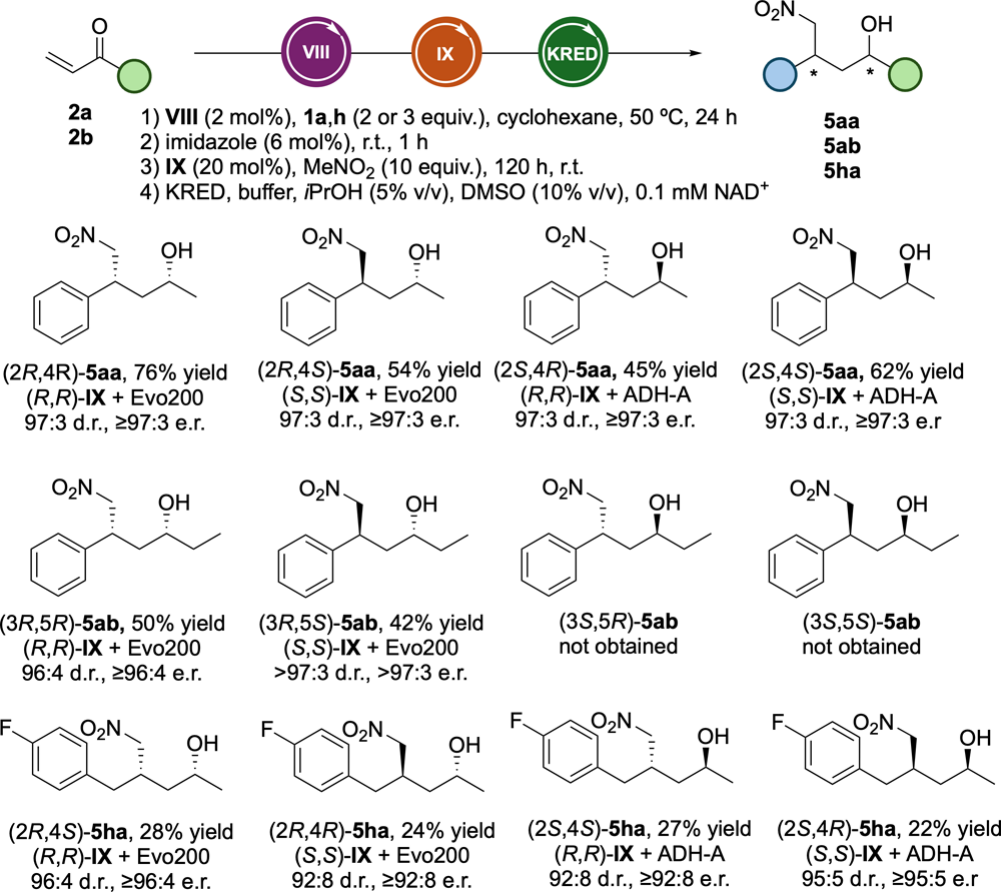
Artificial Multienzymatic Cascade for One-Pot Sequential Access to Chiral 1,4-Nitroalcohols **5** from Alkenes **1a** and **1h** and Vinyl Ketones **2a** and **2b** ^a^

a Yields are for the isolated product after column chromatography. Conversions and diastereomeric and enantiomeric ratios were determined by either ^1^H NMR spectroscopy or HPLC on a chiral stationary phase.

In summary, we have developed a robust and stereocontrolled sequential one-pot cascade integrating three distinct catalytic approaches, Ru-catalyzed cross-metathesis (CM), organocatalytic conjugate addition, and biocatalytic reduction, to access all four diastereomers of chiral 1,4-nitro alcohols. Hoveyda–Grubbs catalyst **VIII** was identified as being optimal for the CM step, and its performance was significantly improved through a stepwise addition strategy to minimize side reactions. This modification resulted in a substantial increase in the atom efficiency of the first step compared to our previous approach (84% vs 35%).^26^ The asymmetric conjugate addition of nitromethane catalyzed by chiral thiourea **IX** proceeded efficiently under modified conditions, and strategies to deactivate residual Ru species (e.g., using imidazole) successfully restored high enantioselectivity. The final bioreduction step employed either Evo200 or ADH-A enzymes to deliver the desired products with excellent diastereo- and enantioselectivity (up to 97:3 dr and er). This modular catalytic platform enabled the stereodivergent synthesis of all four diastereomers of **5aa** and demonstrated good functional group tolerance across a range of substituted styrenes and vinyl ketones. Although the cascade efficiency was dependent on the substrate with the metathesis or organocatalytic step acting as the limiting stage in some cases, the methodology could be adapted through tailored optimization strategies. To the best of our knowledge, this work represents the first example of a fully stereocontrolled one-pot sequence combining metal catalysis, organocatalysis, and biocatalysis to make enantioenriched molecules from prochiral compounds. This approach provides a valuable example of the streamlined synthesis of complex stereochemically defined molecules using orthogonal catalytic methods.

## Supplementary Material



## Data Availability

The data underlying this study are available in the published article and its Supporting Information.

## Notes

Materials, experimental procedures, analytical methods, GC traces and spectra, and ^1^H and ^13^C­{^1^H}-APT NMR spectra (PDF)
