# Supplementary material for: A Multicatalytic Cascade for the Stereoselective Synthesis of 1,4-Chiral Nitro Alcohols
Source: Org Lett. 2026 Jan 2;28(2):879–83. doi: 10.1021/acs.orglett.5c05184 (PMC12814524; doi:10.1021/acs.orglett.5c05184)
Supplement: Supplementary file 1 [file ol5c05184_si_001.pdf]

SUPPORTING INFORMATION FOR

A Multicatalytic Cascade for the Stereoselective Synthesis of 1,4-Chiral Nitro Alcohols

Christian Ascaso-Alegre, Álvaro Linacero-Gracia, Patricia Ferreira, Raquel P. Herrera and Juan Mangas-Sánchez*

C. Ascaso-Alegre, A. Linacero-Gracia, R. P. Herrera

Institute of Chemical Synthesis and Homogeneous Catalysis (ISQCH, Spanish National Research Council (CSIC)-University of Zaragoza, Pedro Cerbuna 12, 50009 Zaragoza, Spain.

P. Ferreira

Department of Biochemistry and Molecular and Cellular Biology and Institute of Biocomputation and Physics of Complex Systems (BIFI, GBsC-CSIC Joint Unit), University of Zaragoza, Pedro Cerbuna 12, 50009 Zaragoza, Spain

J. Mangas-Sánchez

Department of Organic and Inorganic Chemistry, IUQOEM, University of Oviedo, Julián Clavería 8, 33006 Oviedo, Spain.

E-mail: mangasjuan@uniovi.es

Table of Contents

1. General information 3

2. Commercially available Ru-based catalysts used in this contribution 4

3. General procedure for the preparation of ADH-A from *Rhodococcus ruber* lysate 4

4. General procedure for the Ru-catalysed cross-metathesis reaction between 1a and 2a. 5

5. Study of interaction between Ru catalyst VIII and organocatalyst IX 6

6. Investigation of the one-pot cascade to access chiral 1,4-nitro ketones (*S*)-4fa-ha from allylbenzene derivatives 1f-h 7

7. General procedure for the synthesis of chiral nitroketones (*R*)-4aa-ea 8

9. Procedure for the scale-up synthesis of chiral 1,4-nitroalcohol (2*R*,4*R*)-5aa 12

10. General procedure for the synthesis of chiral 1,4-nitroalcohols 5aa, 5ab y 5ha using ADH-A 12

11. General procedure for the synthesis of racemic 1,4-nitroalcohols 5ab and 5ha 14

12. Chiral HPLC analysis of 1,4-nitroketones 4aa-4ea 14

13. Spectroscopic data of studied compounds 21

14. Determination of enantiomeric and diastereomeric ratios by NMR 39

15. Calibration curves for the determination of conversion in metathesis reactions by GC 45

1. General information

The starting materials **1a–h** and **2a,b**, as well as the metal catalysts used in this study (**I-VIII**), are commercially available and were purchased from Merck Sigma Aldrich. Organocatalysts (*S*,*S*)- and (*R*,*R*)-**IX** were prepared as previously described.[1]

Reactions were monitored by thin-layer chromatography (TLC) using aluminum plates coated with silica gel and a fluorescent indicator (60 F254, 0.2 mm). Compounds were visualized at 254 nm using UV light. Products **5aa**, **5ab**, and **5ha** were isolated by column chromatography using silica gel (0.06–0.2 nm) as the stationary phase and commercial mixtures of *n*-hexane/EtOAc as the eluent.

Chiral HPLC analysis of products **4aa**–**4ea** was performed on a Waters 600 system using a Daicel ChiralPak IC or Phenomenex i-Amylose-1 column as the stationary phase and commercial mixtures of *n*-hexane or *n*-heptane/isopropyl alcohol or ethyl acetate as the eluent. The absolute configuration of products **5aa**, **5ab**, and **5ha** was assigned by comparison with previously reported data[1] and the stereopreference of enzymes provided by the manufacturer.

^{1}H NMR and ^{13}C{^{1}H}-APT NMR spectra of reagents and products were recorded at 300 MHz (Bruker ARX300 spectrometer), 400 MHz (Bruker AV400 spectrometer), or 500 MHz (Bruker AV500 spectrometer), using deuterated chloroform ($CDCl_3$) as the solvent. HRMS analysis of the products was performed using a MicroTof-Q mass spectrometer and electrospray ionization (ESI).

Evo200 KRED was acquired at evoxx Technologies GmbH. The enzyme was selected based on the manufacturer's information (https://evoxx.com/products/biocatalysis/alcohol-dehydrogenase/) attending to substrate scope, stereoselectivity and ability to work at high substrate loadings. Evo200 KRED comes as lyophilised powder with 89.5% protein content according to the manufacturer's information. Evo200 KRED corresponds to Alcohol dehydrogenase 200 (catalogue number evo-1.1.200s, Lot number 11200-M184516.01). According to the manufacturer's information, enzyme activity is 7.0 U mg^{-1} with U defined as µmol ethyl acetoacetate reduced per minute at pH 7.0 and 30 ºC.

The spectral data for products **3aa**,[2] **3fa**,[3] **3ga**,[4] **3ha**,[5] **4aa**[1], **4fa**,[2] **5aa**[1] are consistent with values previously reported in the literature.

---

[1] C. Ascaso-Alegre, R. P. Herrera, J. Mangas-Sánchez, *Angew. Chem. Int. Ed.* **2022**, *61*, e202209159.
[2] K. Mei, M. Jin, S. Zhang, P. Li, W. Liu, X. Chen, F. Xue, W. Duan, W. Wang, *Org. Lett.* **2009**, *11*, 2864–2867.
[3] N. S. Y. Loy, A. Singh, X. Xu, C.-M. Park, *Angew. Chem. Int. Ed.* **2013**, *52*, 2212−2216.
[4] T. Tanaka, K. Takahashi, Y. Inoue, N. Endo, E. Shimoda, K. Ueno, T. Ichiyanagi, T. Ohta, A. Ishihara, *Biosci. Biotechnol. Biochem.* **2024**, *88*, 389–398.
[5] C. Wang, X. Wu, H. Li, J. Qu, Y. Chen, *Angew. Chem. Int. Ed.* **2022**, *61*, e202210484.

2. Commercially available Ru-based catalysts used in this contribution

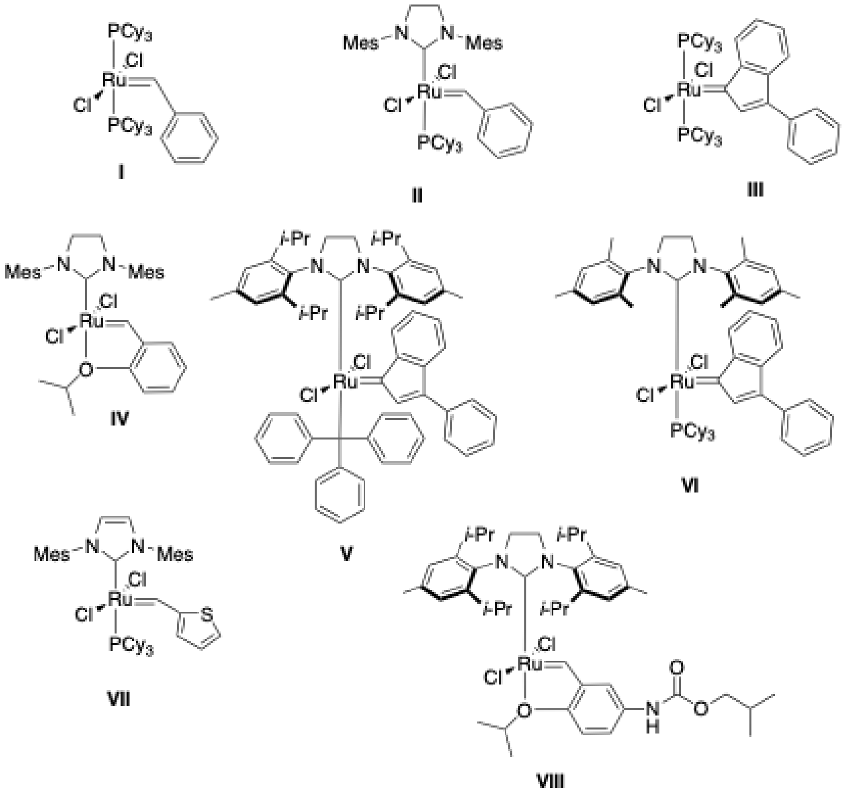

Figure S1. Commercially available first- and second-generation Grubbs catalysts (**I–VI**) and Hoveyda–Grubbs catalysts (**VII** and **VIII**) screened in this contribution.

3. General procedure for the preparation of ADH-A from *Rhodococcus ruber* lysate

A pET24b plasmid containing the gene coding for the ADH-A from *Rhodococcus ruber* (ADH-A) was kindly provided by Prof. Nicholas J. Turner. Plasmid was transformed into *Escherichia coli* (*E. coli*) BLC43 strain, and the gene expressed following the procedure described in the literature.[6] Bacterial pellets from 1 L of culture were resuspended in 10 mL of sample buffer supplemented with a protease inhibitor cocktail (complete EDTA-free Protease Inhibitor Tablets from Roche), sonicated, and then centrifuged at 4 °C for 20 minutes at 8000 × g. Subsequently, 20 mL of cell-free extract was transferred to a 50 mL falcon tube. The falcon tube was snap frozen in liquid nitrogen prior to freeze-drying for 1-2 days (until powdery). The freeze-dried cell-free lysate (CFE) was stored at -20 °C prior to use.

---

[6] K. Edegger, C. C. Gruber, T. M. Poessl, S. R. Wallner, I. Lavandera, K. Faber, F. Niehaus, J. Eck, R. Oehrlein, A. Hafner, W. Kroutil, *Chem. Commun.* **2006**, 2402–2404.

4. General procedure for the Ru-catalysed cross-metathesis reaction between 1a and 2a.

To a mixture of catalyst (**I-VIII**) in MTBE or cyclohexane (1 mL) placed in an test tube, MVK **2a** (0.25 mmol, 1.0 equiv) and styrene **1a** (0.25 mmol, 1.0 equiv) are added. The mixture is allowed to react at 50 °C for 24 hours using an oil bath. After this time time, the conversion to enone **3aa** was either determined by GC or product **3aa** was isolated by column chromatography.

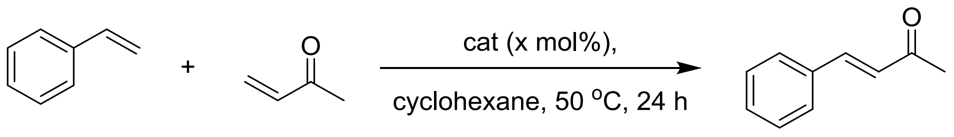

Table S1. Screening of catalysts **I-VIII** for the cross-metathesis reaction between **1a** and **2a**

Entry	Catalyst	Loading (mol%)	Solvent	GC or isolated yield (%)
1	**I**	3	MTBE	n.r.
2	**II**	3	MTBE	22
3	**III**	3	MTBE	n.r.
4	**IV**	3	MTBE	87[b]
5	**V**	3	MTBE	19
6	**VI**	3	MTBE	32
7	**VII**	3	MTBE	8
8	**VIII**	3	MTBE	82[b]
9	**IV**	3	cyclohexane	54[b]
10	**VIII**	3	cyclohexane	78[b]
11	**VIII**	2.5	cyclohexane	78[b]
12	**VIII**	2	cyclohexane	79[b]
13	**VIII**	1.5	cyclohexane	74[b]

General conditions: MVK **2a** (0.25 mmol, 1 equiv.) and **1a** (0.25 mmol, 1 equiv.) were added to a mixture containing the corresponding catalyst **I-VIII** (3-1.5 mol%) in MTBE or cyclohexane (1 mL) and was stirred at 50 ºC in an oil bath for 24 h. GC yields calculated via gas chromatography using calibration curves (Section S14). [b] Yields are for the isolated product after column chromatography.

5. Study of interaction between Ru catalyst VIII and organocatalyst IX

To a mixture of catalyst **VIII** (0.005 mmol, 0.02 equiv.) in cyclohexane (1 mL) placed in a test tube, MVK **2a** (0.25 mmol, 1.00 equiv) and styrene **1a** (0.375 mmol, 1.5 equiv) were added. The mixture was allowed to react at 50 °C using an oil bath for 4 hours. After this time, a second portion of styrene (0.375 mmol, 1.5 eq.) was added, and the reaction was continued for 24 hours. The reaction mixture was then brought to room temperature, and once this temperature was reached, additive (0.03 mmol, 0.06 mol%) was added. Stirring was continued for 1 hour. Subsequently, organocatalyst (*R*,*R*)-**IX** (0.1 mmol, 0.2 eq.) and $MeNO_2$ (5 mmol, 10 eq.) were added, and the reaction mixture stirred for 120 h at room temperature. Afterwards, the mixture was purified by column chromatography using a mixture of *n*-hexane and EtOAc in an 8:2 ratio as eluent, affording the corresponding product (*R*)-**4aa**.

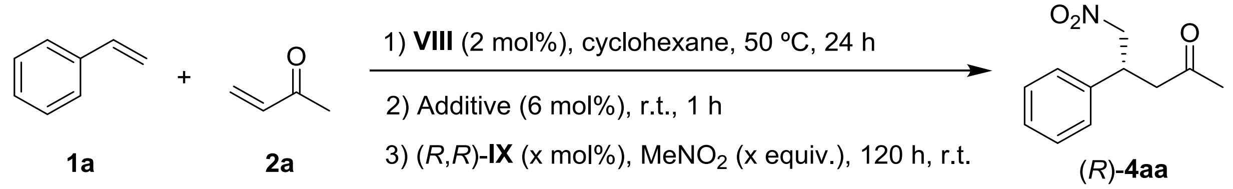

Table S2. Screening of different additives for the inactivation of catalyst **VII**

Entry	(*R*,*R*)-**IX** (mol%)	$MeNO_2$ (equiv.)	Additive	Yield (%)[b]	e.r. (*R*:*S*)[c]
1	15	10	-	64	95:5
2	15	10	imidazole	61	97:3
3	15	10	2-mercaptonicotinic acid	44	98:2
4	15	10	cysteine	55	97:3
5	15	20	imidazole	62	97:3
6	20	10	imidazole	84	97:3

General conditions: MVK **2a** (0.25 mmol, 1 equiv.) and **1a** (0.375 mmol, 1.5 equiv.) were added to a mixture containing catalyst **VIII** (0.005 mmol, 2 mol%) in cyclohexane (1 mL) and was stirred at 50 °C using an oil bath for 4 h. After that time, a second portion of **1a** (0.375 mmol, 1.5 equiv.) was added and the mixture was left to stir for 24 h. Then, the correponding additive (0.015 mmol, 6 mol%) was added at room temperature and was stirred for 1 h. Finally, organocatalyst (*R*,*R*)-**IX** and $MeNO_2$ were added and the mixture stirred for 120 h at r.t. [b] Yields are for the isolated product after column chromatography. [c] Enantiomeric ratios (e. r.) were determined by HPLC on a chiral stationary phase.

6. Investigation of the one-pot cascade to access chiral 1,4-nitro ketones (*S*)-4fa-ha from allylbenzene derivatives 1f-h

To a mixture of catalyst **VIII** (0.005 mmol, 0.02 equiv.) in cyclohexane (1 mL) placed in a test tube, MVK **2a** (0.25 mmol, 1.00 equiv) the corresponding allylbenzene derivative **1fa-ha** (0.375 mmol, 1.5 equiv) were added. The mixture was allowed to react at 50 °C in an oil bath for 4 hours. After this time, a second portion of styrene (0.375 mmol, 1.5 eq.) was added, and the reaction was continued for 24 hours. The reaction mixture was then brought to room temperature, and once this temperature was reached, imidazole (0.03 mmol, 0.06 mol%) was added. Stirring was continued for 1 hour. Subsequently, organocatalyst (*R*,*R*)-**IX** (0.1 mmol, 0.2 eq.) and $MeNO_2$ (5 mmol, 10 eq.) were added, and the reaction mixture stirred for 120 h at room temperature. Afterwards, the mixture was purified by column chromatography using a mixture of *n*-hexane and EtOAc in an 8:2 ratio as eluent, affording the corresponding products (*S*)-**4fa-ha**.

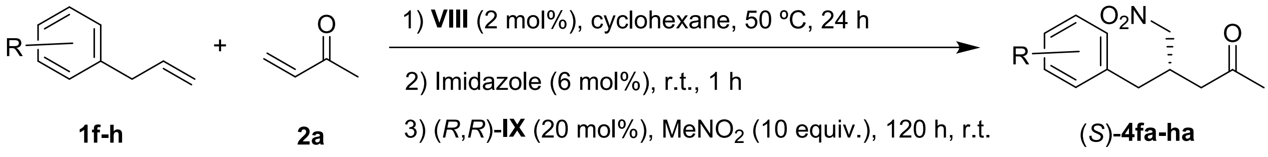

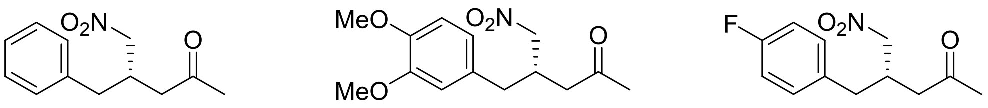

(*S*)-**4fa**, 22%
$Yield_{Met}$ = 71%
$Conv_{Org}$ = 36%

(*S*)-**4ga**, 24%
$Yield_{Met}$ = 71%
$Conv_{Org}$ = 38%

(*S*)-**4ha**, 26%
$Yield_{Met}$ = 57%
$Conv_{Org}$ = 53%

Scheme S1. *One-pot* sequential process to access chiral 1,4-nitro ketones (*S*)-**4ba-da** from allylbenzene derivatives **1f-h** combining Hoveyda-Grubbs catalyst **VIII** and chiral thiourea (*R*,*R*)-**IX**. Yields are for the isolated yield of the corresponding enone **3fa-ha**. Conversion of the organocatalytic step was determined by ^{1}H NMR.

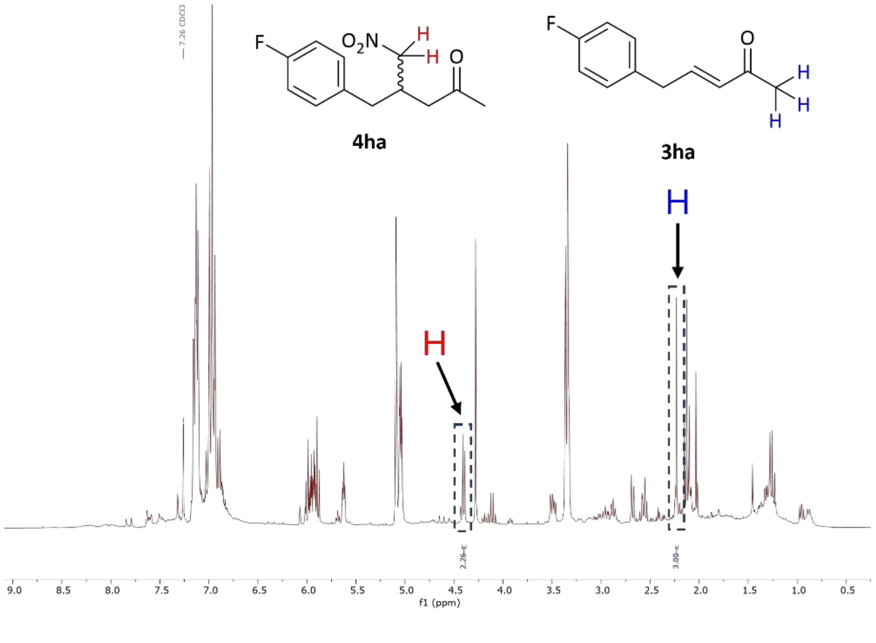

Figure S2. Illustrative example of the determination of conversion in the organocatalytic step for the synthesis of chiral 1,4-nitroketone **4ha**.

(*S*)-4-(3,4-dimethoxybenzyl)-5-nitropentan-2-one (4ga)

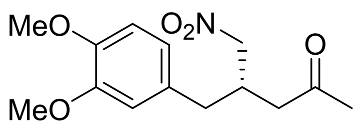

Following the general procedure and purification using a mixture of *n*-hexane and EtOAc in an 8:2 ratio as eluent, (*S*)-**4ga** was obtained as a pale brown oil, with a yield of 24% (34 mg). ^{1}**H NMR** (300 MHz, $CDCl_3$): δ 6.79 (d, *J* = 8.6 Hz, 1H), 6.70 (dq, *J* = 3.8, 2.0 Hz, 2H), 4.41 (d, *J* = 5.7 Hz, 2H), 3.86 (d, *J* = 4.3 Hz, 6H), 2.96 – 2.75 (m, 1H), 2.64 (d, *J* = 7.5 Hz, 2H), 2.56 (t, *J* = 6.1 Hz, 2H), 2.13 (s, 3H). ^{13}**C{^{1}H}-APT NMR** (75 MHz, $CDCl_3$): δ 206.7, 149.3, 148.1, 130.5, 121.4, 112.3, 111.5, 77.7, 56.0 (2H), 44.0, 37.1, 35.2, 30.6. The corresponding e.r. (2:98) was determined by chiral HPLC analysis of the product (Daicel Chiralpak IA column, *n*-hexane/ isopropyl alcohol 90:10, flow rate 1 mL min^{-1}, λ = 241.3 nm): t_{major} = 16.2 min; t_{minor} = 15.1 min. **HRMS** (ESI+) m/z: $[M+Na]^+$ Calcd for $[C_{14}H_{19}NNaO_5]^+$ 304.1155; found 304.1144.

(*S*)-4-(4-fluorobenzyl)-5-nitropentan-2-one (4ha)

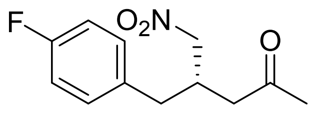

Following the general procedure and purification using a mixture of *n*-hexane and EtOAc in an 8:2 ratio as eluent, (*S*)-**4ha** was obtained as a pale brown oil, with a yield of 26% (31 mg). ^{1}**H NMR** (300 MHz, $CDCl_3$): δ 7.16 (dd, *J* = 8.6, 5.4 Hz, 2H), 7.06 – 6.95 (m, 2H), 4.41 (d, *J* = 5.6 Hz, 2H), 2.87 (dddd, *J* = 12.6, 8.1, 5.7, 1.5 Hz, 1H), 2.69 (d, *J* = 7.5 Hz, 2H), 2.56 (dd, *J* = 9.0, 6.4 Hz, 2H), 2.14 (s, 3H). ^{13}**C{^{1}H}-APT NMR** (75 MHz, CDCl3): δ 206.5, 162.0 (d, J = 245.4 Hz), 133.7 (d, J = 3.3 Hz), 130.8 (d, J = 7.9 Hz), 115.8 (d, J = 21.3 Hz), 77.6, 43.9, 36.7, 35.2, 30.6. The corresponding e.r. (>99:1) was determined by chiral HPLC analysis of the product (Daicel Chiralpak IC column, *n*-hexane/ isopropyl alcohol 90:10, flow rate 1 mL min^{-1}, λ = 250.0 nm): t_{major} = 18.2 min. **HRMS** (ESI+) m/z: $[M+Na]^+$ Calcd for $[C_{12}H_{14}FNNaO_3]^+$ 262.0850; found 262.0851.

7. General procedure for the synthesis of chiral nitroketones (*R*)-4aa-ea

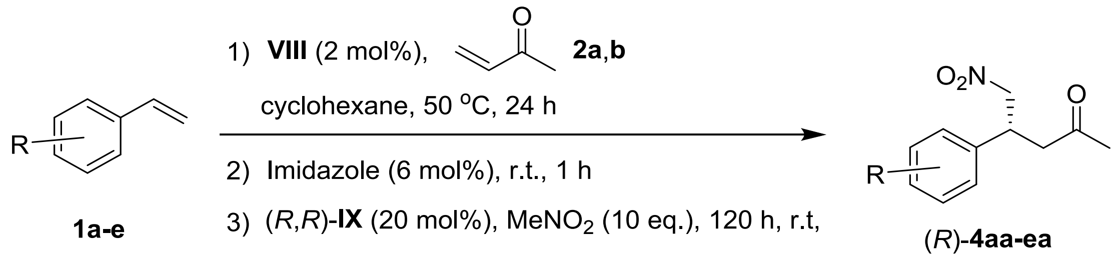

To a test tube containing a mixture of catalyst **VIII** (0.005 mmol, 0.02 equiv.) in cyclohexane (1 mL), MVK **2a** (0.25 mmol, 1.00 equiv.) and the corresponding styrene derivative **1a**–**e** (0.375 mmol, 1.5 equiv.) were added. The mixture was stirred at 50 °C in an oil bath for 4 hours. After this time, a second portion of the styrene derivative (0.375 mmol, 1.5 equiv.) was added, and the reaction allowed to proceed for additional 24 hours. The reaction mixture was then brought to room temperature, and once this temperature was reached, imidazole (0.03 mmol, 0.06 mol%) was added. Stirring was continued for 1 hour. Subsequently, the organocatalyst (*R*,*R*)-**IX** (0.1 mmol, 0.2 equiv.) and $MeNO_2$ (5 mmol, 10 equiv.) were added, and the reaction stirred at room temperature for 120 hours. Afterwards, the mixture was purified by column chromatography using a mixture of *n*-hexane and EtOAc in an 8:2 ratio as eluent, affording the corresponding products (*R*)-**4aa-ea**.

(*R*)-5-nitro-4-(m-tolyl)pentan-2-one (4aa)

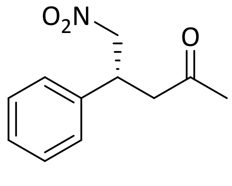

Following the general procedure and purification using a mixture of *n*-hexane and EtOAc in an 8:2 ratio as eluent, (*R*)-**4aa** was obtained as a pale brown oil, with a yield of 85% (44 mg). **^{1}H NMR** (300 MHz, $CDCl_3$): δ 7.38 – 7.17 (m, 5H), 4.73 – 4.55 (m, 2H), 4.00 (p, J = 7.1 Hz, 1H), 2.91 (d, J = 7.0 Hz, 2H), 2.11 (s, 3H). **^{13}C{^{1}H}-APT NMR** (75 MHz, $CDCl_3$) δ 205.5, 138.9, 129.2, 128.0, 127.5, 79.6, 46.3 39.2, 30.5. The corresponding e.r. (98:2) was determined by chiral HPLC analysis of the product (Phenomenex i-Amylose-1 column, *n*-hexane/isopropyl alcohol 95:5, flow rate 1 mL min^{-1}, λ = 221.5 nm): t_{major} = 18.4 min; t_{minor} = 17.3 min. HRMS (ESI$^+$) m/z: [M+Na]$^+$ Calcd for $[C_{11}H_{13}NNaO_3]^+$ 230.0799 [M+Na]$^+$; found 230.0799.

(*R*)-5-nitro-4-(m-tolyl)pentan-2-one (4ba)

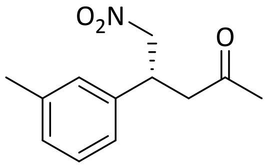

Following the general procedure and purification using a mixture of *n*-hexane and EtOAc in an 8:2 ratio as eluent, (*R*)-**4ba** was obtained as a pale brown oil, with a yield of 89% (49 mg). **^{1}H NMR** (300 MHz, $CDCl_3$): δ 7.20 (t, J = 7.5 Hz, 1H), 7.09 – 6.97 (m, 3H), 4.71 – 4.53 (m, 2H), 3.96 (p, J = 7.1 Hz, 1H), 2.89 (d, J = 7.0 Hz, 2H), 2.32 (s, 3H), 2.10 (s, 3H). **^{13}C{^{1}H}-APT NMR** (101 MHz, $CDCl_3$): δ 205.6, 138.9 (2H), 129.0, 128.7, 128.3, 124.3, 79.6, 46.3, 39.1, 30.4, 21.5. The corresponding e.r. (97:3) was determined by chiral HPLC analysis of the product (Phenomenex i-Amylose-1 column, *n*-heptane/isopropyl alcohol 95:5, flow rate 1 mL min^{-1}, λ = 229.4 nm): t_{major} = 15.1 min; t_{minor} = 14.0 min. HRMS (ESI$^+$) m/z: [M+Na]$^+$ Calcd for $[C_{12}H_{15}NNaO_3]^+$ 244.0944 [M+Na]$^+$; found 244.0944.

(*R*)-4-(4-methoxyphenyl)-5-nitropentan-2-one (4ca)

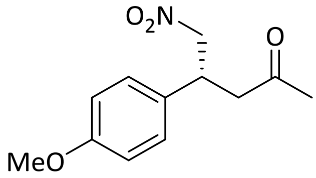

Following the general procedure and purification using a mixture of *n*-hexane and EtOAc in an 8:2 ratio as eluent, (*R*)-**4ca** was obtained as a pale brown oil, with a yield of 68% (40 mg). **^{1}H NMR** (300 MHz, $CDCl_3$): δ 7.17 – 7.08 (m, 2H), 6.88 – 6.80 (m, 2H), 4.71 – 4.49 (m, 2H), 3.95 (p, J = 7.1 Hz, 1H), 3.77 (s, 3H), 2.87 (d, J = 7.1 Hz, 2H), 2.10 (s, 3H). **^{13}C{^{1}H}-APT NMR** (101 MHz, $CDCl_3$): δ 205.7, 159.2, 130.8, 128.6, 114.5, 79.8, 55.4, 46.4, 38.5, 30.5. The corresponding e.r. (97:3) was determined by chiral HPLC analysis of the product (Daicel Chiralpak IC column, *n*-hexane/ isopropyl alcohol 95:5, flow rate 1 mL min^{-1}, λ = 249.7 nm): t_{major} = 30.0 min; t_{minor} = 26.6 min. **HRMS** (ESI+) m/z: [M+Na]$^+$ Calcd for $[C_{12}H_{15}NNaO_4]^+$ 260.0893; found 260.0893.

(*R*)-5-nitro-4-(3-nitrophenyl)pentan-2-one (4da)

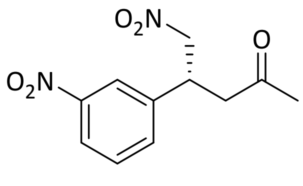

Following the general procedure and purification using a mixture of *n*-hexane and EtOAc in an 8:2 ratio as eluent, (*R*)-**4da** was obtained as a pale brown oil, with a yield of 71% (45 mg). **^{1}H NMR** (400 MHz, $CDCl_3$): δ 8.15 – 8.08 (m, 2H), 7.67 – 7.49 (m, 2H), 4.80 – 4.61 (m, 2H), 4.14 (dt, J = 15.0, 7.1 Hz, 1H), 2.98 (d, J = 6.9 Hz, 1H), 2.15 (s, 3H). **^{13}C{^{1}H}-APT NMR** (101 MHz, $CDCl_3$): δ 204.7, 148.6, 141.3, 134.4, 130.2, 123.1, 122.3, 78.8, 45.8, 38.6, 30.4. The corresponding e.r. (97:3) was determined by chiral HPLC analysis of the product (Daicel Chiralpak IC column, *n*-hexane/ isopropyl alcohol 90:10, flow rate 1 mL min^{-1}, λ = 258.3 nm): t_{major} = 34.4 min; t_{minor} = 38.5 min. **HRMS** (ESI+) m/z: [M+Na]$^+$ Calcd for $[C_{11}H_{12}N_2NaO_5]^+$ 275.0638; found 275.0638.

(*R*)-4-(1-nitro-4-oxopentan-2-yl)phenyl acetate (4ea)

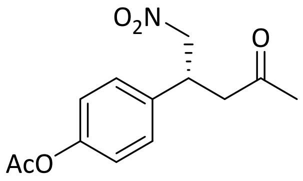

Following the general procedure and purification using a mixture of *n*-hexane and EtOAc in an 8:2 ratio as eluent, (*R*)-**4ea** was obtained as a pale brown oil, with a yield of 55% (36 mg). **^{1}H NMR** (400 MHz, $CDCl_3$): δ 7.23 – 7.16 (m, 2H), 7.05 – 6.99 (m, 2H), 4.68 – 4.50 (m, 2H), 3.98 (dt, *J* = 13.8, 7.1 Hz, 1H), 2.86 (d, *J* = 7.0 Hz, 2H), 2.25 (s, 3H), 2.07 (s, 3H). **^{13}C{^{1}H}-APT NMR** (101 MHz, $CDCl_3$): δ 205.3, 169.3, 150.1, 136.5, 128.5, 122.2, 79.3, 46.1, 38.4, 30.3, 21.1. The corresponding e.r. (96:4) was determined by chiral HPLC analysis of the product (Phenomenex i-Amylose-1 column, *n*-heptane/isopropyl alcohol 90:10, flow rate 1 mL min^{-1}, λ =252.3 nm): t_{major} = 48.9 min; t_{minor} = 42.1 min. **HRMS** (ESI+) m/z: [M+Na]$^+$ Calcd for $[C_{13}H_{15}NNaO_5]^+$ 288.0842; found 288.0842.

8. General procedure for the synthesis of chiral 1,4-nitroalcohols 5aa, 5ab y 5ha using Evo200 enzyme

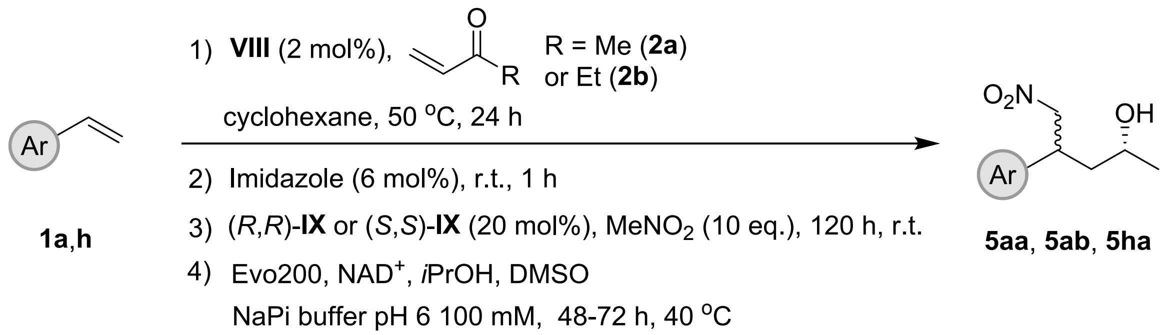

To a test tube containing a mixture of catalyst **VIII** (0.01 mmol, 0.02 equiv.) in cyclohexane (1 mL), vinyl ketone MVK **2a** or EVK **2b** (0.5 mmol, 1.00 equiv.) and the corresponding styrene derivative **1a** or **1h** (0.75 mmol, 1.5 equiv. for **1a** or 0.5 mmol, 1 equiv. for **1h**) were added. The mixture was stirred at 50 °C in an oil bath for 4 hours. After this time, a second portion of the styrene derivative (0.75 mmol, 1.5 equiv. for **1a** or 0.5 mmol, 1 equiv. for **1h**) was added, and the reaction allowed to proceed for an additional 24 hours. The reaction mixture was then brought to room temperature, and once this temperature was reached, imidazole (0.03 mmol, 0.06 equiv.) was added. Stirring was continued for 1 hour. Subsequently, the organocatalyst (*R*,*R*)-**IX** or (*S*,*S*)-**IX** (0.1 mmol, 0.2 equiv.) and $MeNO_2$ (5 mmol, 10 equiv.) were added, and the reaction is stirred at room temperature for 120 hours. Finally, *i*PrOH (250 µL, 5% v/v), DMSO (500 µL, 10% v/v), ADH Evo200 (100 µL, 10 mg/mL), NADH (10 µL, 50 mM), and 4.14 mL of 100 mM NaPi buffer pH 6 were added. The reaction was stirred with orbital shaking at 40 °C for 24 h. Successive additions of enzyme and cofactor were made every 24 h until no nitro ketone was observed in the crude reaction mixture by NMR. The reaction mixture was then purified by column chromatography using a mixture of *n*-hexane and EtOAc in an 8:2 ratio, affording the corresponding products **5aa**, **5ab** and **5ha**.

(2*R*,4*R*)-5-nitro-4-phenylpentan-2-ol (5aa)

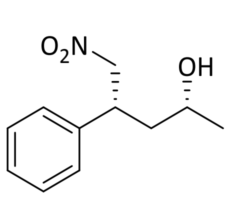

Following the general procedure and purification using a mixture of *n*-hexane and EtOAc in an 8:2 ratio as eluent, (2*R*,4*R*)-**5aa** was obtained as a pale brown oil, with a yield of 76% (79 mg). **^{1}H NMR** (300 MHz, $CDCl_3$): δ 7.40 – 7.15 (m, 5H), 4.72 – 4.50 (m, 2H), 3.88 – 3.72 (m, 1H), 3.65 – 3.48 (m, 1H), 1.88 – 1.66 (m, 2H), 1.50 (bs, 1H), 1.16 (d, *J* = 6.2 Hz, 3H). **^{13}C{^{1}H}-APT NMR** (75 MHz, $CDCl_3$): δ 139.4, 129.2, 127.9, 127.6, 80.6, 65.8, 42.3, 41.5, 23.6. **HRMS** (ESI+) m/z: [M+Na]$^+$ Calcd for $[C_{11}H_{15}NNaO_3]^+$ 232.0955; found 232.0944.

(2*R*,4*S*)-5-nitro-4-phenylpentan-2-ol (5aa)

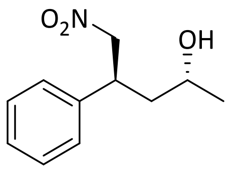

Following the general procedure and purification using a mixture of *n*-hexane and EtOAc in an 8:2 ratio as eluent, (2*R*,4*S*)-**5aa** was obtained as a pale brown oil, with a yield of 54% (56 mg). **^{1}H NMR** (300 MHz, $CDCl_3$): δ 7.34 – 7.11 (m, 5H), 4.65 – 4.47 (m, 2H), 3.71 – 3.51 (m, 2H), 1.96 – 1.65 (m, 3H), 1.13 (d, *J* = 6.2 Hz, 3H). **^{13}C{^{1}H}-APT NMR** (75 MHz, $CDCl_3$): δ 139.4, 129.2, 127.9, 127.6, 65.8, 42.3, 41.5, 23.6. **HRMS** (ESI+) m/z: $[M+Na]^+$ Calcd for $[C_{11}H_{15}NNaO_3]^+$ 232.0955; found 232.0944.

(3*R*,5*R*)-6-nitro-phenylhexan-3-ol (5ab)

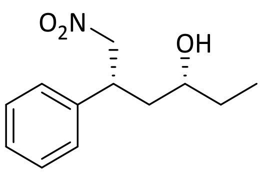

Following the general procedure and purification using a mixture of *n*-hexane and EtOAc in an 8:2 ratio as eluent, (3*R*,5*R*)-**5ab** was obtained as a pale brown oil, with a yield of 50% (56 mg). **^{1}H NMR** (400 MHz, $CDCl_3$): δ 7.26 – 7.08 (m, 5H), 4.61 – 4.43 (m, 2H), 3.79 – 3.66 (m, 1H), 3.17 (dtd, *J* = 9.9, 6.2, 2.4 Hz, 1H), 1.80 – 1.55 (m, 3H), 1.32 (td, *J* = 7.7, 6.2 Hz, 2H), 0.76 (t, *J* = 7.4 Hz, 3H). **^{13}C{^{1}H}-APT NMR** (101 MHz, $CDCl_3$): δ 139.4, 129.1, 127.8, 81.2, 70.4, 40.1, 31.2, 9.8. **HRMS** (ESI+) m/z: $[M+Na]^+$ Calcd for $[C_{12}H_{17}NNaO_3]^+$ 246.1101; found 246.1101.

(3*R*,5*S*)-6-nitro-phenylhexan-3-ol (5ab)

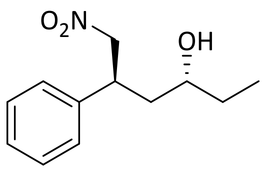

Following the general procedure and purification using a mixture of *n*-hexane and EtOAc in an 8:2 ratio as eluent, (3*R*,5*S*)-**5ab** was obtained as a pale brown oil, with a yield of 42% (47 mg). **^{1}H NMR** (400 MHz, $CDCl_3$): δ 7.26 – 7.09 (m, 5H), 4.65 – 4.47 (m, 2H), 3.59 (dq, *J* = 8.6, 7.1 Hz, 1H), 3.41 (tdd, *J* = 7.2, 5.7, 4.5 Hz, 1H), 1.77 (dd, *J* = 6.6, 5.7 Hz, 2H), 1.50 – 1.27 (m, 3H), 0.81 (t, *J* = 7.4 Hz, 3H). **^{13}C{^{1}H}-APT NMR** (101 MHz, $CDCl_3$): δ 139.8, 129.1, 127.8, 127.5, 80.4, 41.3, 40.2, 30.1, 9.6. **HRMS** (ESI+) m/z: $[M+Na]^+$ Calcd for $[C_{12}H_{17}NNaO_3]^+$ 246.1101; found 246.1101.

(2*R*,4*S*)-4-(4-fluorobenzyl)-5-nitropentan-2-ol (5ha)

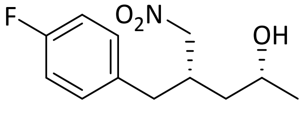

Following the general procedure and purification using a mixture of *n*-hexane and EtOAc in an 8:2 ratio as eluent, (2*R*,4*S*)-**5ab** was obtained as a pale brown oil, with a yield of 28% (34 mg). **^{1}H NMR** (400 MHz, $CDCl_3$): δ 7.19 – 7.11 (m, 2H), 7.03 – 6.97 (m, 2H), 4.45 (dd, *J* = 12.4, 5.9 Hz, 1H), 4.31 (dd, *J* = 12.4, 6.3 Hz, 1H), 3.95 – 3.87 (m, 1H), 2.80 – 2.58 (m, 3H), 1.64 – 1.55 (m, 1H), 1.53 – 1.43 (m, 2H), 1.20 (d, *J* = 6.1 Hz, 3H). **^{13}C{^{1}H}-APT NMR** (75 MHz, $CDCl_3$): δ 163.5, 160.3, 134.1, 130.8, 115.5, 79.1, 66.3, 40.5, 37.36, 37.2, 25.0. **HRMS** not obtainable.

(2*R*,4*R*)-4-(4-fluorobenzyl)-5-nitropentan-2-ol (5ha)

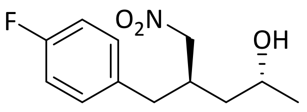

Following the general procedure and purification using a mixture of *n*-hexane and EtOAc in an 8:2 ratio as eluent, (2*R*,4*R*)-**5ab** was obtained as a pale brown oil, with a yield of 24% (29 mg). **^{1}H NMR** (400 MHz, $CDCl_3$): δ 7.19 – 7.13 (m, 2H), 7.02 – 6.97 (m, 2H), 4.47 – 4.35 (m, 2H), 4.03 – 3.94 (m, 1H), 2.77 – 2.63 (m, 3H), 1.59 – 1.45 (m, 3H), 1.20 (d, *J* = 6.1 Hz, 3H). **^{13}C{^{1}H}-APT NMR** (75 MHz, $CDCl_3$): δ 163.5, 160.2, 134.2, 130.8, 115.5, 78.2, 65.33, 40.1, 37.7, 36.5, 24.6. **HRMS** not obtainable.

9. Procedure for the scale-up synthesis of chiral 1,4-nitroalcohol (2*R*,4*R*)-5aa

To a test tube containing a mixture of catalyst **VIII** (0.02 mmol, 0.02 equiv.) in cyclohexane (2 mL), MVK **2a** (1 mmol, 1.00 equiv.) and styrene **1a** (1.5 mmol, 1.5 equiv.) were added. The mixture was stirred at 50 °C in an oil bath for 4 hours. After this time, a second portion of **1a** (1.5 mmol, 1.5 equiv.) was added, and the reaction allowed to proceed for an additional 24 hours. The reaction mixture was then brought to room temperature, and once this temperature was reached, imidazole (0.06 mmol, 0.06 equiv.) was added. Stirring continued for 1 hour. Subsequently, the organocatalyst (*R*,*R*)-**IX** (0.2 mmol, 0.2 equiv.) and $MeNO_2$ (10 mmol, 10 equiv.) were added, and the reaction stirred at room temperature for 120 hours. After that, to facilitate the enzymatic reaction, solvents were evaporated. Then, *i*PrOH (500 µL, 5% v/v), DMSO (1 mL, 10% v/v), ADH Evo200 (200 µL, 10 mg/mL), NADH (20 µL, 50 mM), and 8.28 mL of 100 mM NaPi buffer pH 6 were added. Successive additions of enzyme and cofactor were made every 24 h until no nitro ketone was observed in the crude reaction mixture by NMR (13 mg total enzyme). The reaction was stirred with orbital shaking at 40 °C for 24 h. The reaction mixture was then purified by column chromatography using a mixture of *n*-hexane and EtOAc in an 8:2 ratio, affording the corresponding product (2*R*,4*R*)-**5aa** with an isolated yield of 43% (90 mg). ([α]= + 0.44; c 0.8 $CHCl_3$)

10. General procedure for the synthesis of chiral 1,4-nitroalcohols 5aa, 5ab y 5ha using ADH-A

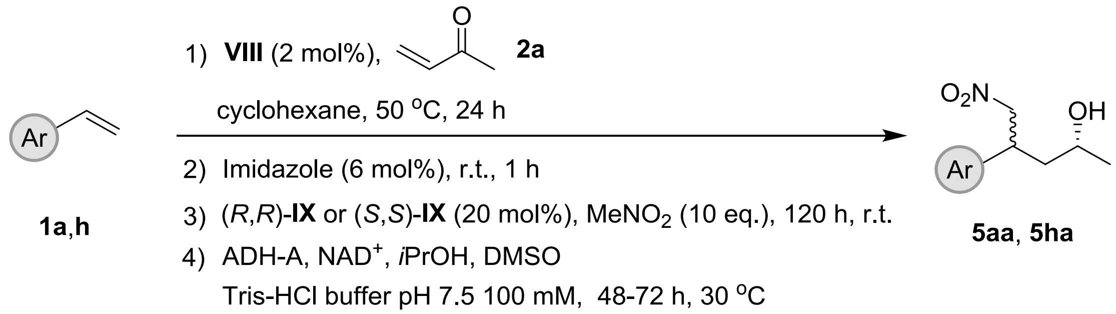

To a test tube containing a mixture of catalyst **VIII** (0.01 mmol, 0.02 equiv.) in cyclohexane (1 mL), MVK **2a** (0.5 mmol, 1.00 equiv.) and the corresponding styrene derivative **1a** or **1h** (0.75 mmol, 1.5 equiv. for **1a** or 0.5 mmol, 1 equiv. for **1h**) were added. The mixture was stirred in an oil bath at 50 °C for 4 hours. After this time, a second portion of the styrene derivative (0.75 mmol, 1.5 equiv. for **1a** or 0.5 mmol, 1 equiv. for **1h**) was added, and the reaction allowed to proceed for an additional 24 hours. The reaction mixture was then brought to room temperature, and once this temperature was reached, imidazole (0.03 mmol, 0.06 equiv.) was added. Stirring was continued for 1 hour. Subsequently, the organocatalyst (*R*,*R*)-**IX** or (*S*,*S*)-**IX** (0.1 mmol, 0.2 equiv.) and $MeNO_2$ (5 mmol, 10 equiv.) were added, and the reaction is stirred at room temperature for 120 hours. Finally, *i*PrOH (750 µL, 5% v/v), DMSO (1.5 mL, 10% v/v), ADH as CFE (150 mg, 10 mg/mL solution), NADH (10 µL, 50 mM), and 12.7 mL of 100 mM Tris-HCl pH 7.5 were added. Successive additions of enzyme and cofactor were made every 24 h until no nitro ketone was observed in the crude reaction mixture by NMR. The reaction was stirred with orbital shaking at 40 °C for 24 h. The reaction mixture was then purified by column chromatography using a mixture of *n*-hexane and EtOAc in an 8:2 ratio, affording the corresponding products **5aa** and **5ha**.

(2*S*,4*R*)-5-nitro-4-phenylpentan-2-ol (5aa)

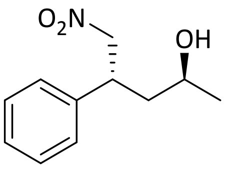

Following the general procedure and purification using a mixture of *n*-hexane and EtOAc in an 8:2 ratio as eluent, (2*S*,4*R*)-**5aa** was obtained as a pale brown oil, with a yield of 45% (47 mg). **^{1}H NMR** (400 MHz, $CDCl_3$): δ 7.33 – 7.12 (m, 5H), 4.65 – 4.48 (m, 2H), 3.71 – 3.51 (m, 2H), 1.99 – 1.56 (m, 3H), 1.13 (d, *J* = 6.2 Hz, 3H). **^{13}C{^{1}H}-APT NMR** (101 MHz, $CDCl_3$): δ 139.4, 129.2 (2C), 127.9, 127.6 (2C), 80.5, 65.7, 42.3, 41.5, 23.5. **HRMS** (ESI+) m/z: $[M+Na]^+$ Calcd for $[C_{11}H_{15}NNaO_3]^+$ 232.0955; found 232.0944.

(2*S*,4*S*)- 5-nitro-4-phenylpentan-2-ol (5aa)

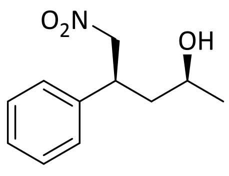

Following the general procedure and purification using a mixture of *n*-hexane and EtOAc in an 8:2 ratio as eluent, (2*S*,4*S*)-**5aa** was obtained as a pale brown oil, with a yield of 62% (65 mg). **^{1}H NMR** (400 MHz, $CDCl_3$): δ 7.34 – 7.11 (m, 5H), 4.66 – 4.46 (m, 2H), 3.79 – 3.66 (m, 1H), 3.49 (m, 1H), 1.80 – 1.61 (m, 3H), 1.09 (d, *J* = 6.2 Hz, 3H). **^{13}C{^{1}H}-APT NMR** (101 MHz, $CDCl_3$): δ 139.3, 129.1 (2C), 127.8 (3C), 81.1, 65.3, 42.3, 41.5, 24.7. **HRMS** (ESI+) m/z: $[M+Na]^+$ Calcd for $[C_{11}H_{15}NNaO_3]^+$ 232.0955; found 232.0944.

(2*S*,4*S*)-4-(4-fluorobenzyl)-5-nitropentan-2-ol (5ha)

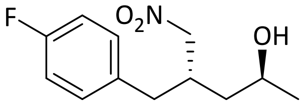

Following the general procedure and purification using a mixture of *n*-hexane and EtOAc in an 8:2 ratio as eluent, (2*S*,4*S*)-**5ha** was obtained as a pale brown oil, with a yield of 27% (32 mg). **^{1}H NMR** (400 MHz, $CDCl_3$): δ 7.19 – 7.13 (m, 2H), 7.02 – 6.97 (m, 2H), 4.47 – 4.35 (m, 2H), 4.03 – 3.94 (m, 1H), 2.77 – 2.63 (m, 3H), 1.59 – 1.45 (m, 3H), 1.20 (d, *J* = 6.1 Hz, 3H). **^{13}C{^{1}H}-APT NMR** (75 MHz, $CDCl_3$): δ 163.5, 160.2, 134.2, 130.8, 115.5, 78.2, 65.33, 40.1, 37.7, 36.5, 24.6. **HRMS** not obtainable.

(2*S*,4*R*)-4-(4-fluorobenzyl)-5-nitropentan-2-ol (5ha)

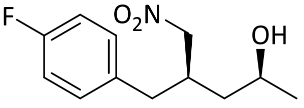

Following the general procedure and purification using a mixture of *n*-hexane and EtOAc in an 8:2 ratio as eluent, (2*S*,4*R*)-**5ha** was obtained as a pale brown oil, with a yield of 22% (26 mg). **^{1}H NMR** (400 MHz, $CDCl_3$): δ 7.19 – 7.11 (m, 2H), 7.03 – 6.97 (m, 2H), 4.45 (dd, *J* = 12.4, 5.9 Hz, 1H), 4.31 (dd, *J* = 12.4, 6.3 Hz, 1H), 3.95 – 3.87 (m, 1H), 2.80 – 2.58 (m, 3H), 1.64 – 1.55 (m, 1H), 1.53 – 1.43 (m, 2H), 1.20 (d, *J* = 6.1 Hz, 3H). **^{13}C{^{1}H}-APT NMR** (75 MHz, $CDCl_3$): δ 163.5, 160.3, 134.1, 130.8, 115.5, 79.1, 66.3, 40.5, 37.36, 37.2, 25.0. **HRMS** not obtainable.

11. General procedure for the synthesis of racemic 1,4-nitroalcohols 5ab and 5ha

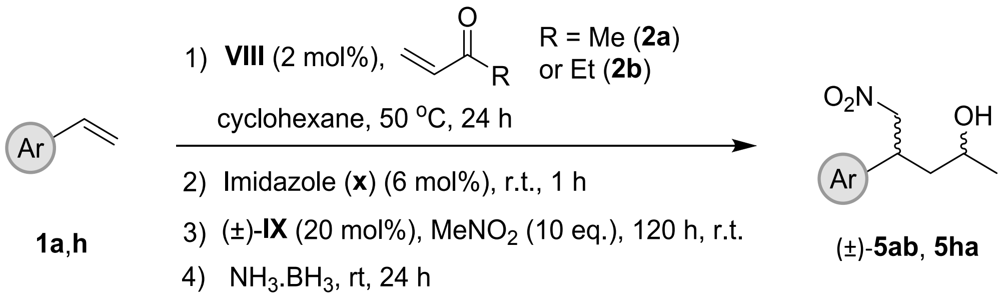

To a test tube containing a mixture of catalyst **VIII** (0.005 mmol, 0.02 equiv.) in cyclohexane (1 mL), vinyl ketone MVK **2a** or EVK **2b** (0.25 mmol, 1.00 equiv.) and the corresponding styrene derivative **1a** or **1h** (0.75 mmol, 1.5 equiv. for 1a or 0.5 mmol, 1 equiv. for 1h) were added. The mixture was stirred at 50 °C in an oil bath for 4 hours. After this time, a second portion of the styrene derivative (0.375 mmol, 1.5 equiv.) was added, and the reaction allowed to proceed for an additional 24 hours. The reaction mixture was then brought to room temperature, and once this temperature was reached, imidazole (0.03 mmol, 0.06 mol%) was added. Stirring was continued for 1 hour. Subsequently, the organocatalyst (±)-**IX** (0.1 mmol, 0.2 equiv.) and $MeNO_2$ (5 mmol, 10 equiv.) were added, and the reaction stirred at room temperature for 120 hours. Finally, ammonia–borane ($NH_3{\cdot}BH_3$) (15.4 mg, 0.5 mmol, 1 equiv.) was added, and the reaction stirred at room temperature for 24 h. The reaction mixture was then purified by column chromatography using a mixture of *n*-hexane and EtOAc in an 8:2 ratio, affording the corresponding products **5ab** and **5ha**.

12. Chiral HPLC analysis of 1,4-nitroketones 4aa-4ea

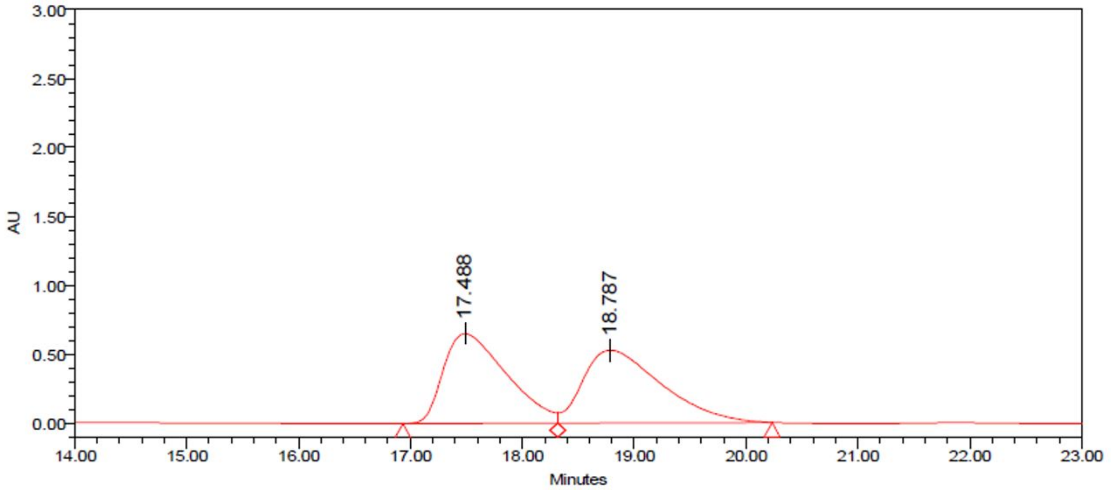

Processed Channel: PDA 221.5 nm

	Processed Channel	Retention Time (min)	Area	% Area	Height
1	PDA 221.5 nm	17.488	25378651	49.71	649715
2	PDA 221.5 nm	18.787	25676307	50.29	528043

Figure S3. Racemic mixture of **4aa**. Phenomenex i-Amylose-1 column (*n*-hexane/ethyl acetate = 95:5, 1 mL min^{-1}, λ = 221.5 nm).

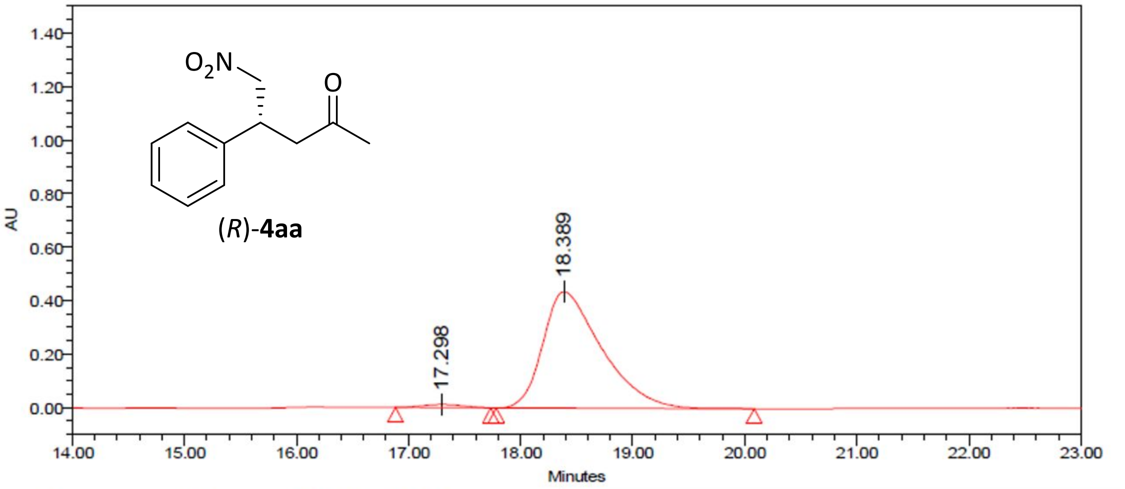

Processed Channel: PDA 221.5 nm

	Processed Channel	Retention Time (min)	Area	% Area	Height
1	PDA 221.5 nm	17.298	317729	1.96	12510
2	PDA 221.5 nm	18.389	15913944	98.04	436688

Figure S4. Enantioenriched mixture of (*R*)-**4aa** (2:98 e.r.). Phenomenex i-Amylose-1 column (*n*-hexane/ethyl acetate = 95:5, 1 mL min^{-1}, λ = 221.5 nm).

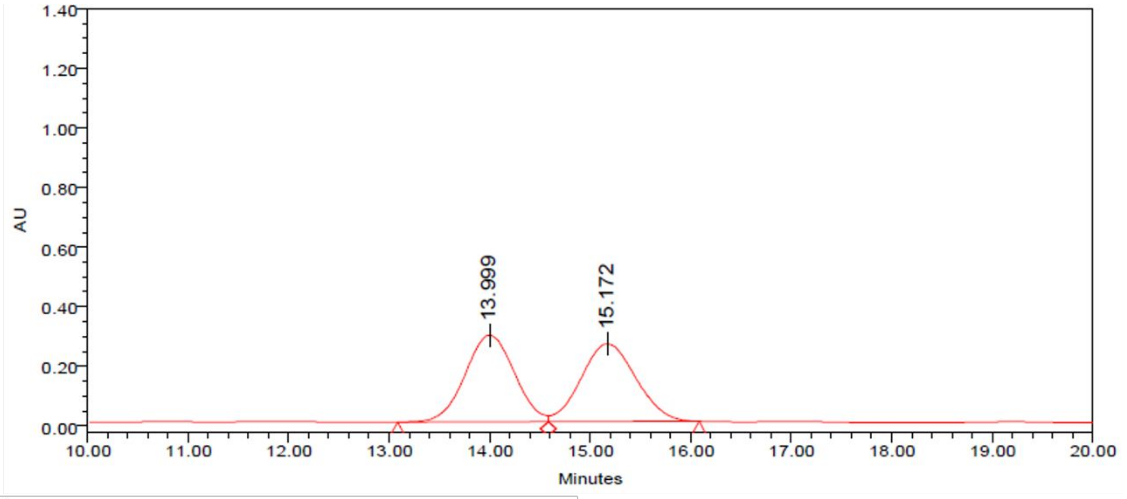

Processed Channel: PDA 229.4 nm

	Processed Channel	Retention Time (min)	Area	% Area	Height
1	PDA 229.4 nm	13.999	9907881	50.02	290845
2	PDA 229.4 nm	15.172	9901248	49.98	261094

Figure S5. Racemic mixture of **4ba**. Phenomenex i-Amylose-1 column (*n*-heptane/ethyl acetate = 95:5, 1 mL min^{-1}, λ = 229.4 nm).

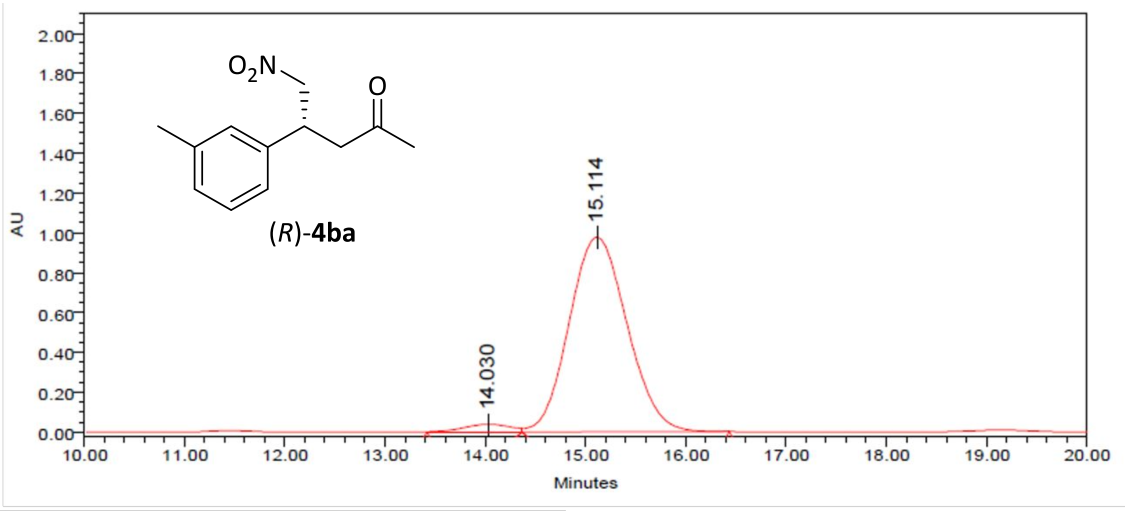

Processed Channel: PDA 227.2 nm

	Processed Channel	Retention Time (min)	Area	% Area	Height
1	PDA 227.2 nm	14.030	1179531	2.93	37500
2	PDA 227.2 nm	15.114	39109644	97.07	975779

Figure S6. Enantioenriched mixture of (*R*)-**4ba** (3:97 e.r.). Phenomenex i-Amylose-1 column (*n*-heptane/ethyl acetate = 95:5, 1 mL min^{-1}, λ = 227.2 nm).

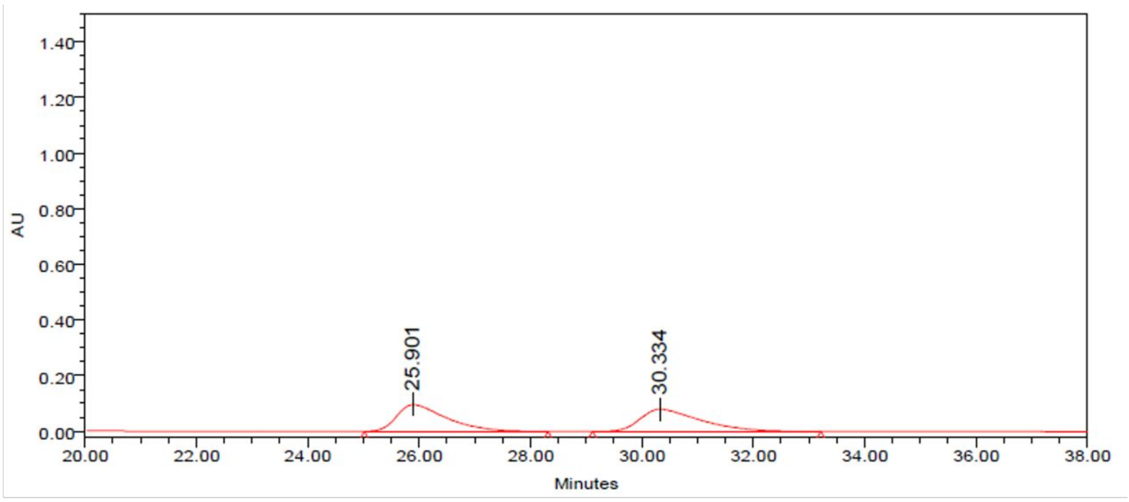

Processed Channel: PDA 249.7 nm

	Processed Channel	Retention Time (min)	Area	% Area	Height
1	PDA 249.7 nm	25.901	5801191	50.05	95544
2	PDA 249.7 nm	30.334	5790429	49.95	78702

Figure S7. Racemic mixture of **4ca**. Phenomenex i-Amylose-1 column (*n*-hexane/isopropyl alcohol = 95:5, 1 mL min^{-1}, λ = 249.7 nm).

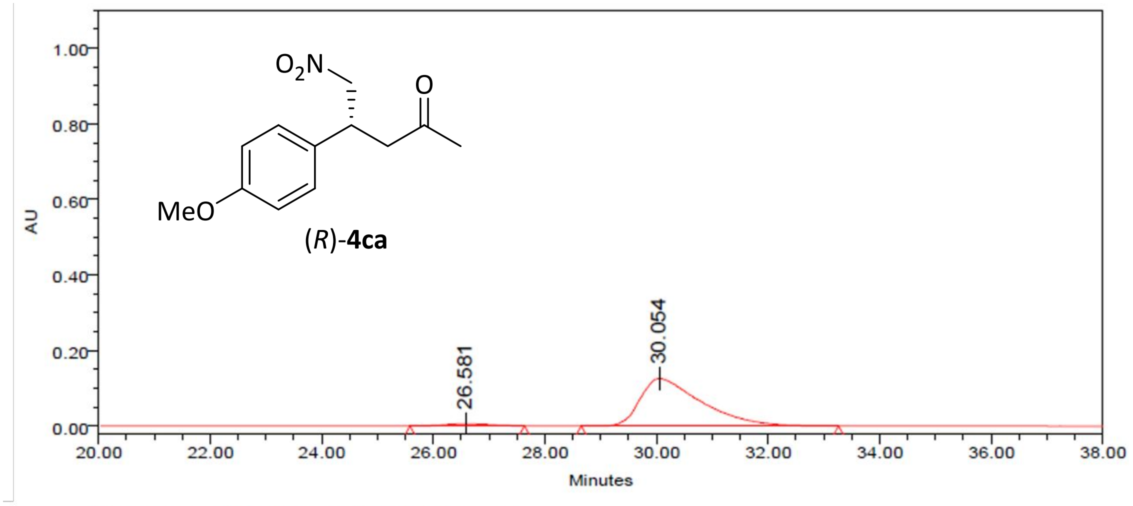

Processed Channel: PDA 249.7 nm

	Processed Channel	Retention Time (min)	Area	% Area	Height
1	PDA 249.7 nm	26.581	295838	3.02	5611
2	PDA 249.7 nm	30.054	9512388	96.98	125360

Figure S8. Enantioenriched mixture of (*R*)-**4ca** (3:97 e.r.). Phenomenex i-Amylose-1 column (*n*-hexane/isopropyl alcohol = 95:5, 1 mL min^{-1}, λ = 249.7 nm).

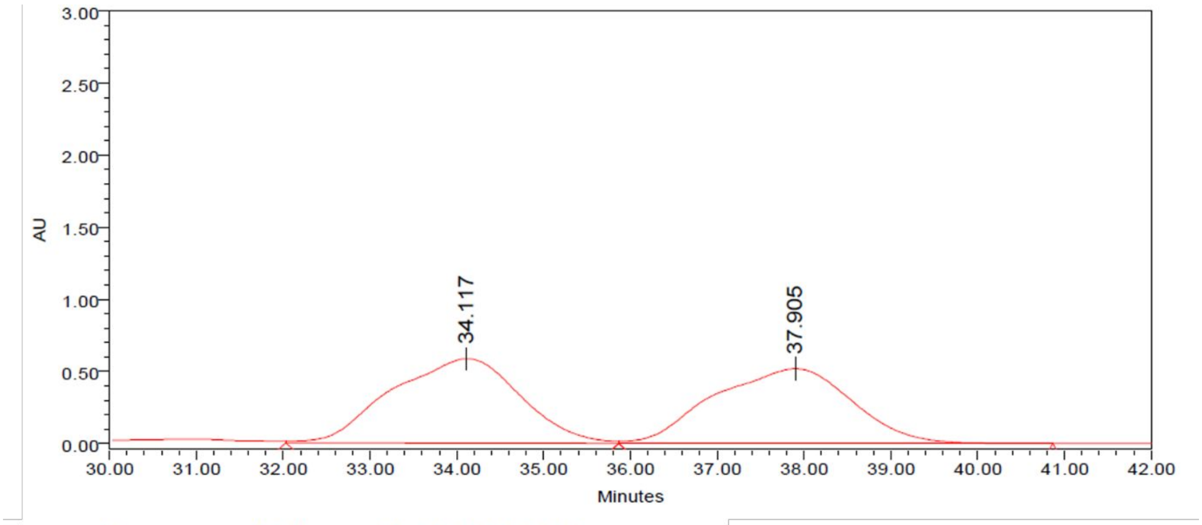

Processed Channel: PDA 258.7 nm

	Processed Channel	Retention Time (min)	Area	% Area	Height
1	PDA 258.7 nm	34.117	60526456	51.39	585578
2	PDA 258.7 nm	37.905	57262893	48.61	516667

Figure S9. Racemic mixture of **4da**. Daicel ChiralPak IC column (*n*-hexane/ethyl acetate = 90:10, 1 mL min^{-1}, λ = 258.7 nm).

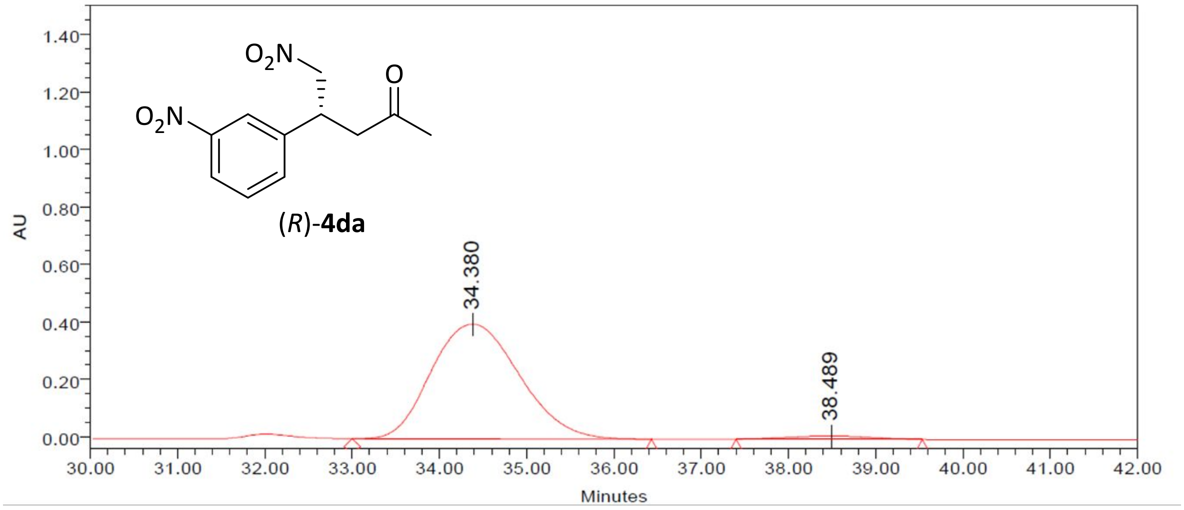

Processed Channel: PDA 258.3 nm

	Processed Channel	Retention Time (min)	Area	% Area	Height
1	PDA 258.3 nm	34.380	28131330	97.31	399459
2	PDA 258.3 nm	38.489	776387	2.69	11518

Figure S10. Enantioenriched mixture of (*R*)-**4da** (97:3 e.r.). Daicel ChiralPak IC column (*n*-hexane/ethyl acetate = 90:10, 1 mL min^{-1}, λ = 258.3 nm).

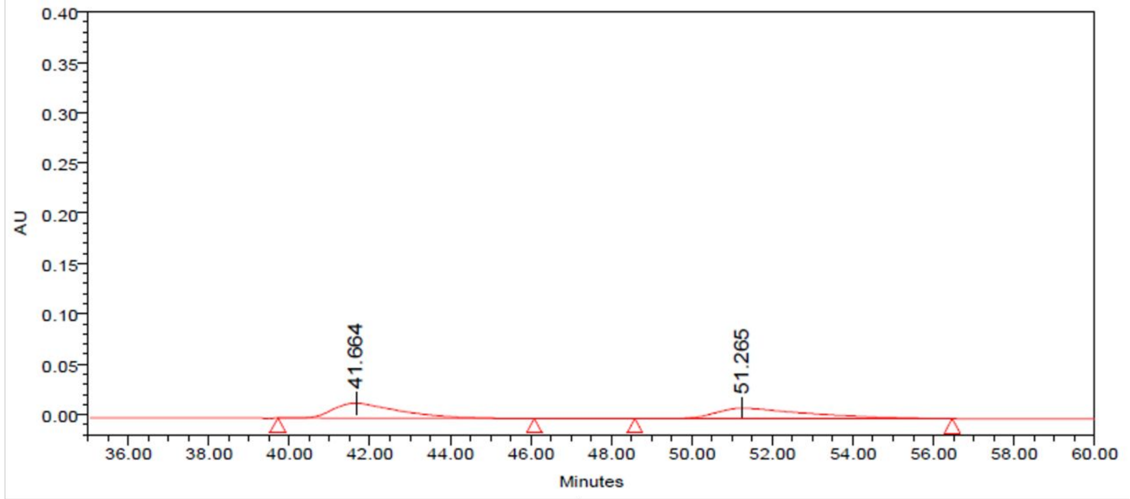

Processed Channel: PDA 252.3 nm

	Processed Channel	Retention Time (min)	Area	% Area	Height
1	PDA 252.3 nm	41.664	1684044	50.69	14887
2	PDA 252.3 nm	51.265	1638205	49.31	10266

Figure S11. Racemic mixture of **4ea**. Phenomenex i-Amylose-1 column (*n*-heptane/isopropyl alcohol = 90:10, 1 mL min^{-1}, λ = 252.3 nm).

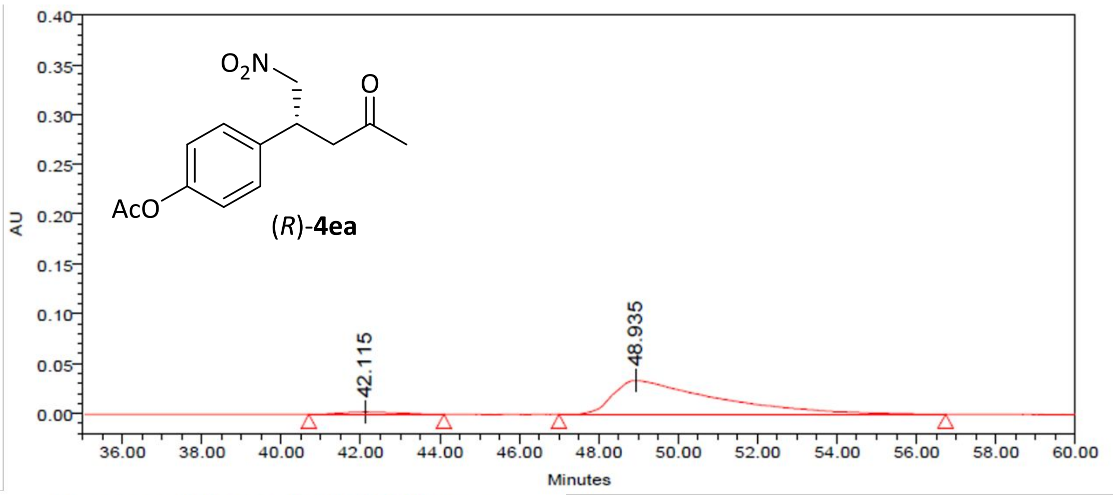

Processed Channel: PDA 252.3 nm

	Processed Channel	Retention Time (min)	Area	% Area	Height
1	PDA 252.3 nm	42.115	235305	3.71	2485
2	PDA 252.3 nm	48.935	6111432	96.29	34135

Figure S12. Enantioenriched mixture of (*R*)-**4ea** (4:96 e.r.). Phenomenex i-Amylose-1 column (*n*-heptane/isopropyl alcohol = 90:10, 1 mL min^{-1}, λ = 252.3 nm).

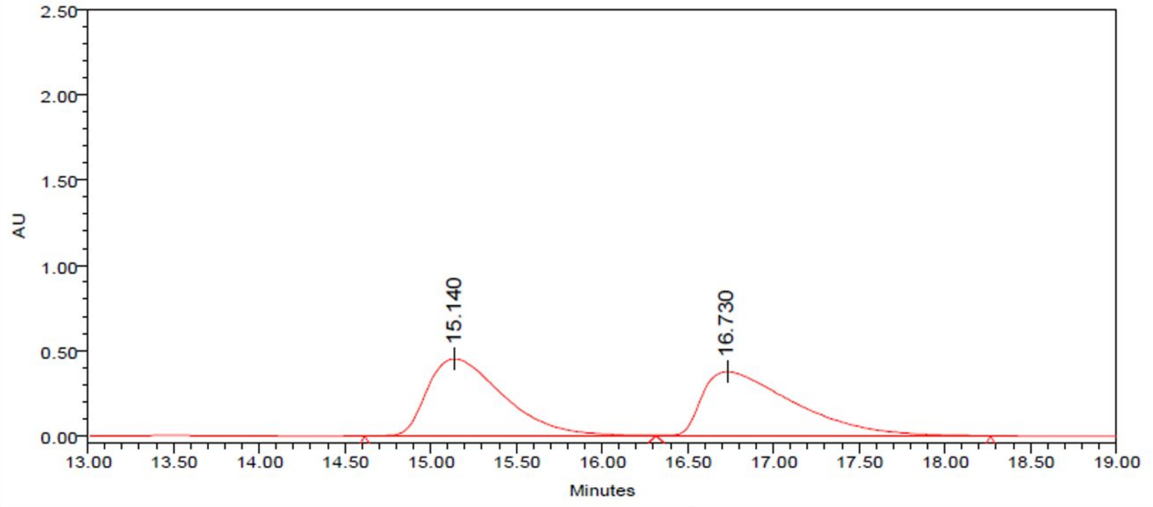

Processed Channel: PDA 241.3 nm

	Processed Channel	Retention Time (min)	Area	% Area	Height
1	PDA 241.3 nm	15.140	13972863	49.97	452510
2	PDA 241.3 nm	16.730	13989717	50.03	375725

Figure S13. Racemic mixture of **4ga**. Daicel ChiralPak IA column (*n*-hexane/ethyl acetate = 90:10, 1 mL min^{-1}, λ = 241.3 nm).

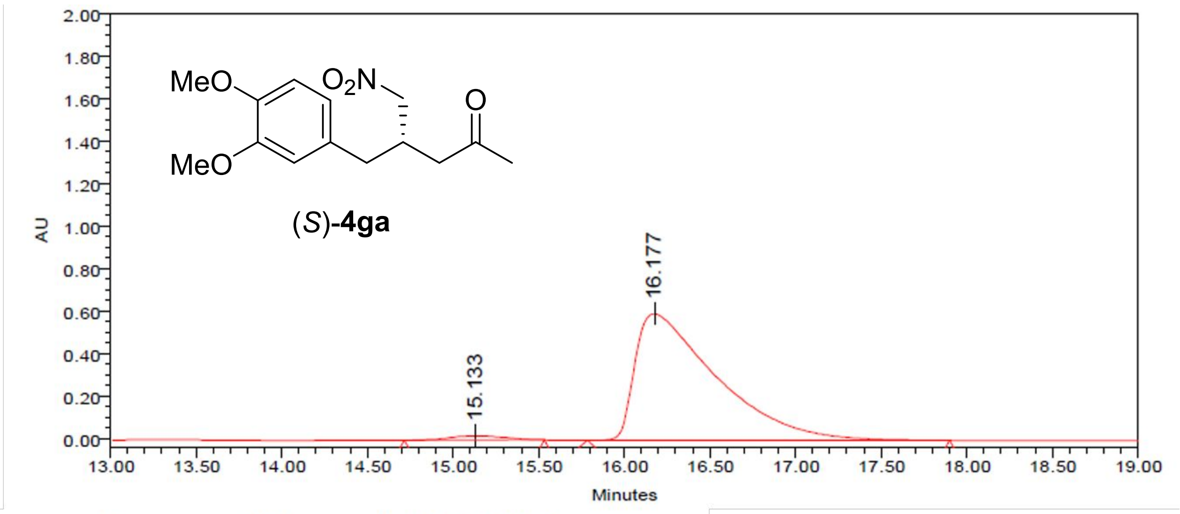

Processed Channel: PDA 241.3 nm

	Processed Channel	Retention Time (min)	Area	% Area	Height
1	PDA 241.3 nm	15.133	431439	2.15	19912
2	PDA 241.3 nm	16.177	19638183	97.85	596262

Figure S14. Enantioenriched mixture of (*S*)-**4ga** (2:98 e.r.). Daicel ChiralPak IA column (*n*-hexane/ethyl acetate = 90:10, 1 mL min^{-1}, λ = 241.3 nm).

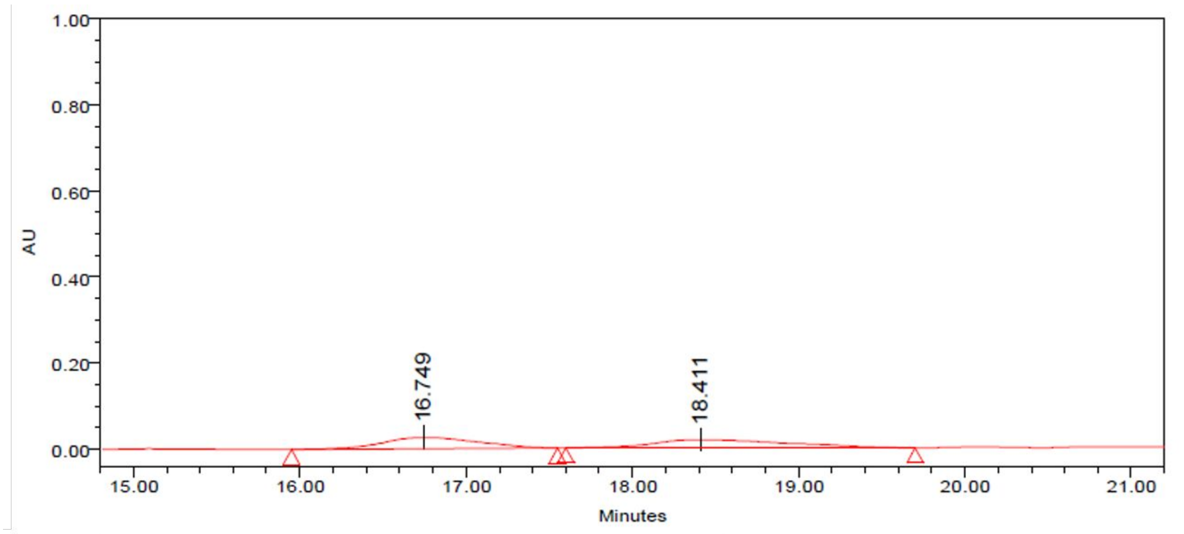

Processed Channel: PDA 250.0 nm

	Processed Channel	Retention Time (min)	Area	% Area	Height
1	PDA 250.0 nm	16.749	1054921	49.09	26212
2	PDA 250.0 nm	18.411	1094153	50.91	19151

Figure S15. Racemic mixture of **4ha**. Daicel ChiralPak IC column (*n*-hexane/ethyl acetate = 90:10, 1 mL min^{-1}, λ = 250.0 nm).

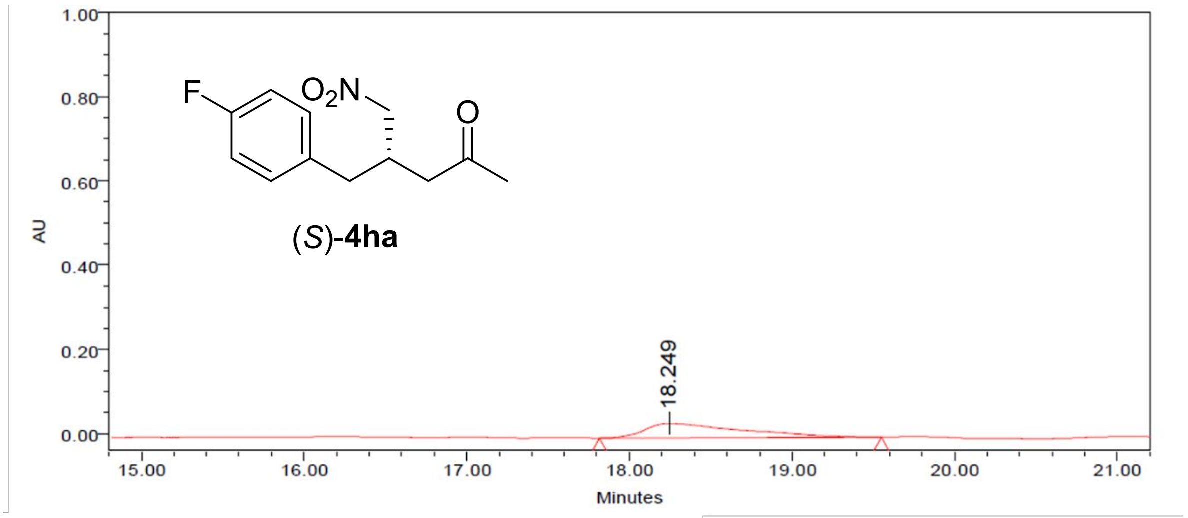

Processed Channel: PDA 250.0 nm

	Processed Channel	Retention Time (min)	Area	% Area	Height
1	PDA 250.0 nm	18.249	1395065	100.00	33629

Figure S16. Enantioenriched mixture of (*S*)-**4ha** (>99:1 e.r.). Daicel ChiralPak IC column (*n*-hexane/ethyl acetate = 90:10, 1 mL min^{-1}, λ = 250.0 nm).

13. Spectroscopic data of studied compounds

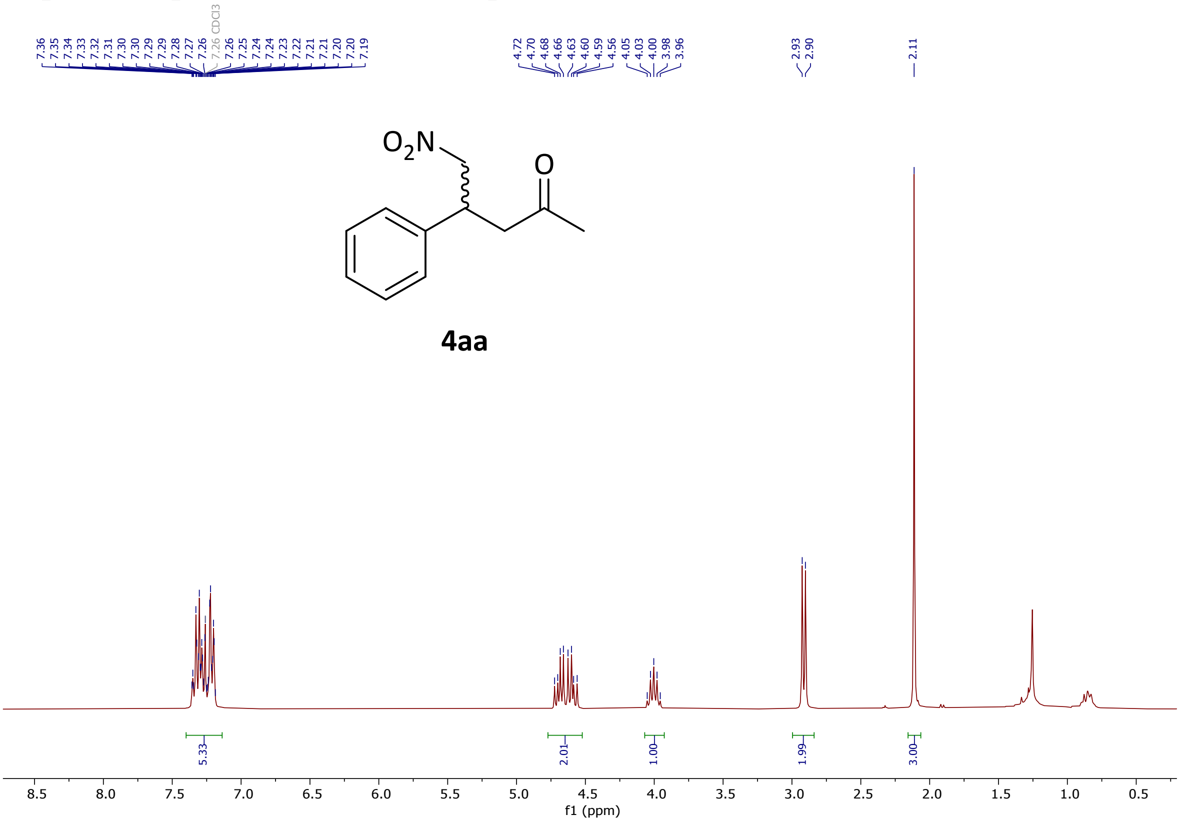

Figure S17. ^{1}H NMR spectrum of 5-nitro-4-phenylpentan-2-one (**4aa**). (300 Hz, $CDCl_3$).

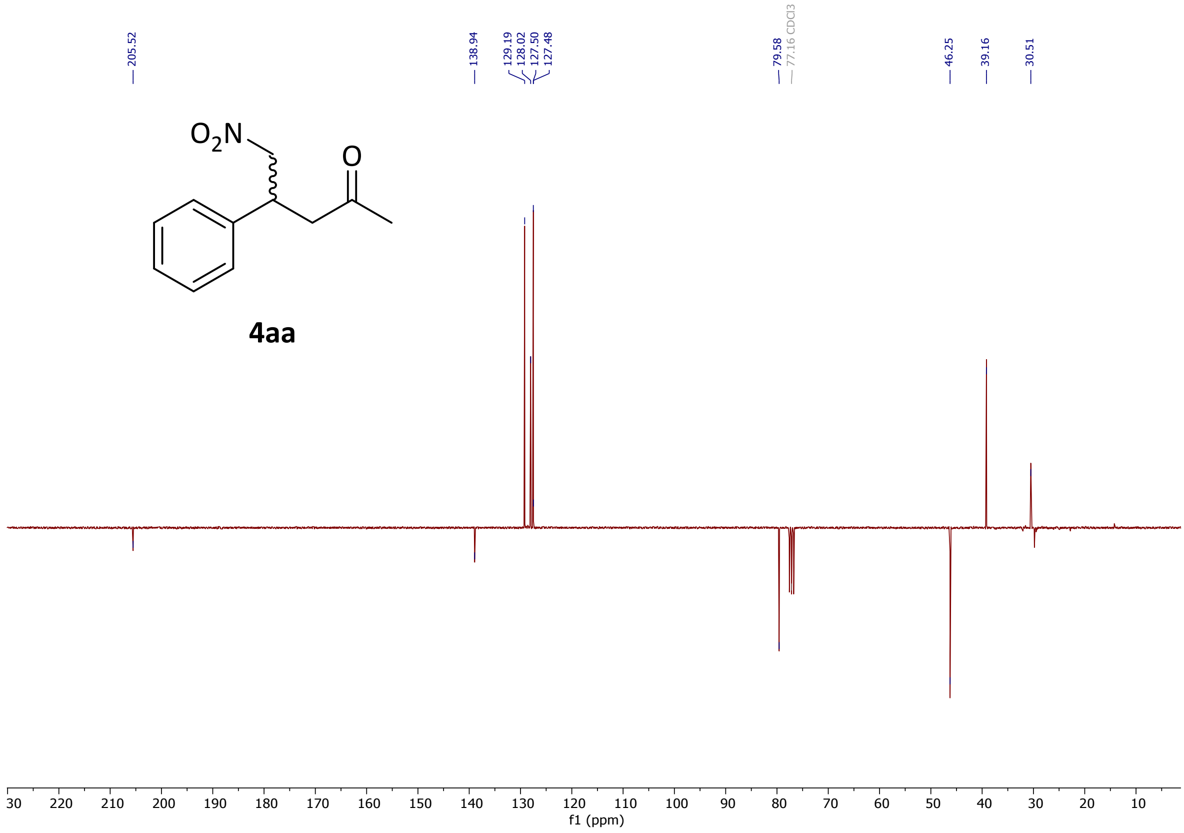

Figure S18. ^{13}C{^{1}H}-APT NMR spectrum of 5-nitro-4-phenylpentan-2-one (**4aa**). (300 Hz, $CDCl_3$).

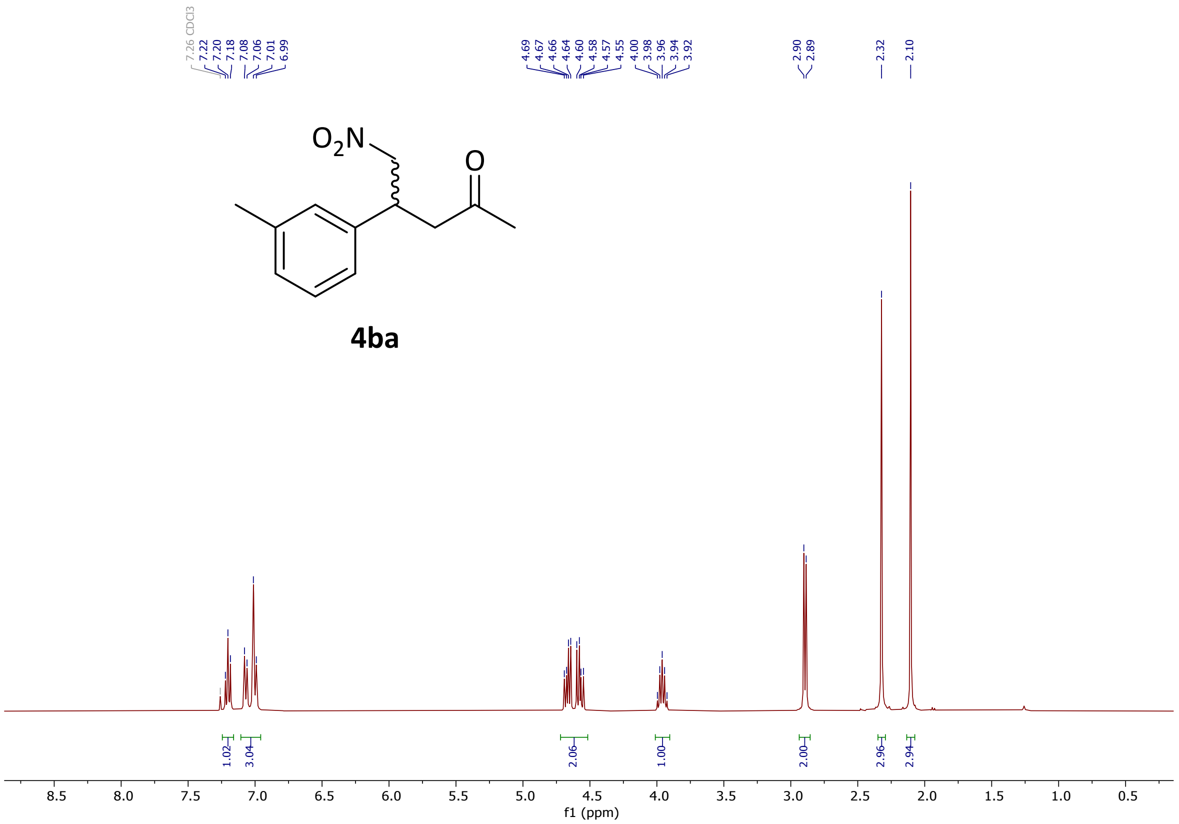

Figure S19. ^{1}H NMR spectrum of 5-nitro-4-(m-tolyl)pentan-2-one (**4ba**). (400 Hz, $CDCl_3$).

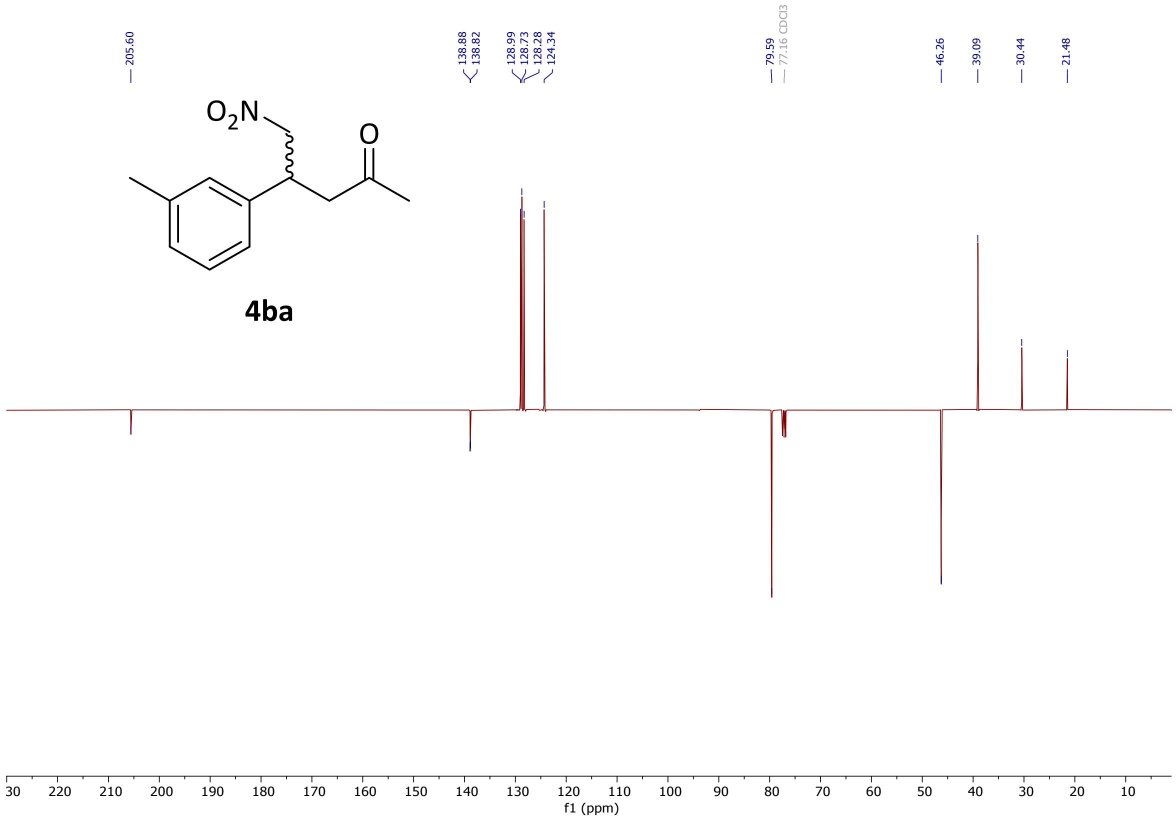

Figure S20. $^{13}C\{^{1}H\}$-APT NMR spectrum of 5-nitro-4-(*m*-tolyl)pentan-2-one (**4ba**). (101 Hz, $CDCl_3$).

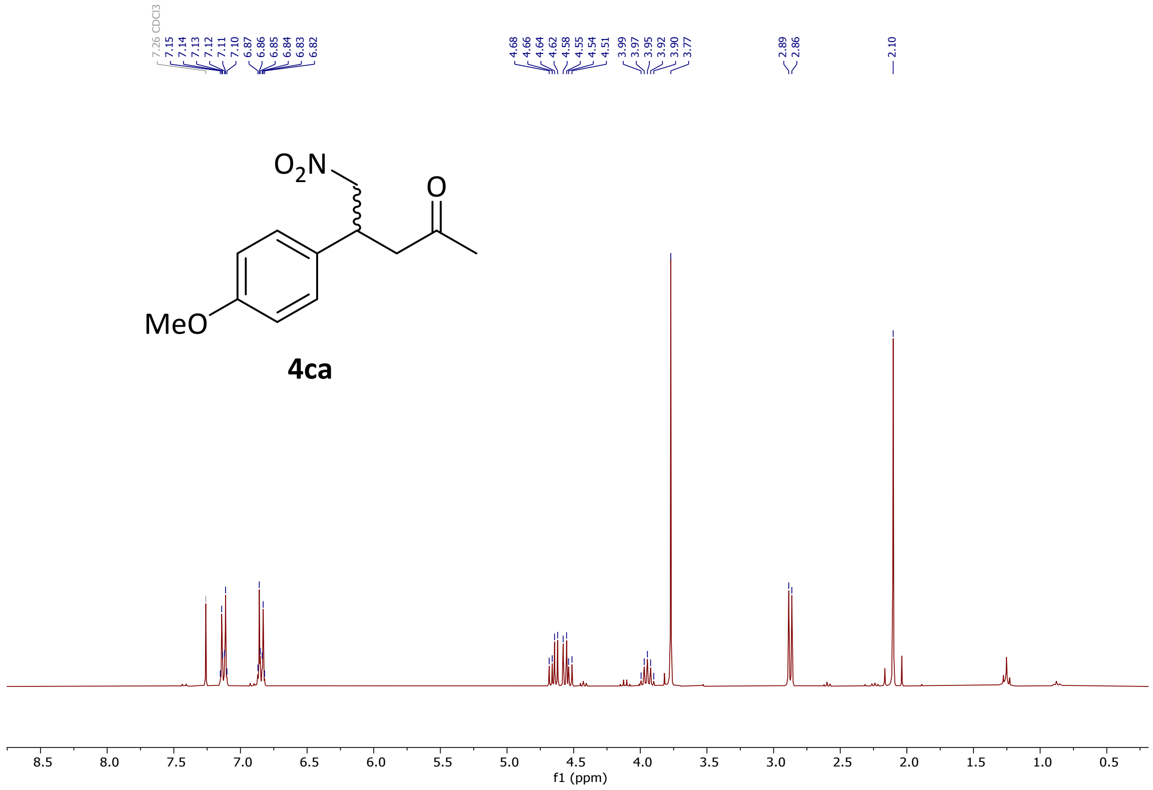

Figure S21. ^{1}H NMR spectrum of 4-(4-methoxyphenyl)-5-nitropentan-2-one (**4ca**). (300 Hz, $CDCl_3$).

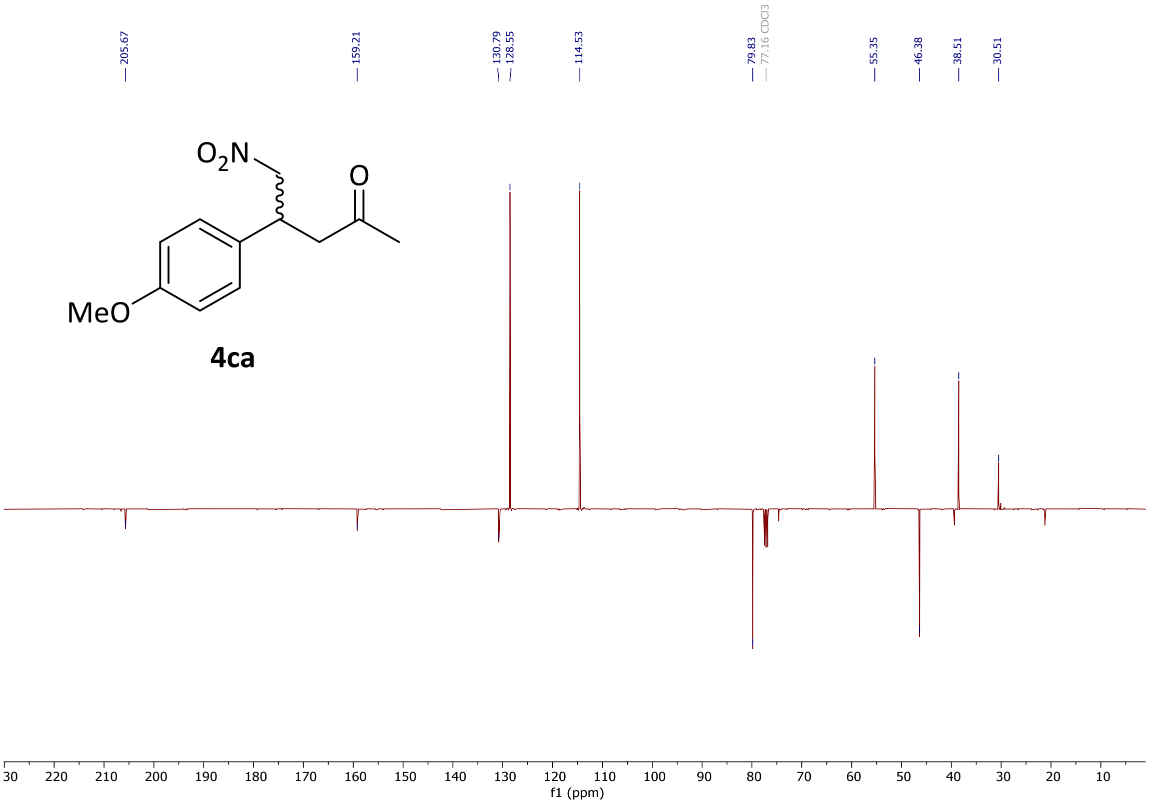

Figure S22. $^{13}C\{^{1}H\}$-APT NMR spectrum of 4-(4-methoxyphenyl)-5-nitropentan-2-one (**4ca**). (75 Hz, $CDCl_3$).

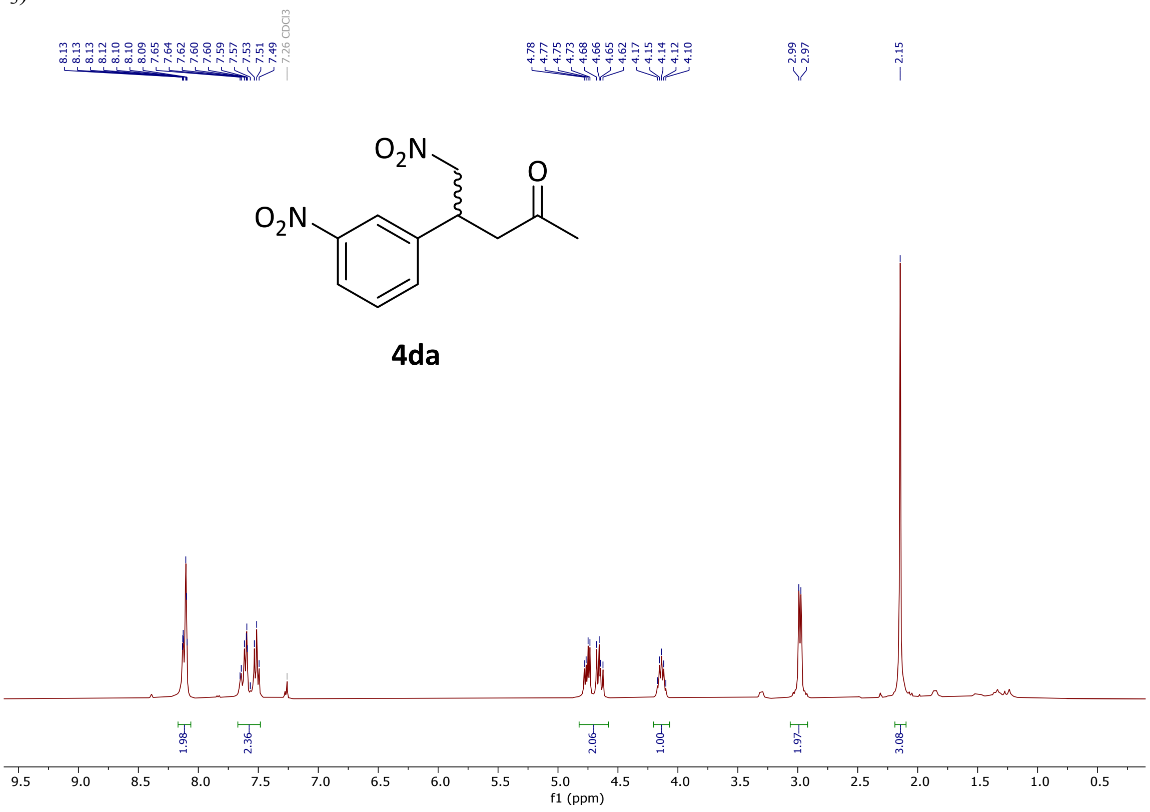

Figure S23. ^{1}H NMR spectrum of 5-nitro-4-(3-nitrophenyl)pentan-2-one (**4da**). (400 Hz, $CDCl_3$).

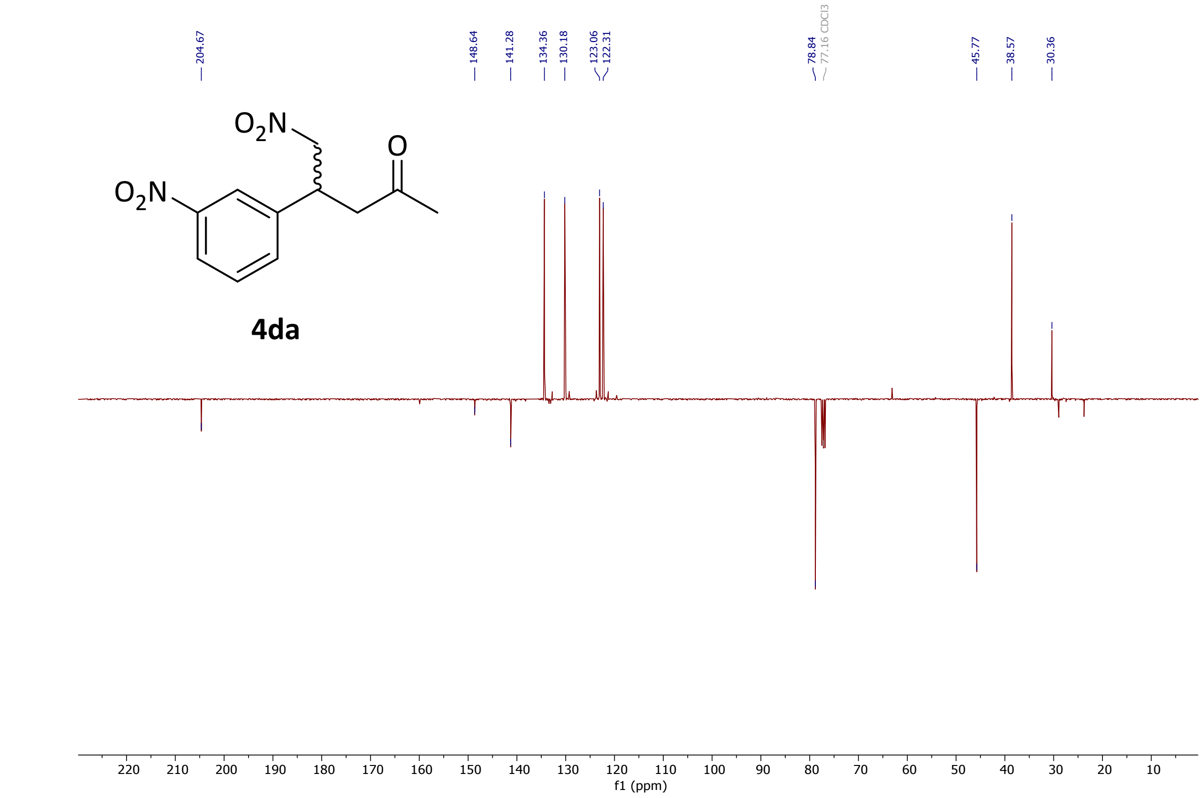

Figure S24. $^{13}C\{^{1}H\}$-APT NMR spectrum of 5-nitro-4-(3-nitrophenyl)pentan-2-one (**4da**). (101 Hz, $CDCl_3$).

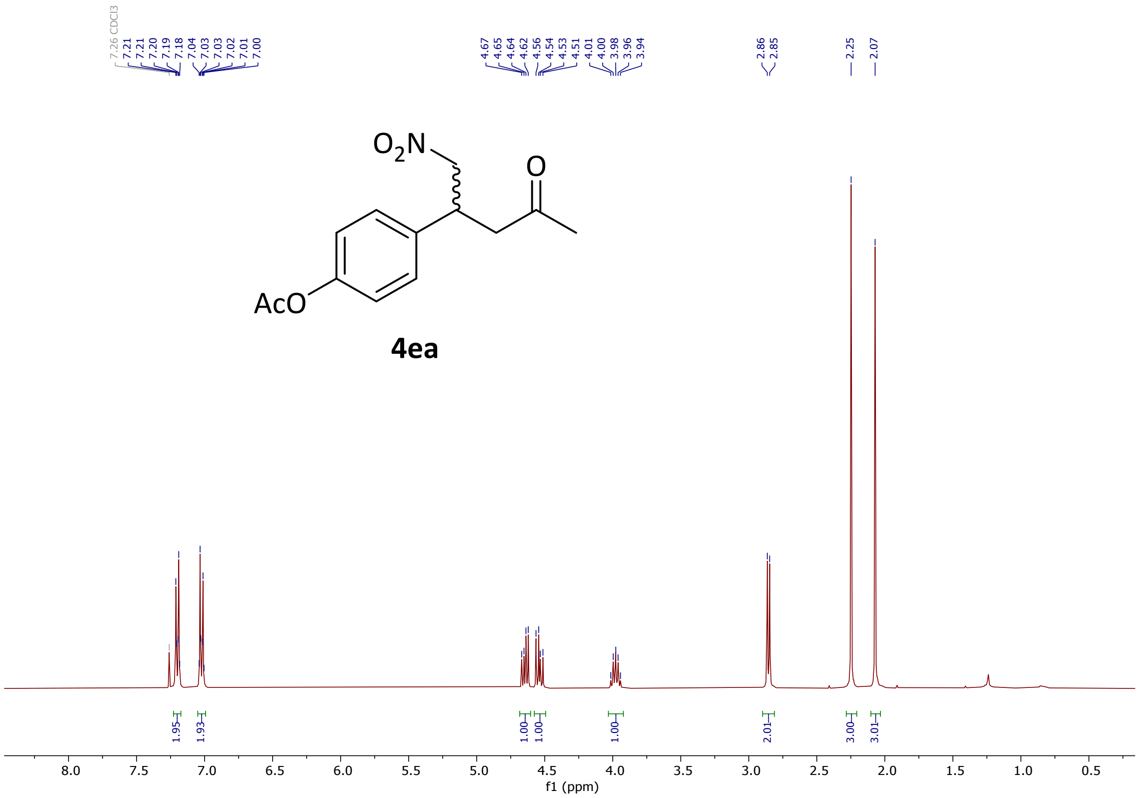

Figure S25. ^{1}H NMR spectrum of 4-(1-nitro-4-oxopentan-2-yl)phenyl acetate (**4ea**). (400 Hz, $CDCl_3$).

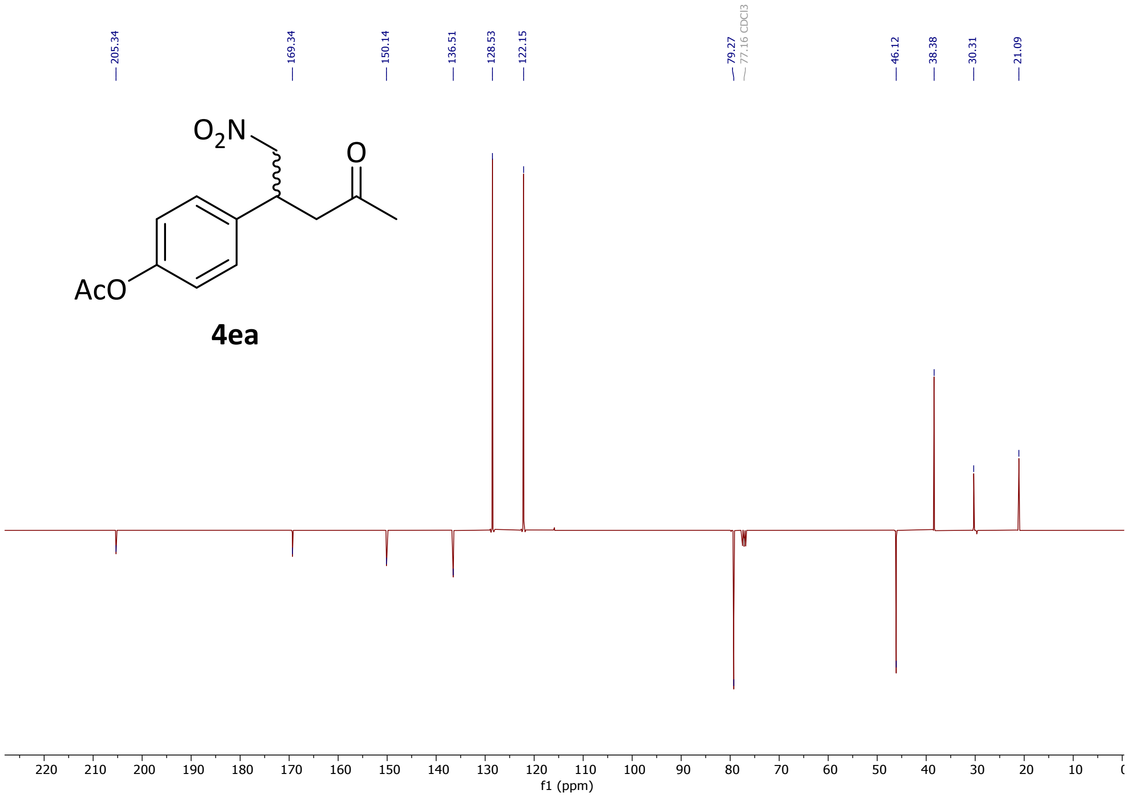

Figure S26. $^{13}C\{^{1}H\}$-APT NMR spectrum of 4-(1-nitro-4-oxopentan-2-yl)phenyl acetate (**4ea**). (101 Hz, $CDCl_3$).

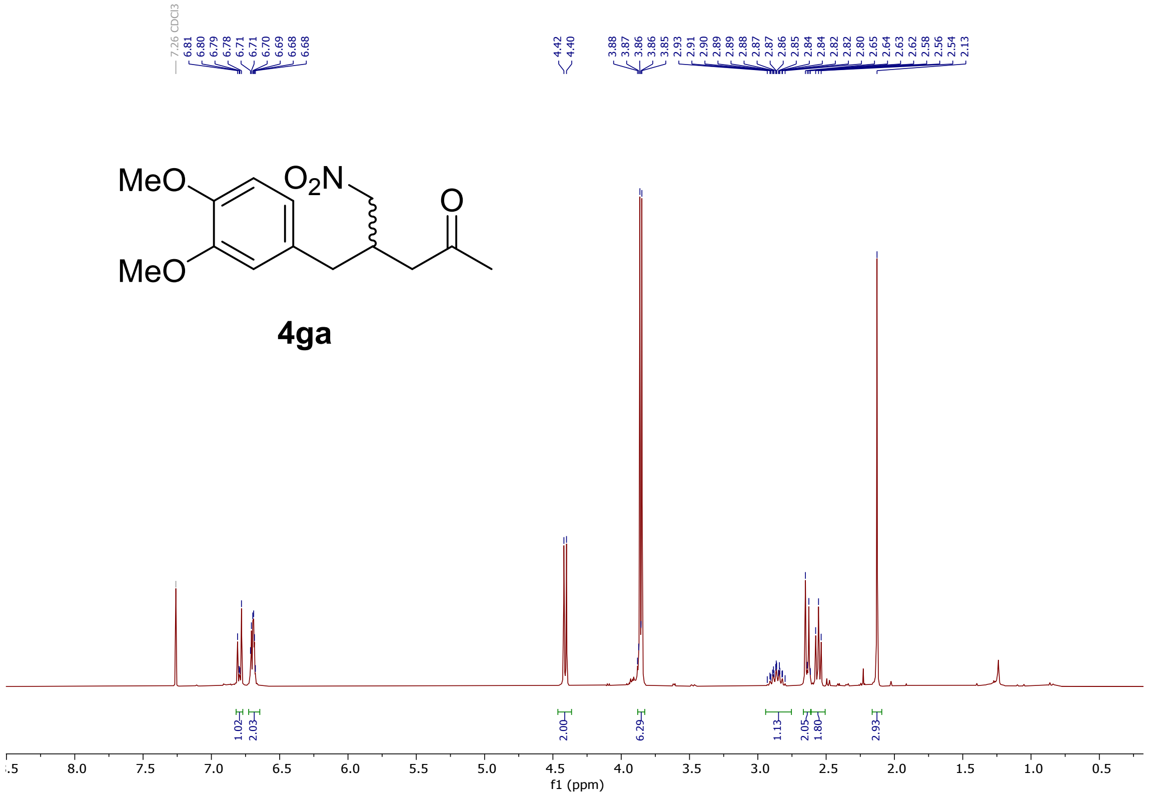

Figure S27. ^{1}H NMR spectrum of 4-(3,4-dimethoxybenzyl)-5-nitropentan-2-one (**4ga**). (300 Hz, $CDCl_3$).

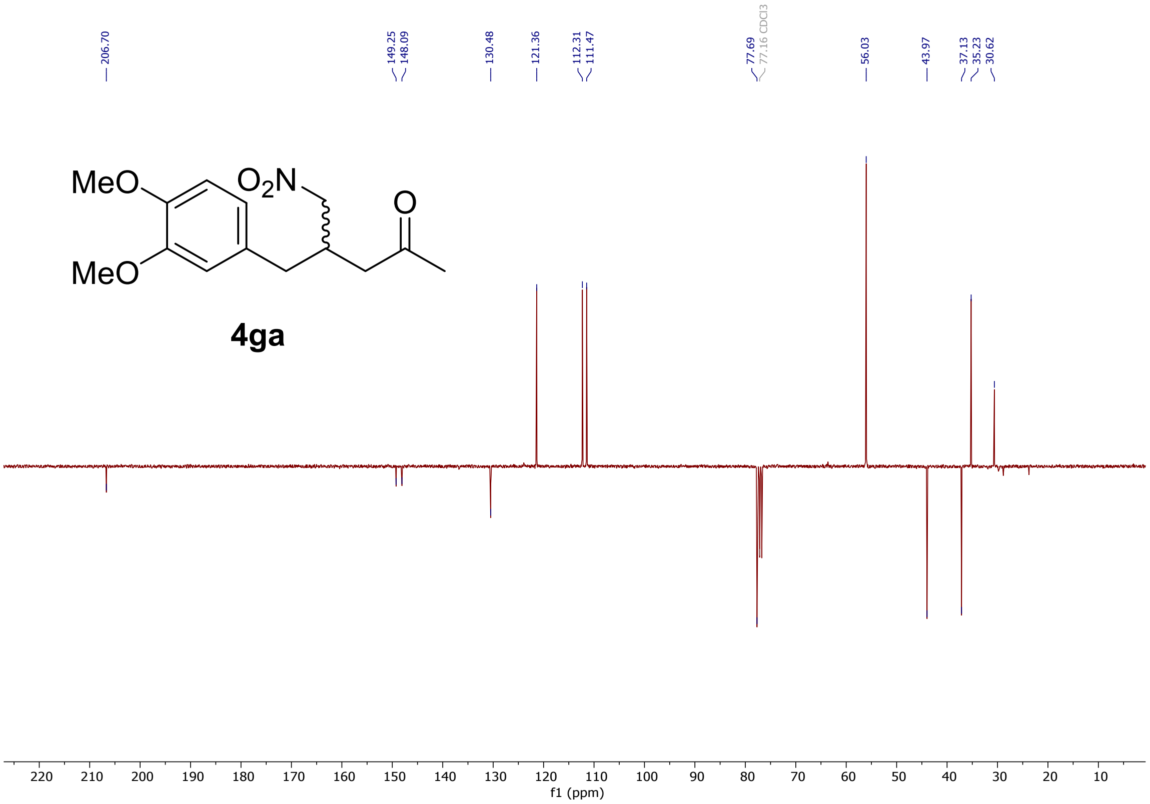

Figure S28. $^{13}C\{^{1}H\}$-APT NMR spectrum of 4-(3,4-dimethoxybenzyl)-5-nitropentan-2-one (**4ga**). (75 Hz, $CDCl_3$).

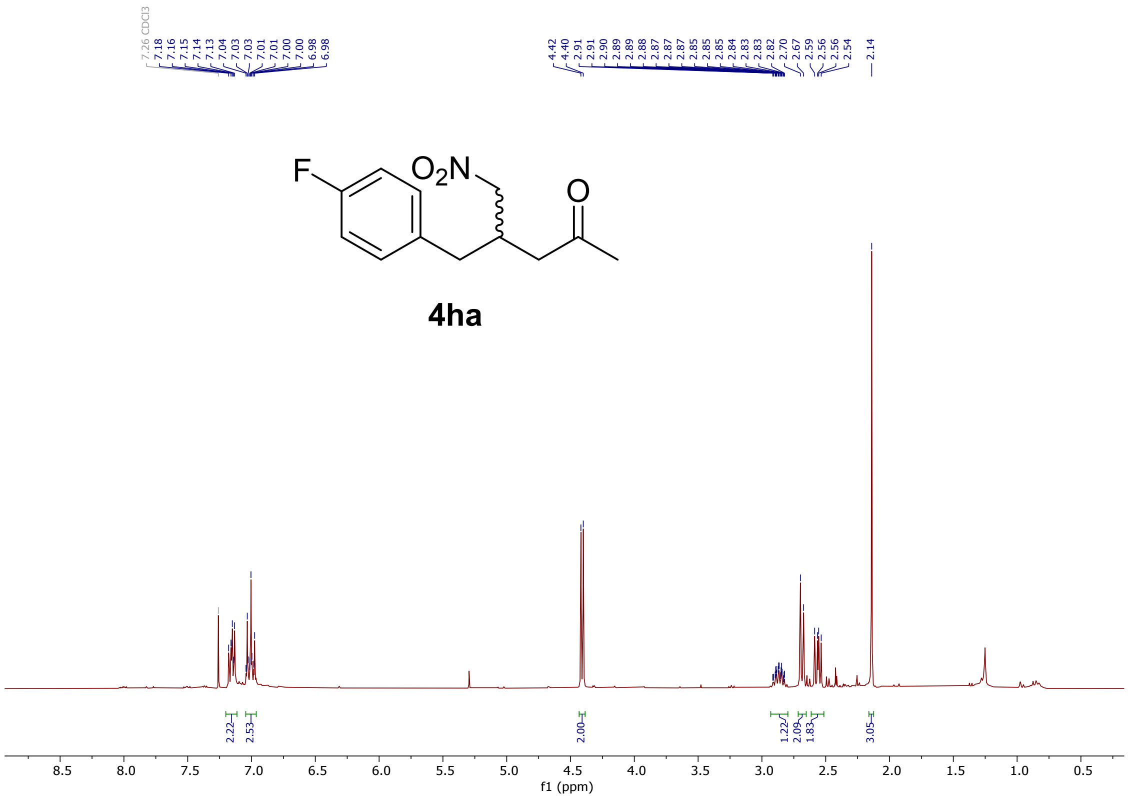

Figure S29. ^{1}H NMR spectrum of 4-(4-fluorobenzyl)-5-nitropentan-2-one (**4ha**). (300 Hz, $CDCl_3$).

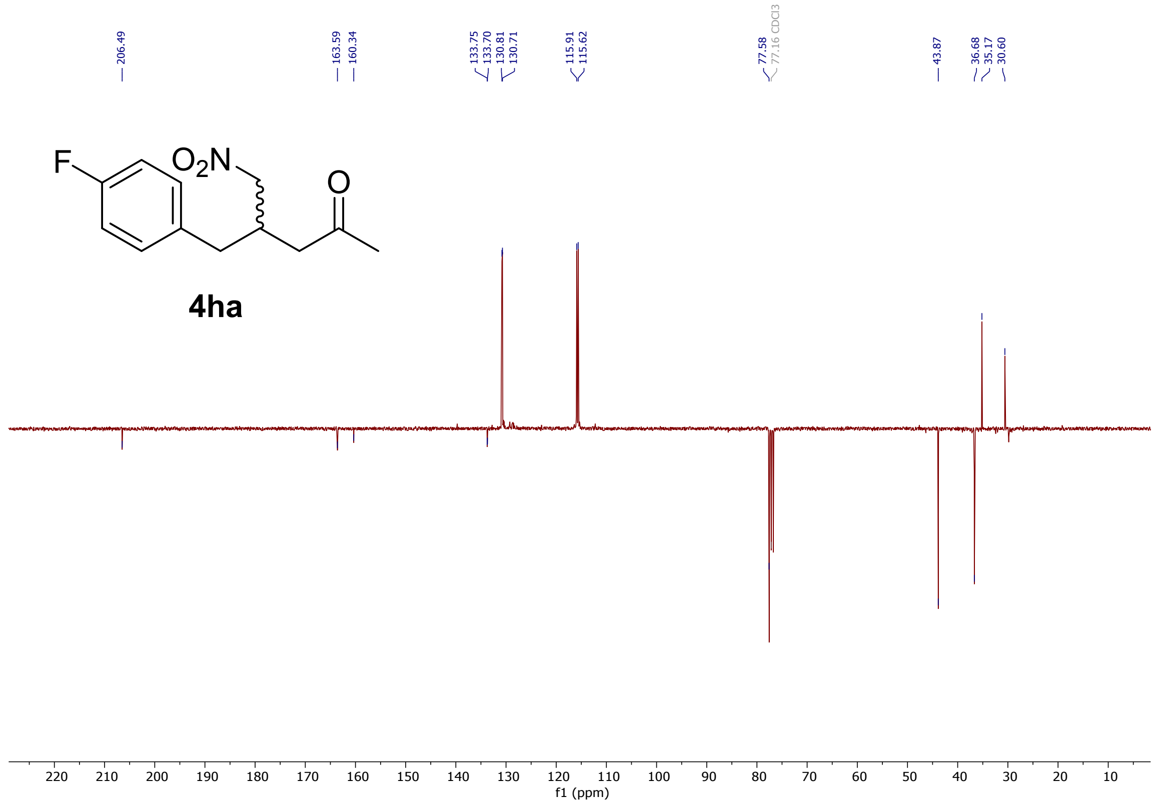

Figure S30. $^{13}C\{^{1}H\}$-APT NMR spectrum of 4-(4-fluorobenzyl)-5-nitropentan-2-one (**4ha**). (75 Hz, $CDCl_3$).

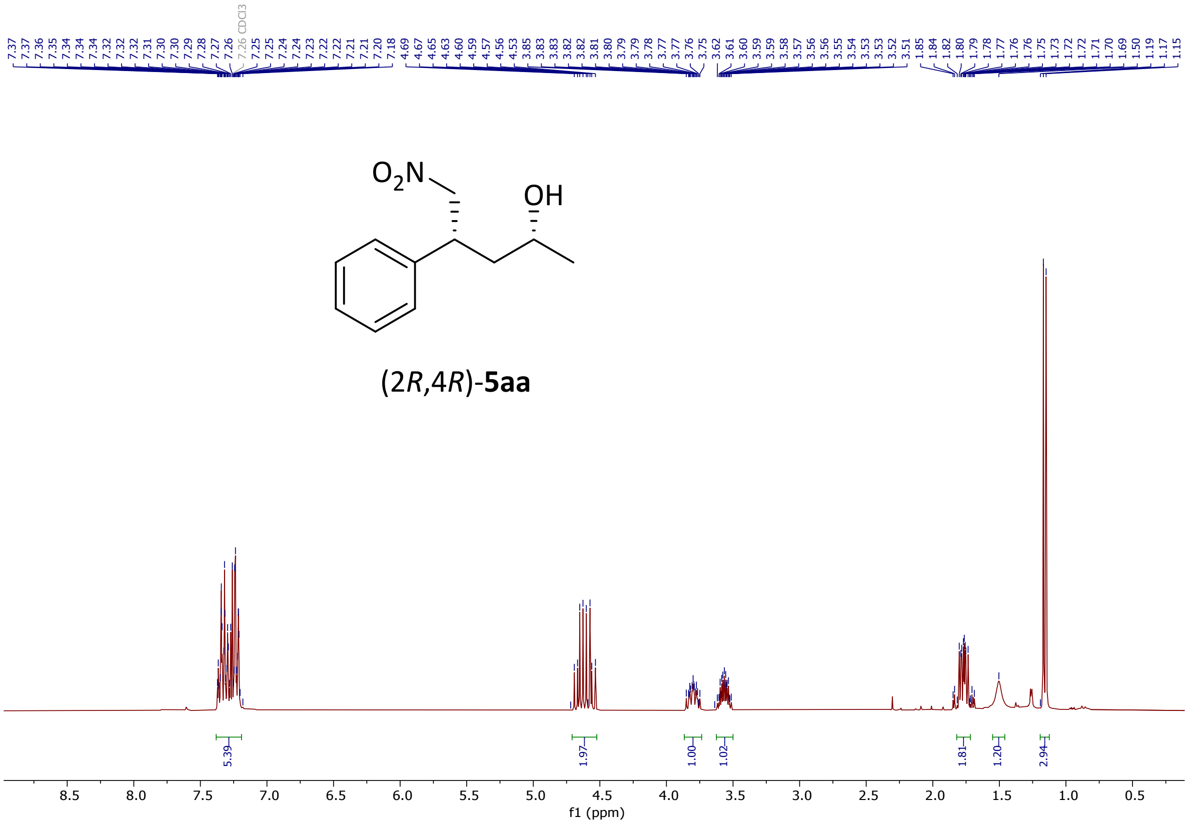

Figure S31. ^{1}H NMR spectrum of (2*S*,4*R*)-5-nitro-4-phenylpentan-2-ol (**5aa**). (300 Hz, $CDCl_3$).

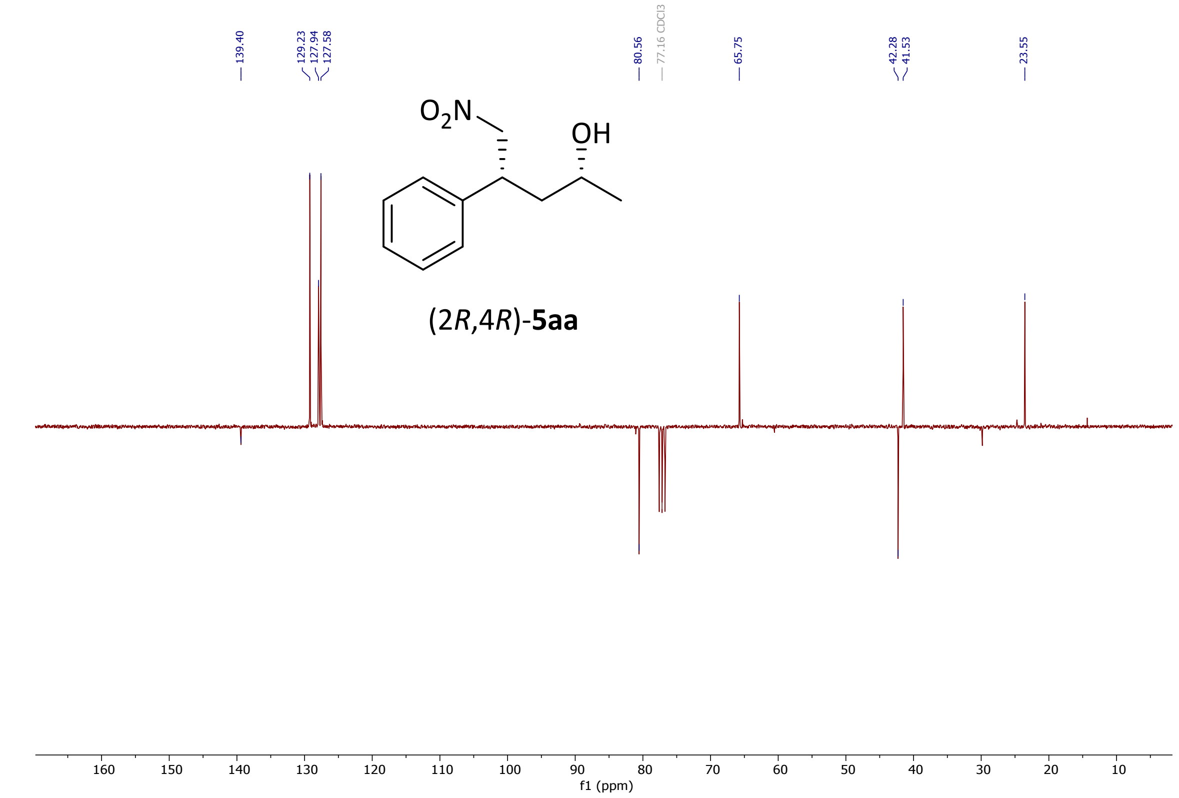

Figure S32. $^{13}C\{^{1}H\}$-APT NMR spectrum of (2*S*,4*R*)-5-nitro-4-phenylpentan-2-ol (**5aa**). (75 Hz, $CDCl_3$).

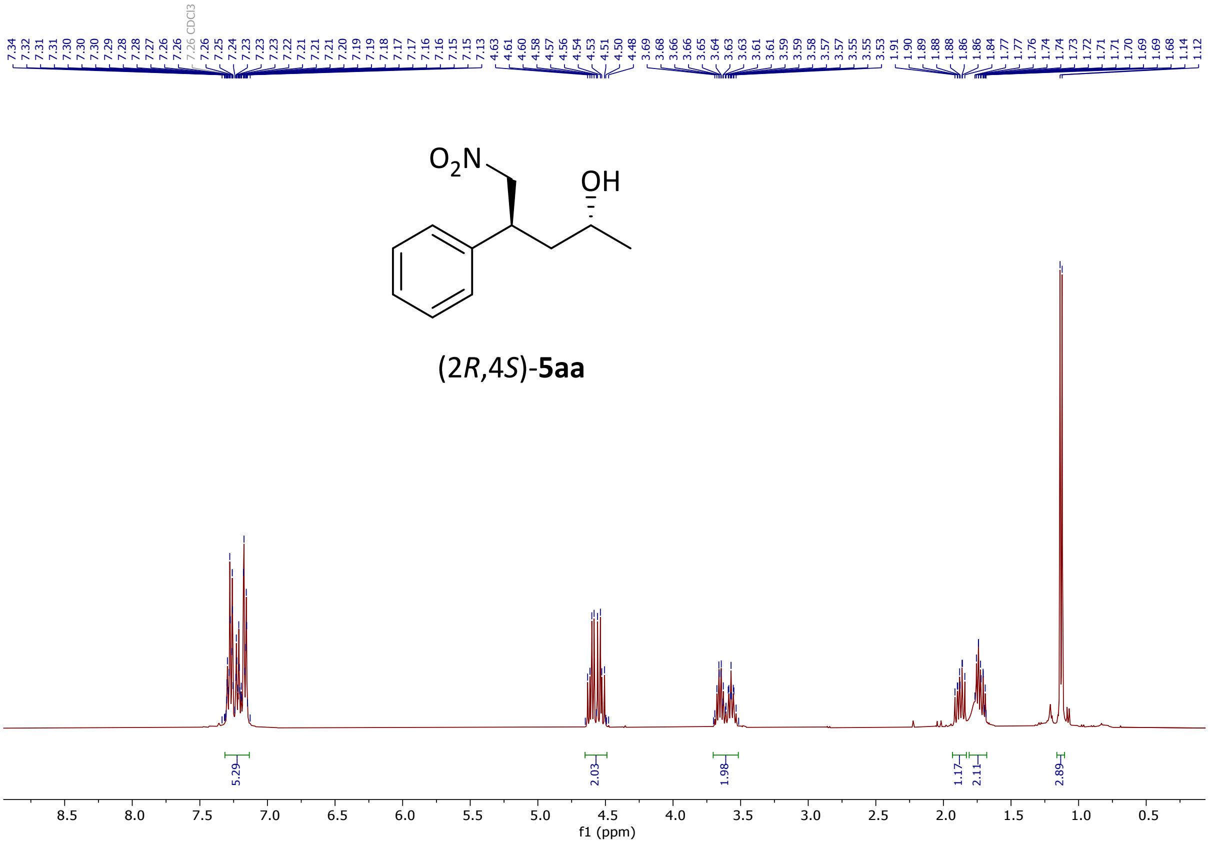

Figure S33. ^{1}H NMR spectrum of (2*R*,4*S*)-5-nitro-4-phenylpentan-2-ol (**5aa**). (300 Hz, $CDCl_3$).

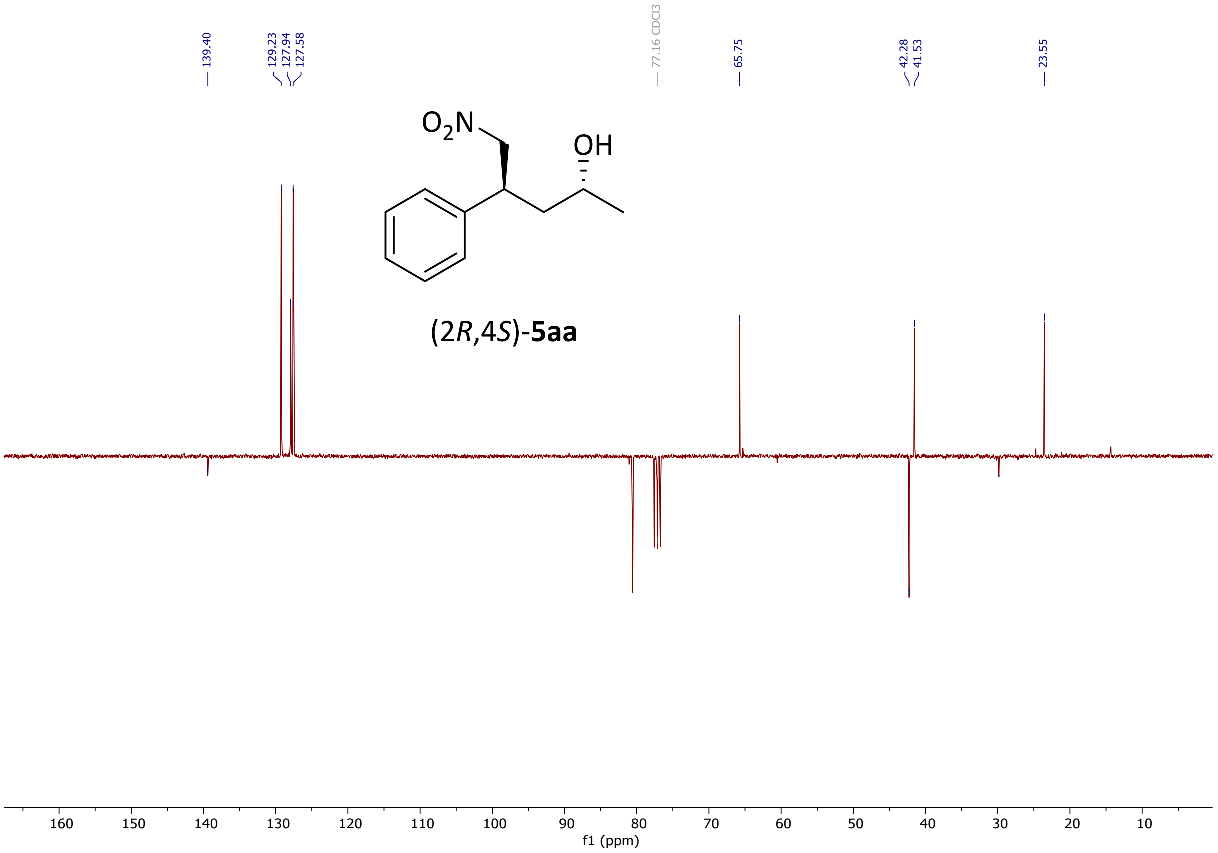

Figure S34. $^{13}C\{^{1}H\}$-APT NMR spectrum of (2*R*,4*S*)-5-nitro-4-phenylpentan-2-ol (**5aa**). (75 Hz, $CDCl_3$).

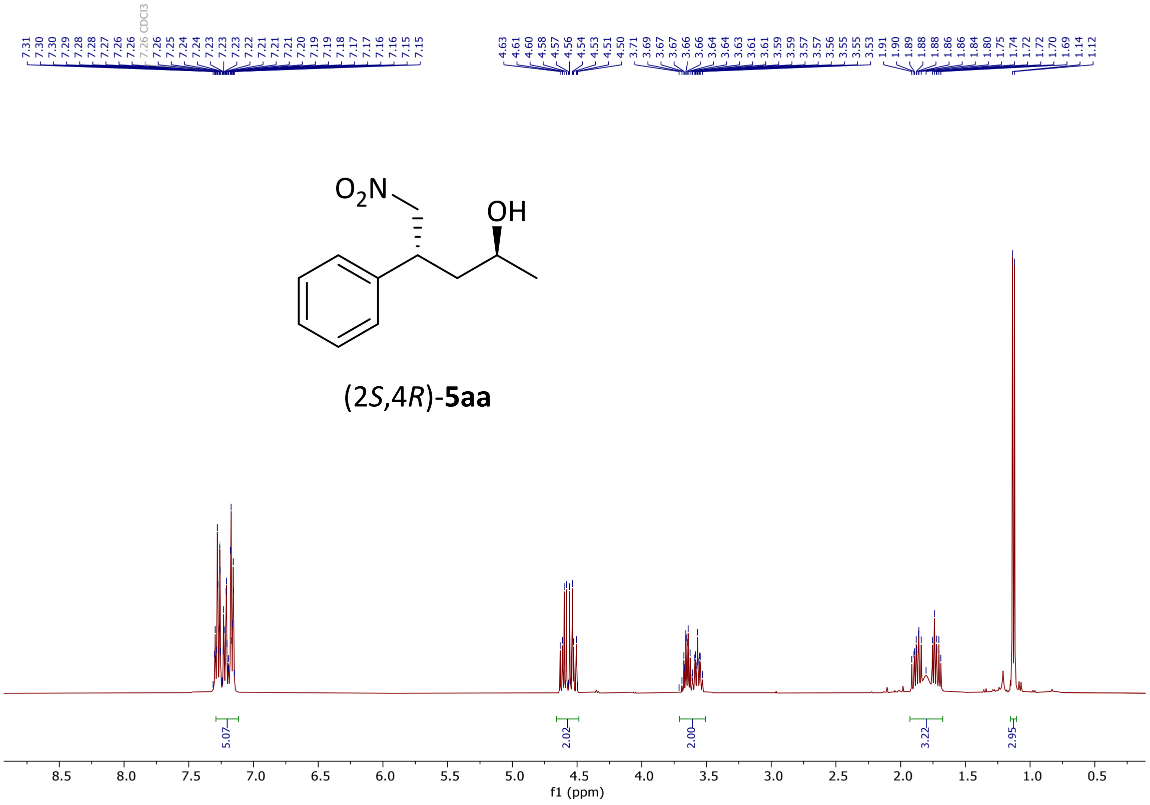

Figure S35. ^{1}H NMR spectrum of (2*S*,4*R*)-5-nitro-4-phenylpentan-2-ol (**5aa**). (300 Hz, $CDCl_3$).

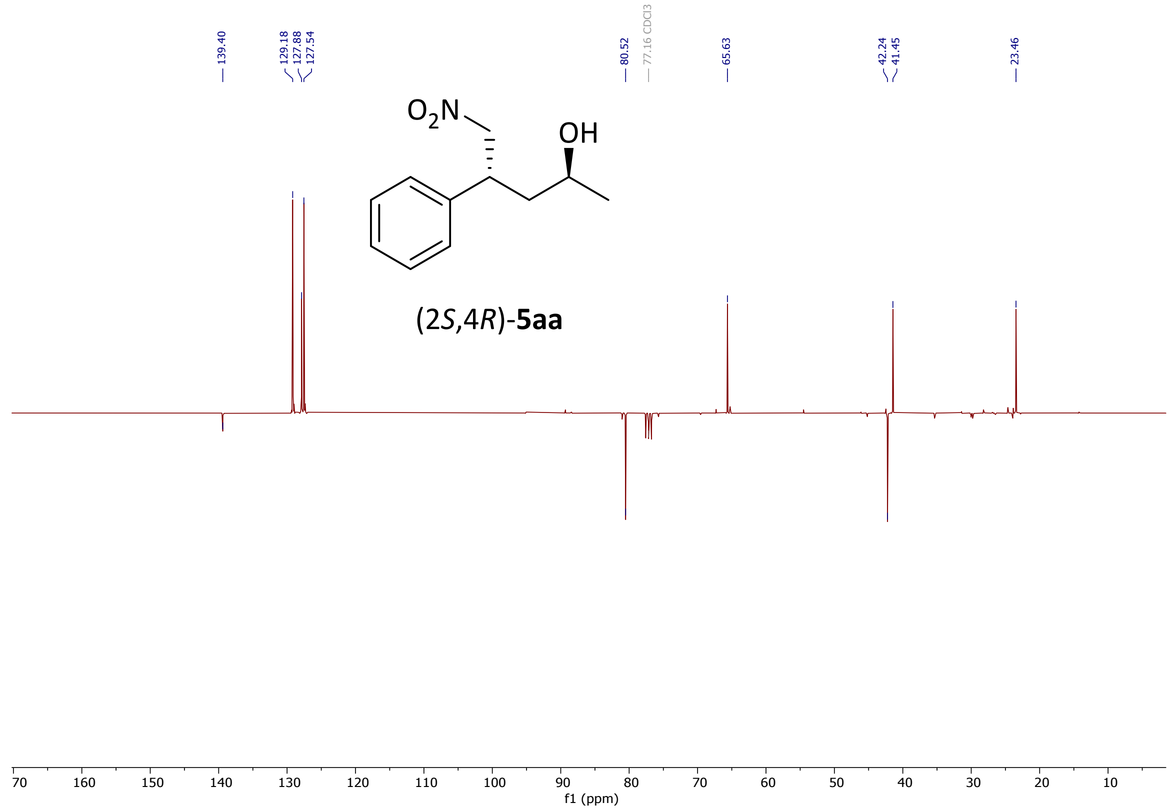

Figure S36. $^{13}C\{^{1}H\}$-APT NMR spectrum of (2*S*,4*R*)-5-nitro-4-phenylpentan-2-ol (**5aa**). (75 Hz, $CDCl_3$).

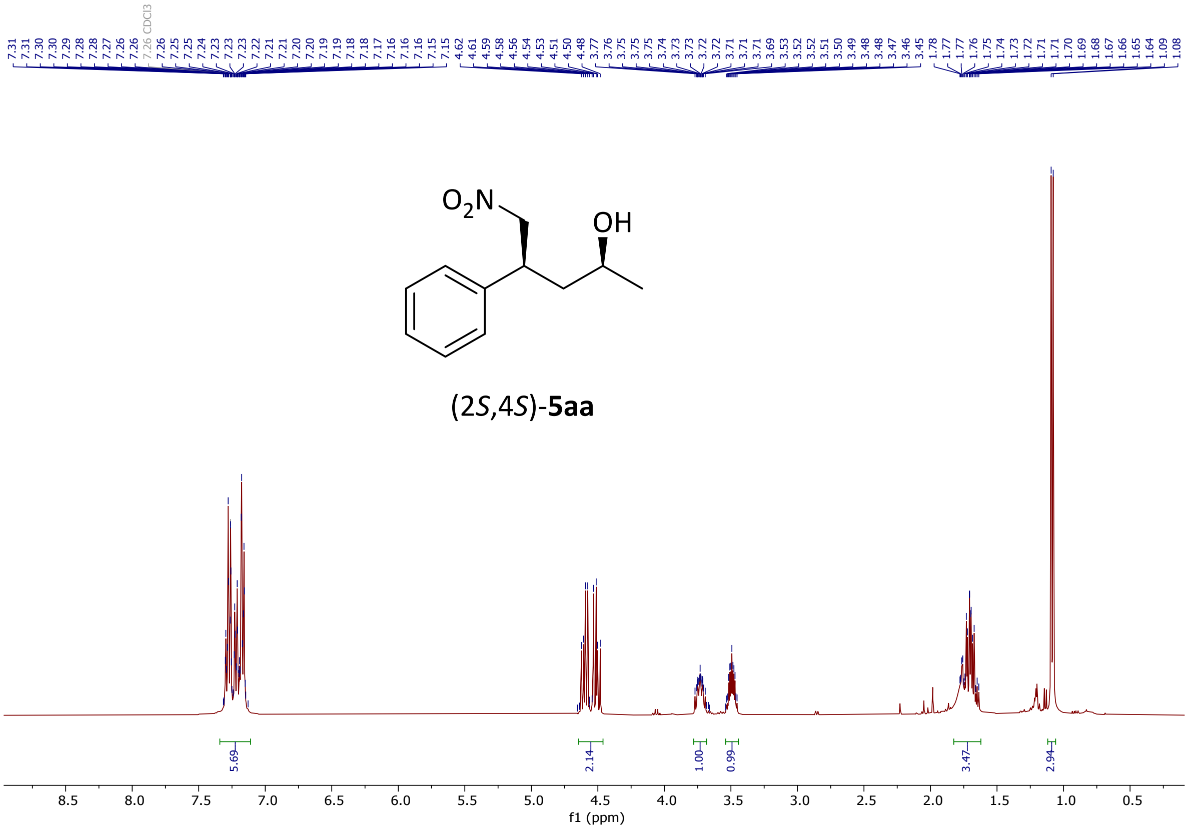

Figure S37. ^{1}H NMR spectrum of (2*S*,4*S*)-5-nitro-4-phenylpentan-2-ol (**5aa**). (400 Hz, $CDCl_3$).

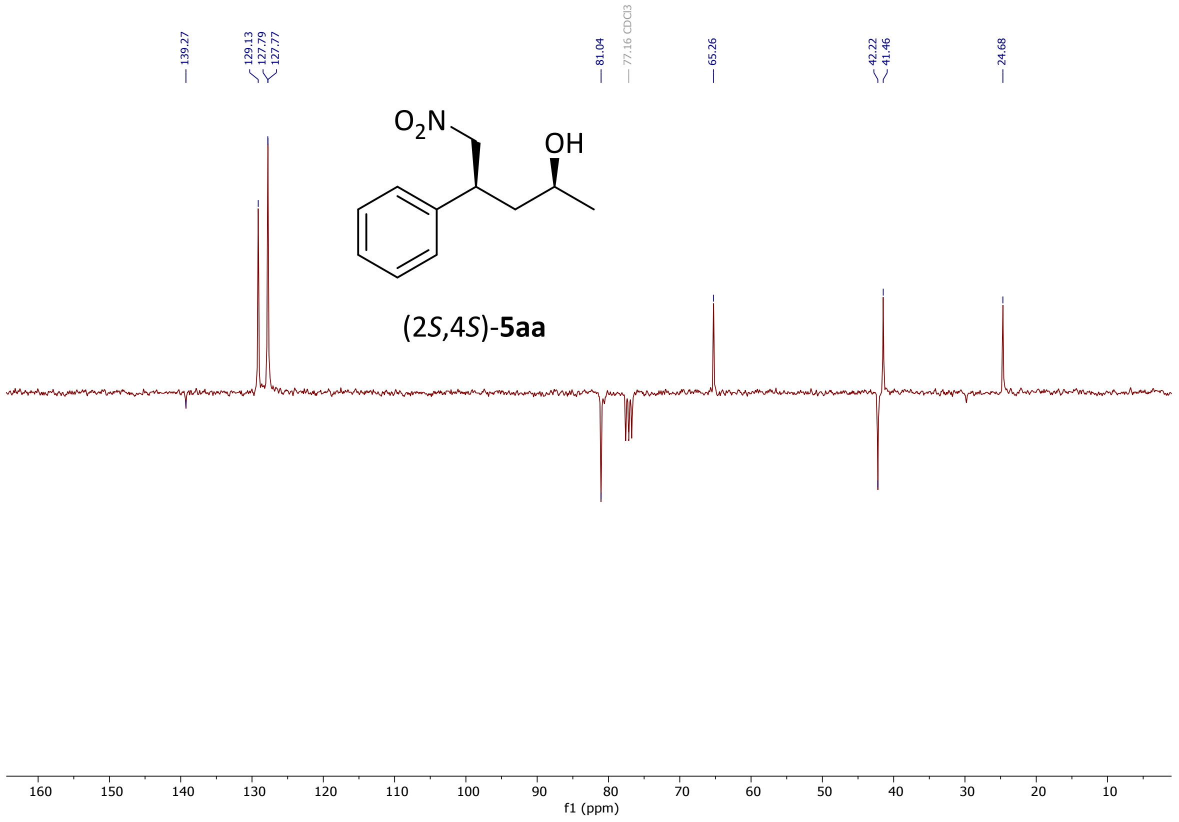

Figure S38. ${}^{13}C\{{}^{1}H\}$-APT NMR spectrum of (2*S*,4*S*)-5-nitro-4-phenylpentan-2-ol (**5aa**). (101 Hz, $CDCl_3$).

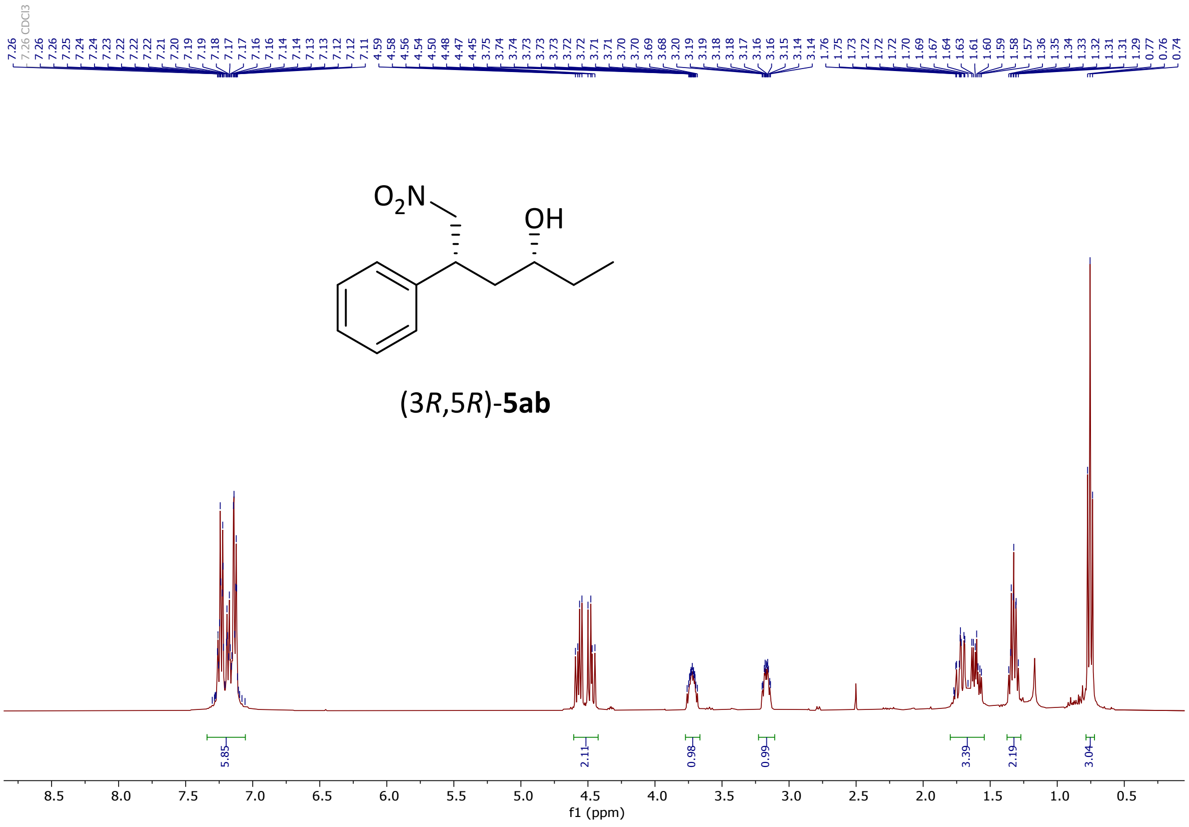

Figure S39. ${}^{1}H$ NMR spectrum of (3*R*,5*R*)-6-nitro-phenylhexan-3-ol (**5ab**). (400 Hz, $CDCl_3$).

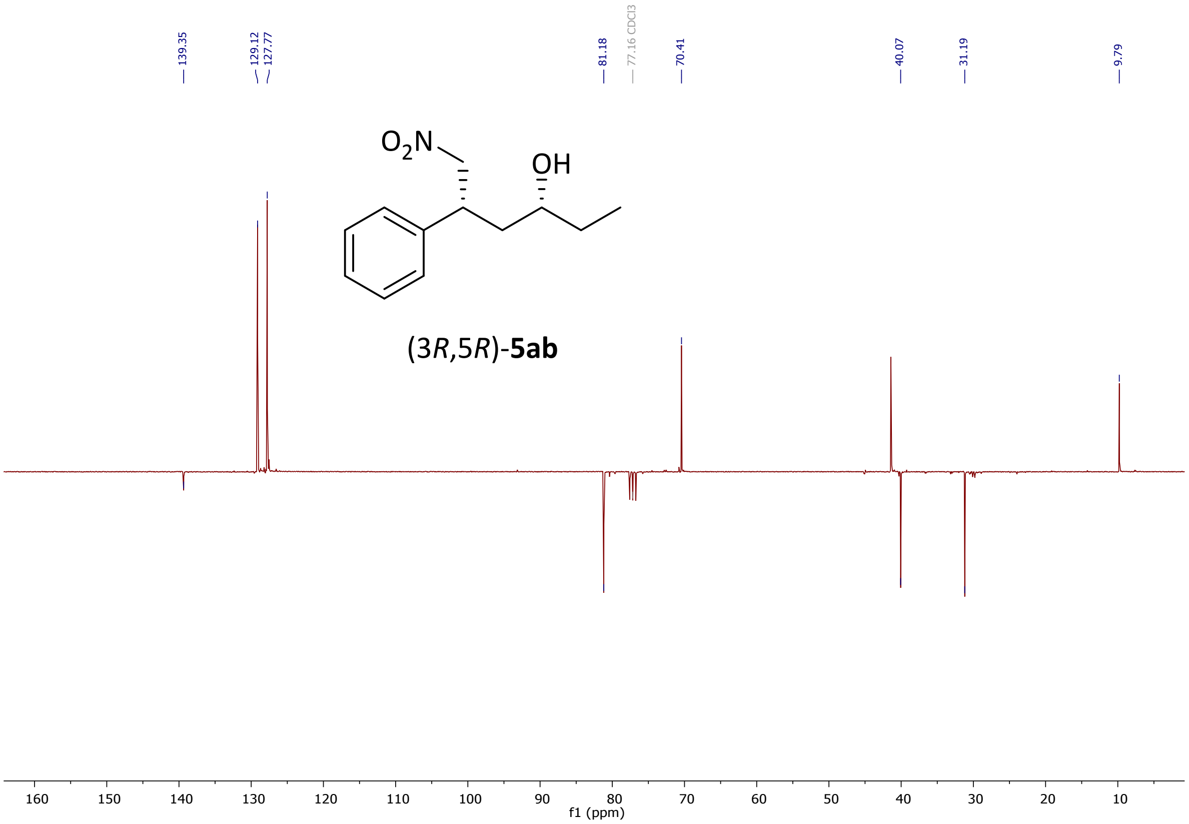

Figure S40. $^{13}C\{^{1}H\}$-APT NMR spectrum of (3*R*,5*R*)-6-nitro-phenylhexan-3-ol (**5ab**). (101 Hz, $CDCl_3$).

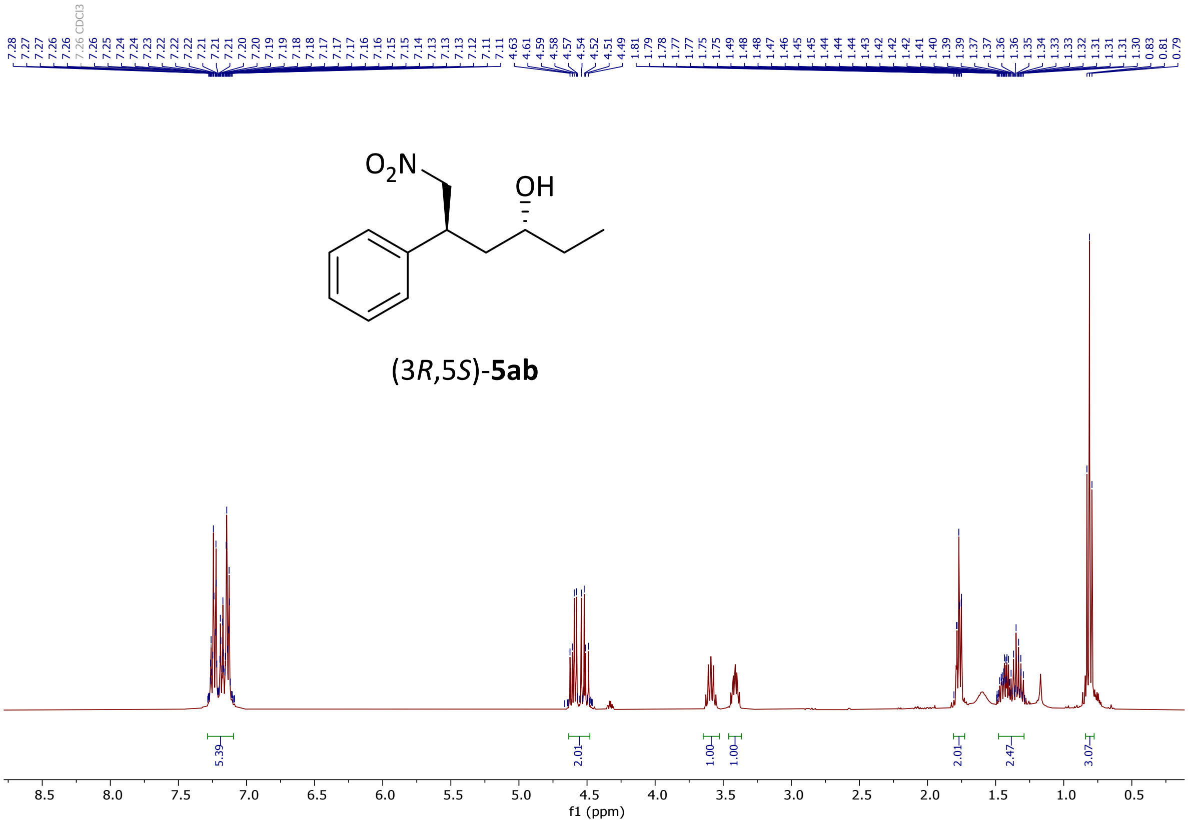

Figure S41. ^{1}H NMR spectrum of (3*R*,5*S*)-6-nitro-phenylhexan-3-ol (**5ab**). (400 Hz, $CDCl_3$).

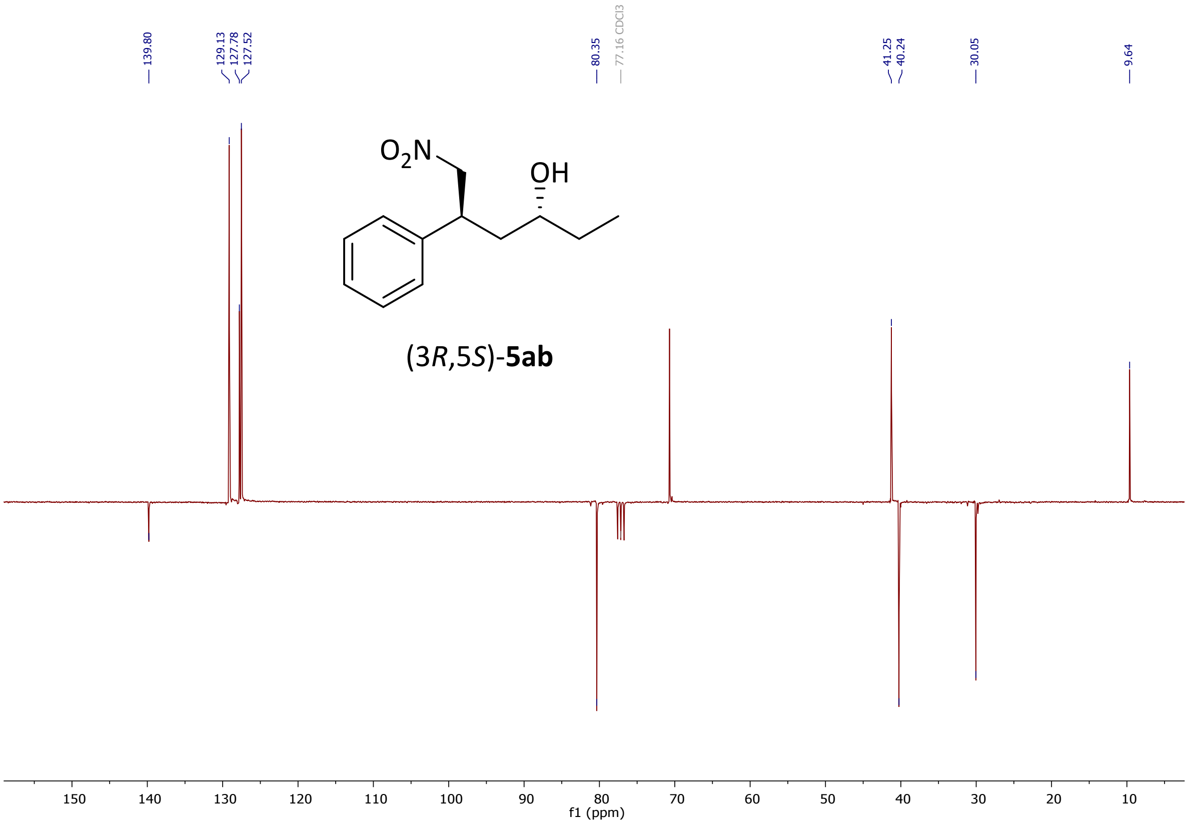

Figure S42. $^{13}C\{^1H\}$-APT NMR spectrum of (3*R*,5*S*)-6-nitro-phenylhexan-3-ol (**5ab**). (101 Hz, $CDCl_3$).

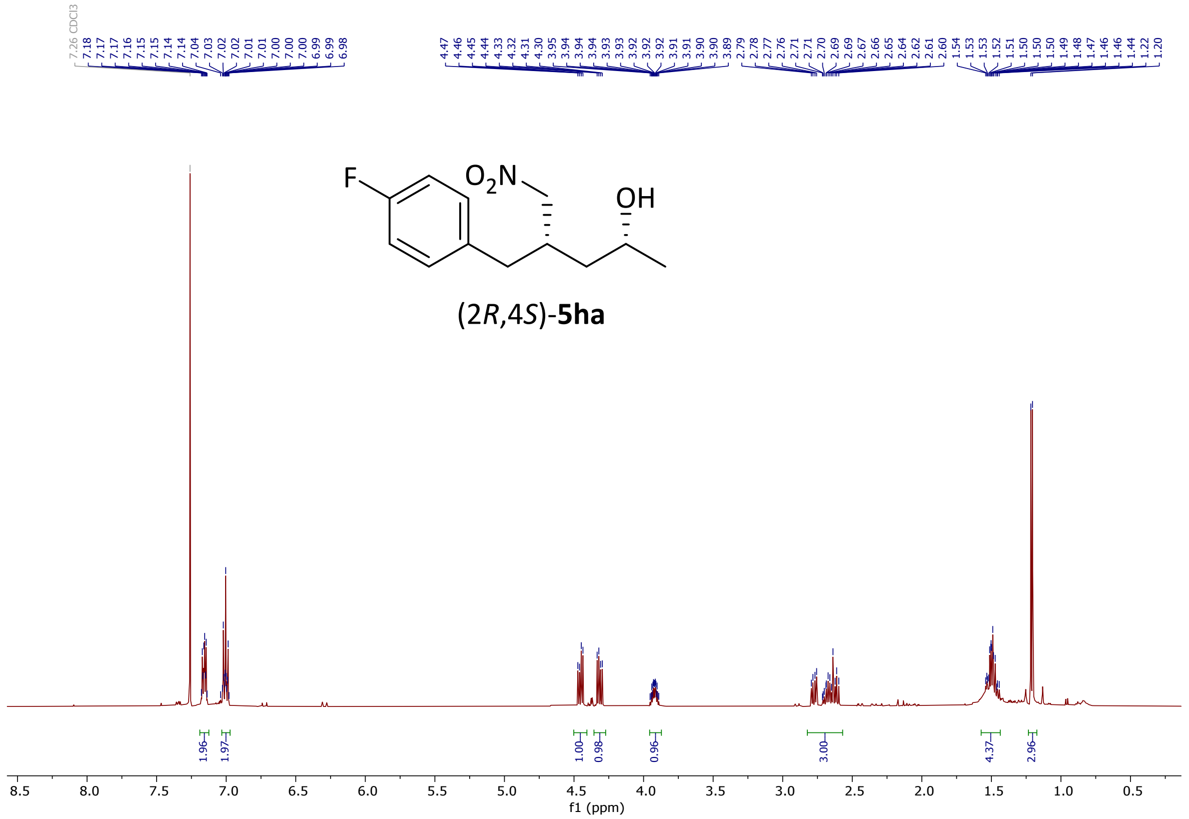

Figure S43. ^1H NMR spectrum of (2*R*,4*S*)-4-(4-fluorobenzyl)-5-nitropentan-2-ol (**5ha**). (500 Hz, $CDCl_3$).

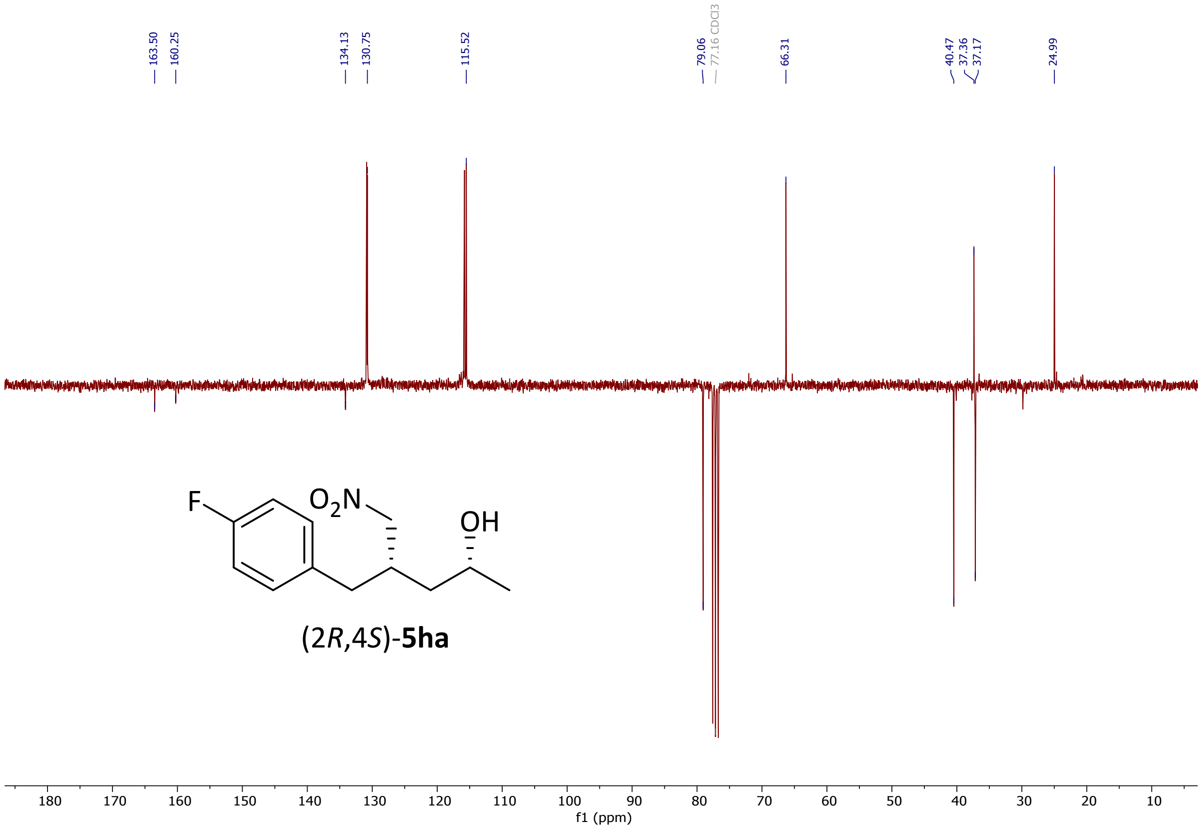

Figure S44. $^{13}C\{^{1}H\}$-APT NMR spectrum of (2*R*,4*S*)-4-(4-fluorobenzyl)-5-nitropentan-2-ol (**5ha**). (75 Hz, $CDCl_3$).

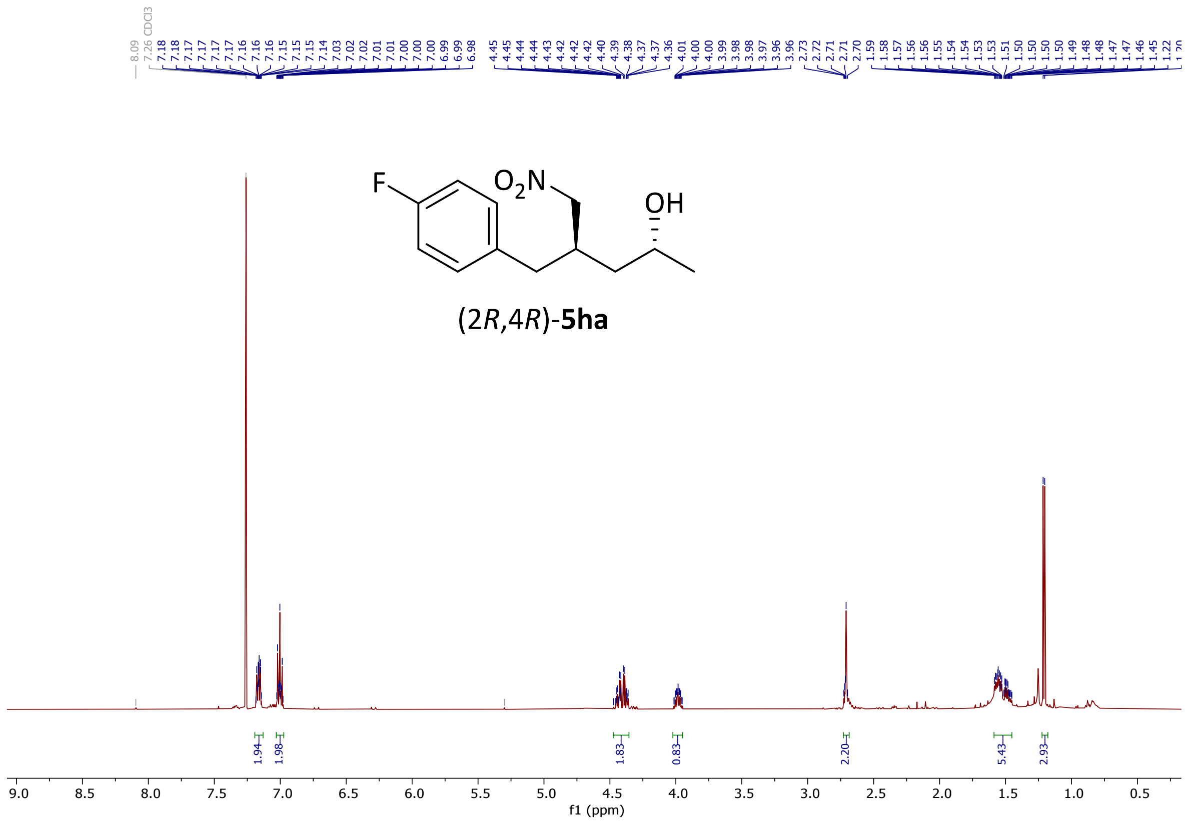

Figure S45. ^{1}H NMR spectrum of (2*R*,4*R*)-4-(4-fluorobenzyl)-5-nitropentan-2-ol (**5ha**). (500 Hz, $CDCl_3$).

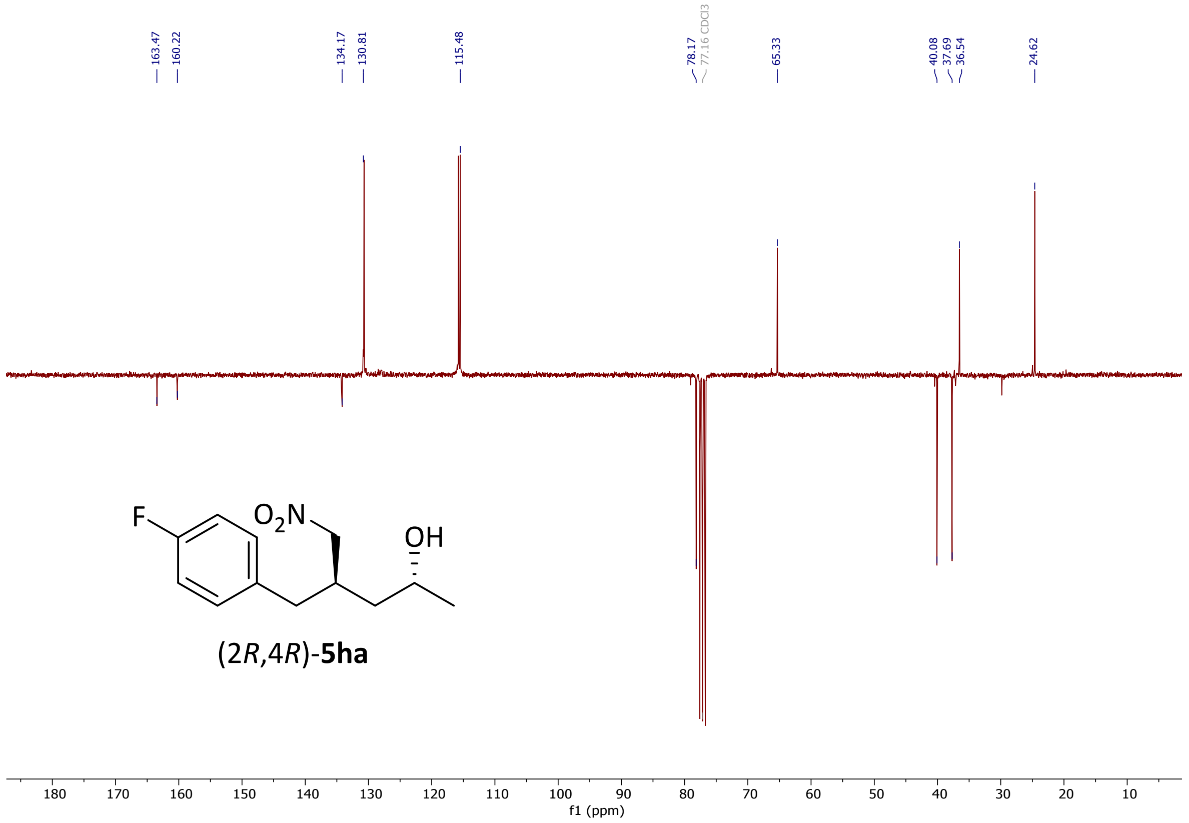

Figure S46. $^{13}C\{^{1}H\}$-APT NMR spectrum of (2*R*,4*R*)-4-(4-fluorobenzyl)-5-nitropentan-2-ol (**5ha**). (75 Hz, $CDCl_3$).

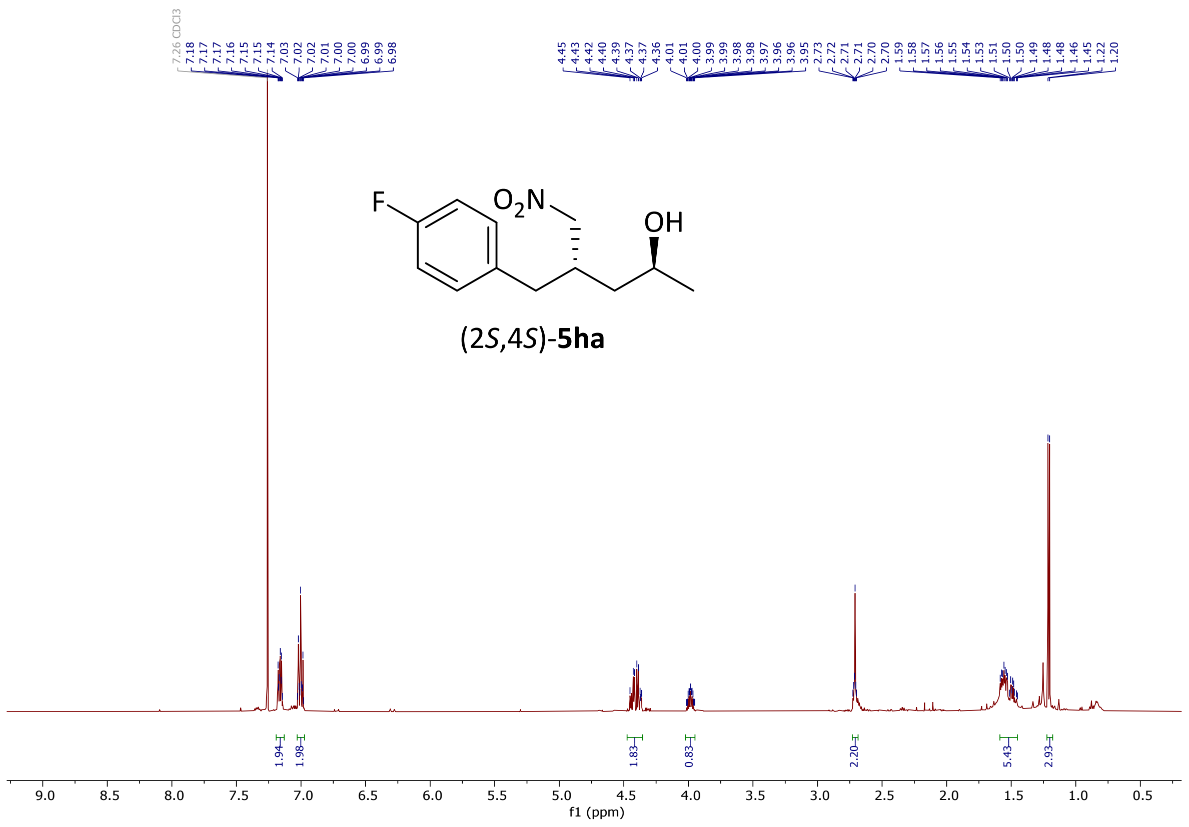

Figure S47. ^{1}H NMR spectrum of (2*S*,4*S*)-4-(4-fluorobenzyl)-5-nitropentan-2-ol (**5ha**). (500 Hz, $CDCl_3$).

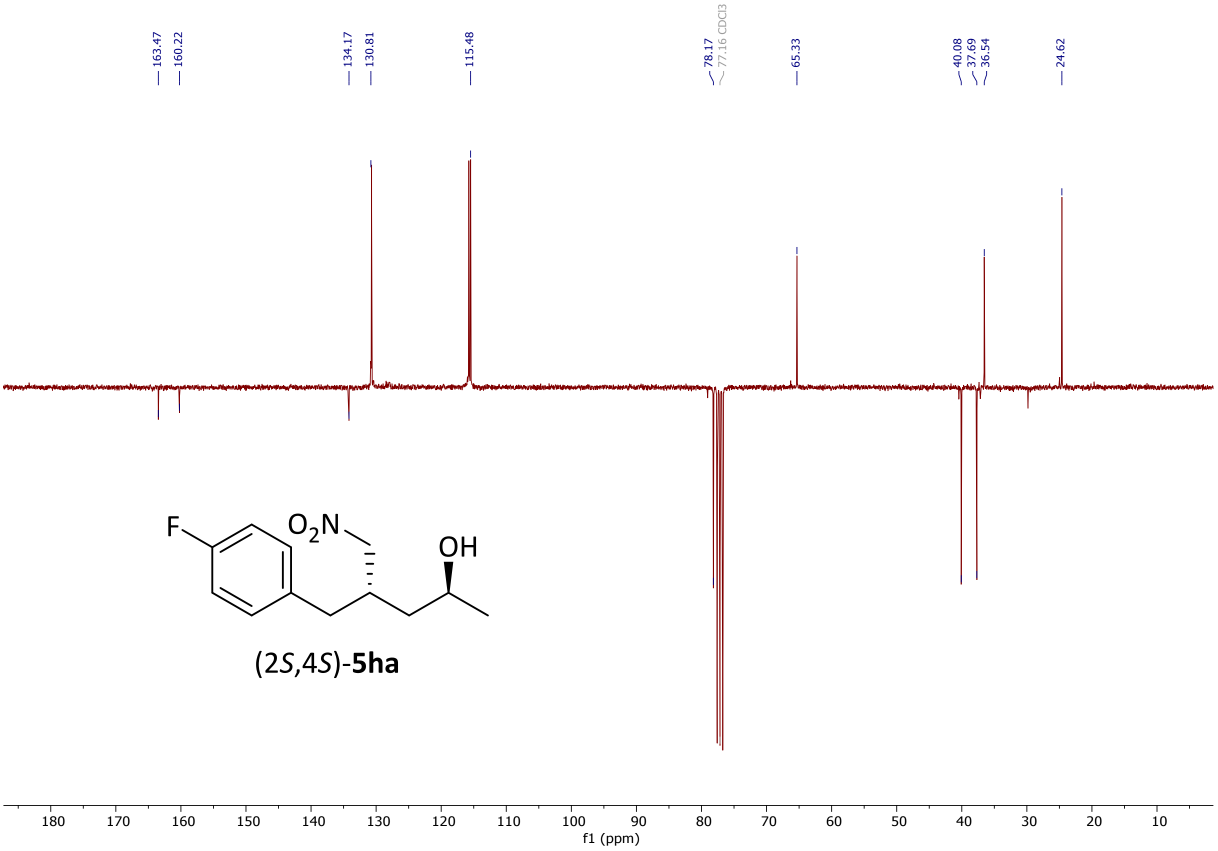

Figure S48. ^{13}C{^{1}H}-APT NMR spectrum of (2*S*,4*S*)-4-(4-fluorobenzyl)-5-nitropentan-2-ol (**5ha**). (75 Hz, $CDCl_3$).

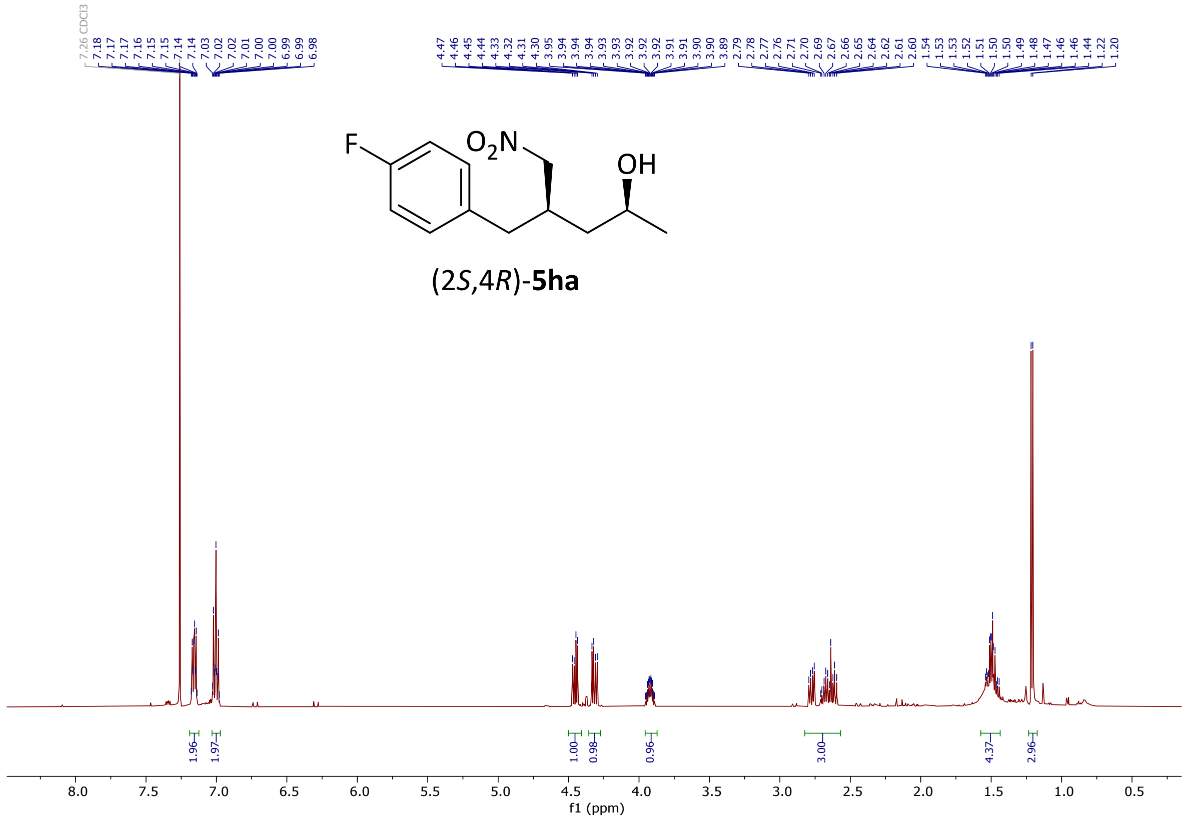

Figure S49. ^{1}H NMR spectrum of (2*S*,4*R*)-4-(4-fluorobenzyl)-5-nitropentan-2-ol (**5ha**). (500 Hz, $CDCl_3$).

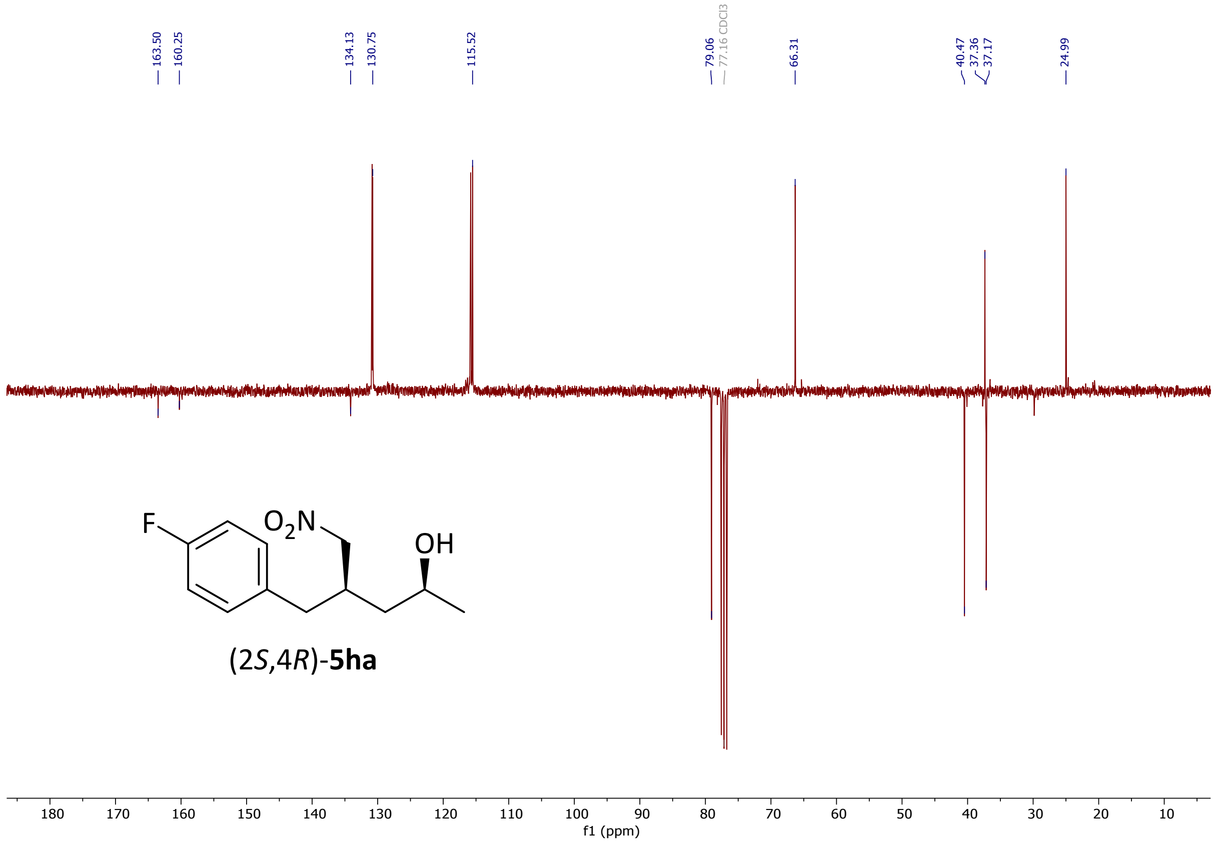

(2*S*,4*R*)-**5ha**

Figure S50. $^{13}C\{^{1}H\}$-APT NMR spectrum of (2*S*,4*R*)-4-(4-fluorobenzyl)-5-nitropentan-2-ol (**5ha**). (75 Hz, $CDCl_3$).

14. Determination of enantiomeric and diastereomeric ratios by NMR

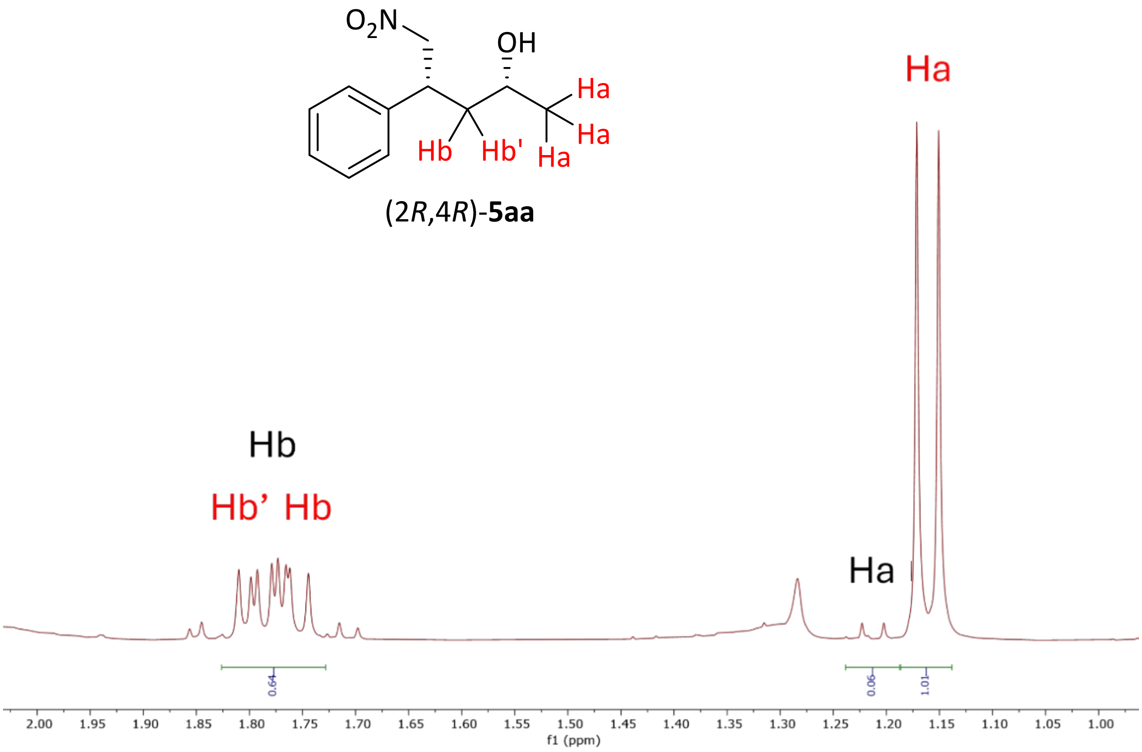

Figure S51. Detail of relevant areas in the ^{1}H NMR of (2*R*,4*R*)-**5aa** for the determination of diastereomeric and enantiomeric ratios.

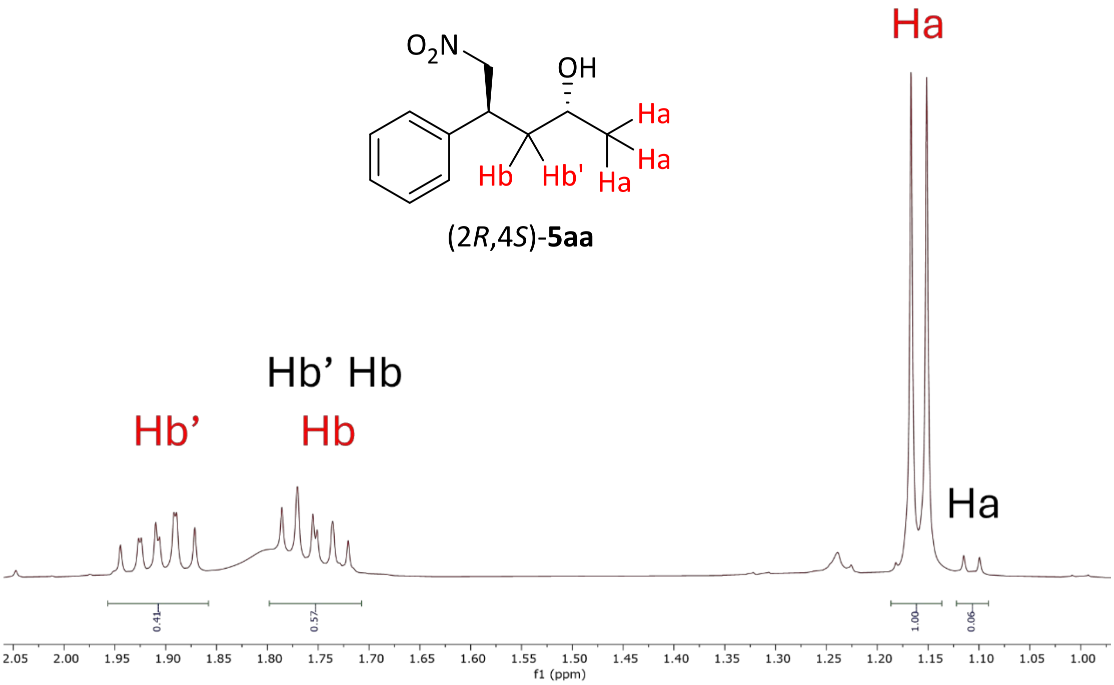

Figure S52. Detail of relevant areas in the ^{1}H NMR of (2*R*,4*S*)-**5aa** for the determination of diastereomeric and enantiomeric ratios.

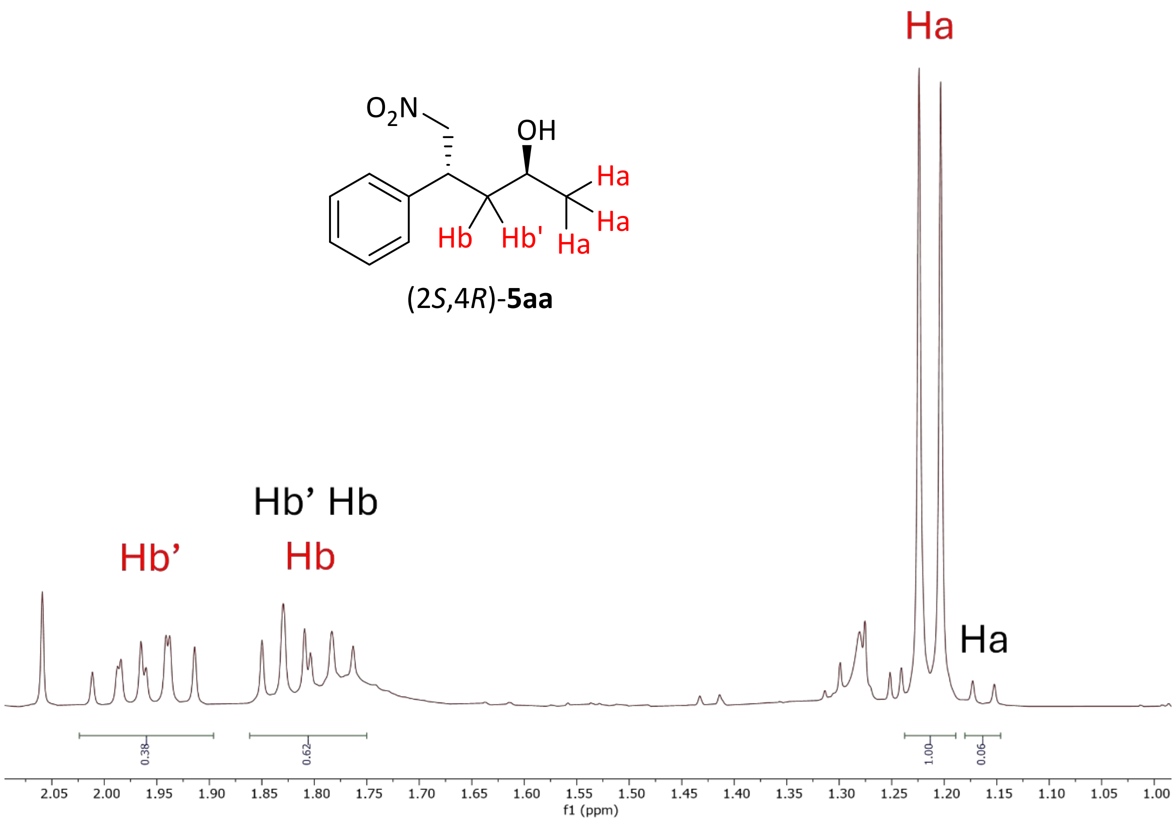

Figure S53. Detail of relevant areas in the ^{1}H NMR of (2*S*,4*R*)-**5aa** for the determination of diastereomeric and enantiomeric ratios.

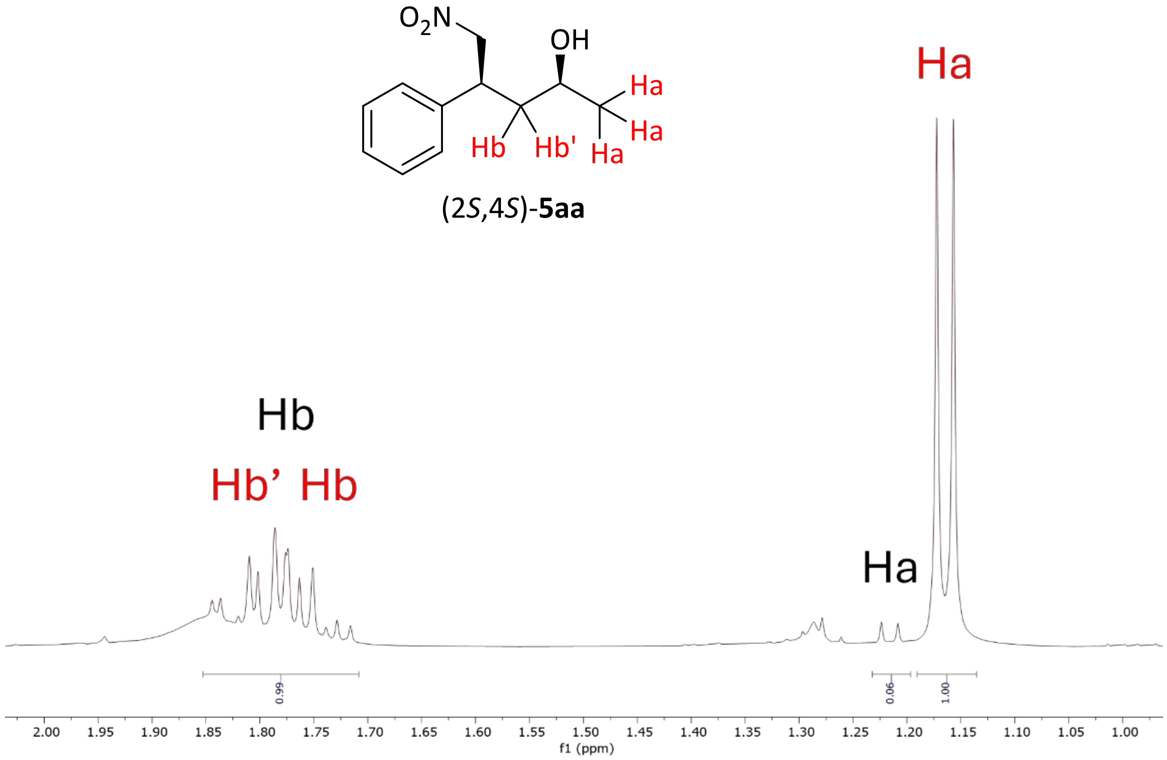

Figure S54. Detail of relevant areas in the ^{1}H NMR of (2*S*,4*S*)-**5aa** for the determination of diastereomeric and enantiomeric ratios.

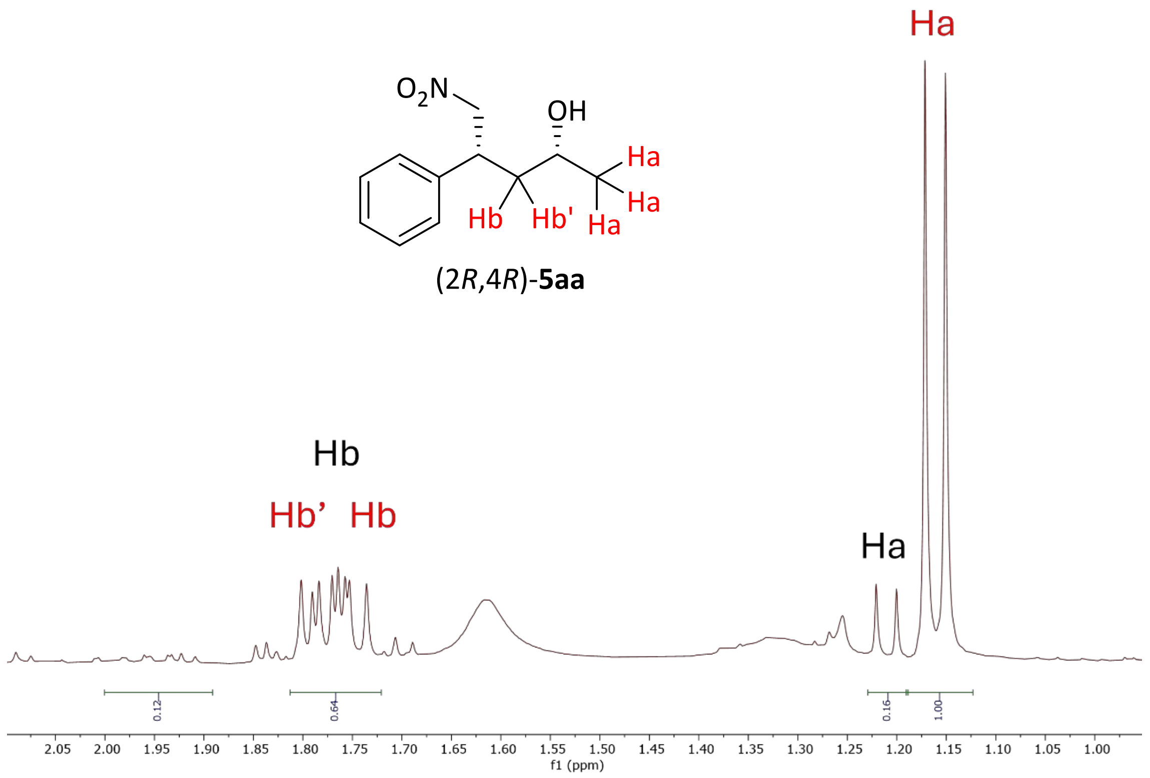

Figure S55. Detail of relevant areas in the ^{1}H NMR of (2*R*,4*R*)-**5aa** for the determination of diastereomeric and enantiomeric ratios. Product obtained from a scale-up performed at a 1 mmol scale.

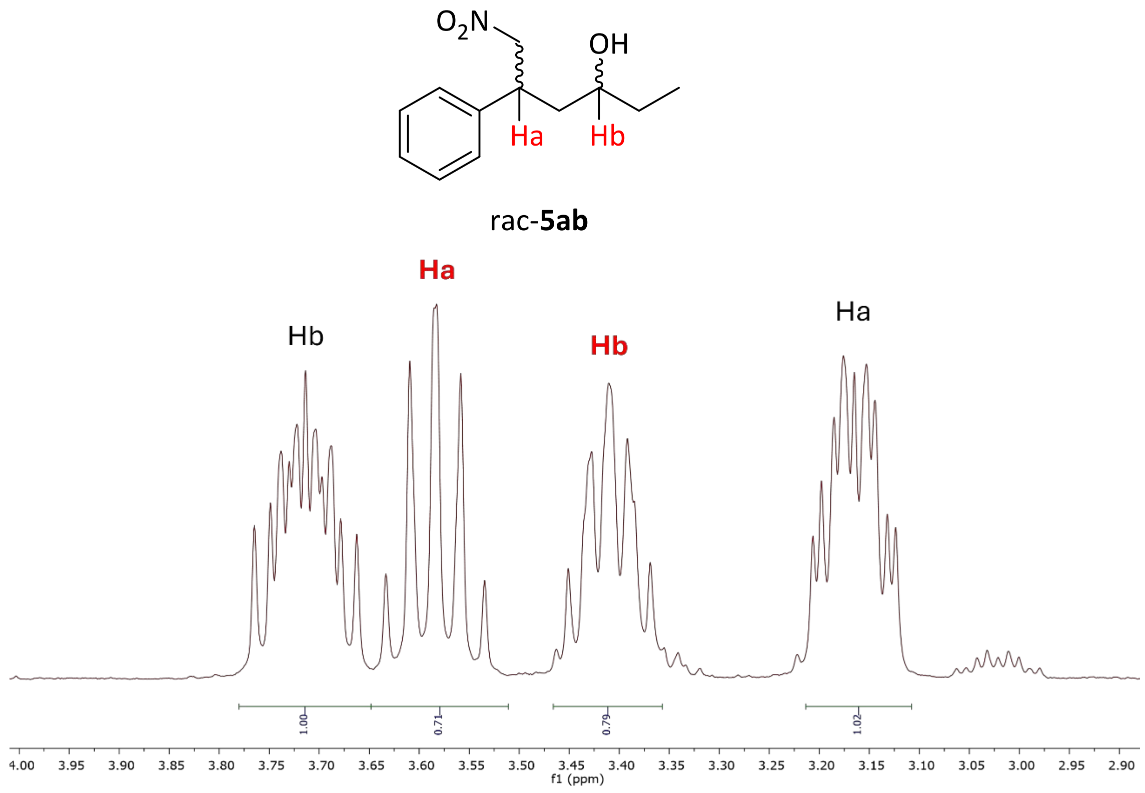

Figure S56. Detail of relevant areas in the ^{1}H NMR of racemic **5ab** for the determination of diastereomeric and enantiomeric ratios.

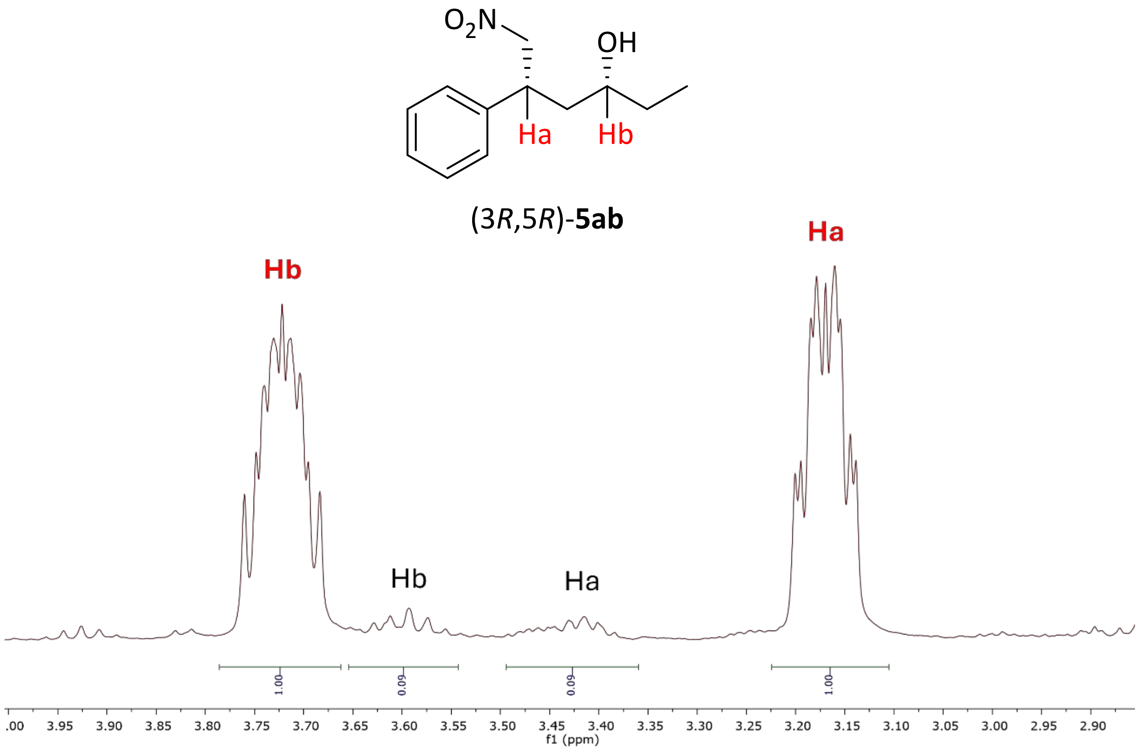

Figure S57. Detail of relevant areas in the ^{1}H NMR of (3*R*,5*R*)-**5ab** for the determination of diastereomeric and enantiomeric ratios.

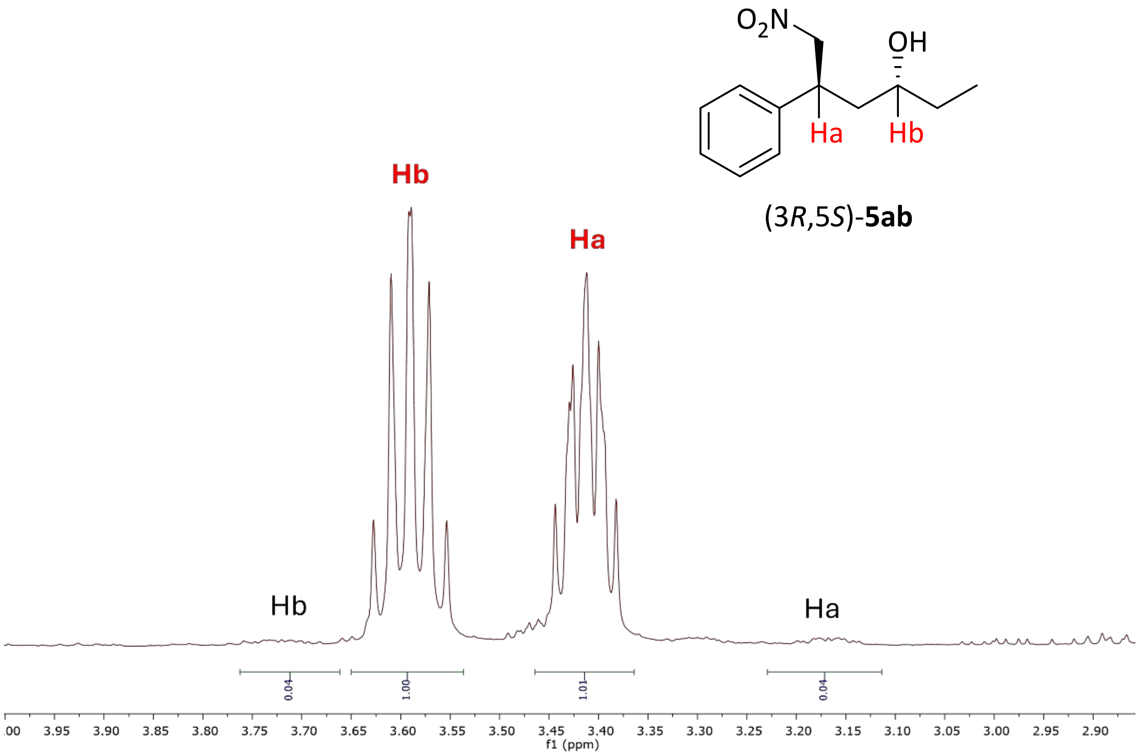

Figure S58. Detail of relevant areas in the ^{1}H NMR of (3*R*,5*S*)-**5ab** for the determination of diastereomeric and enantiomeric ratios.

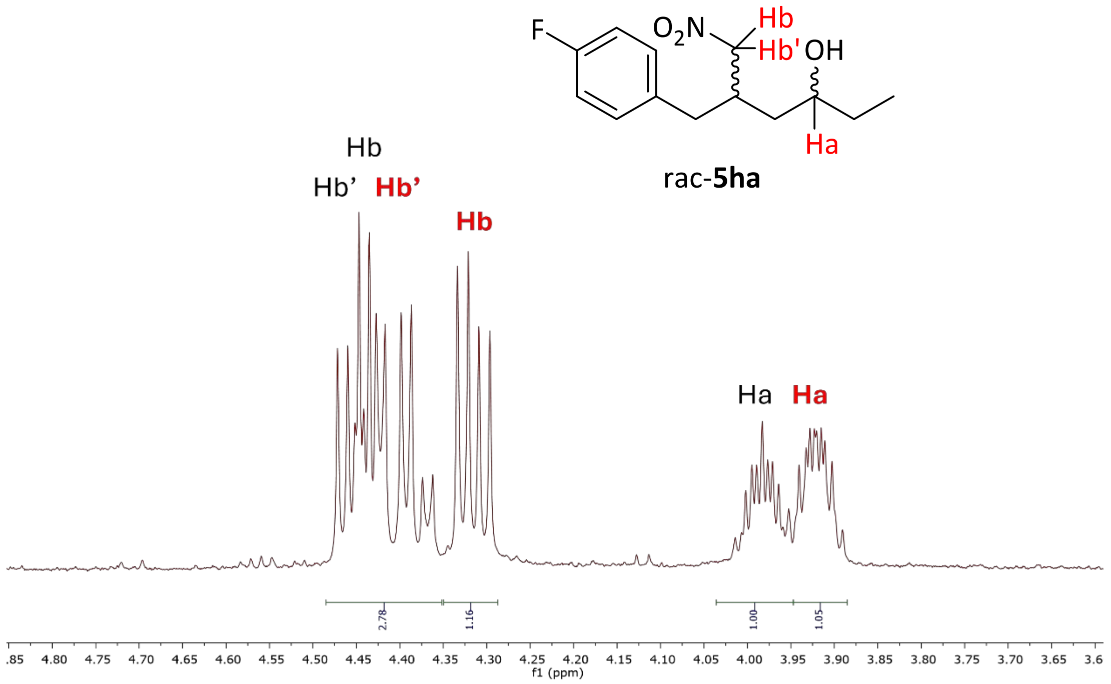

Figure S59. Detail of relevant areas in the ^{1}H NMR of racemic **5ha** for the determination of diastereomeric and enantiomeric ratios.

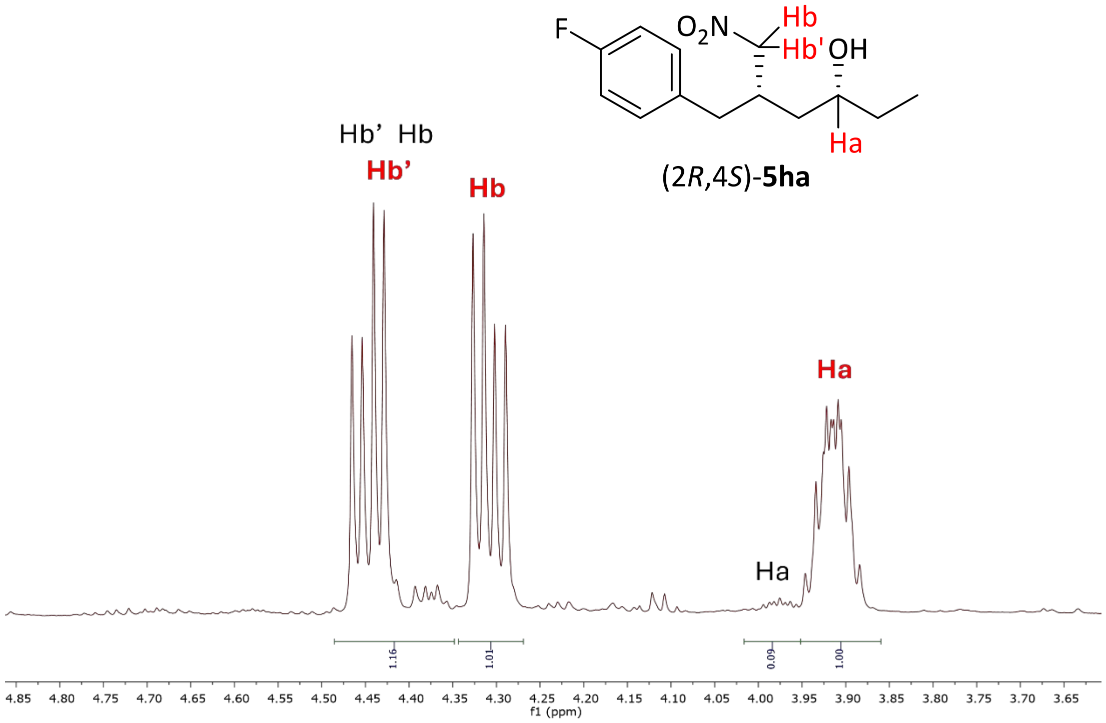

Figure S60. Detail of relevant areas in the ^{1}H NMR of (2*R*,4*S*)-**5ha** for the determination of diastereomeric and enantiomeric ratios.

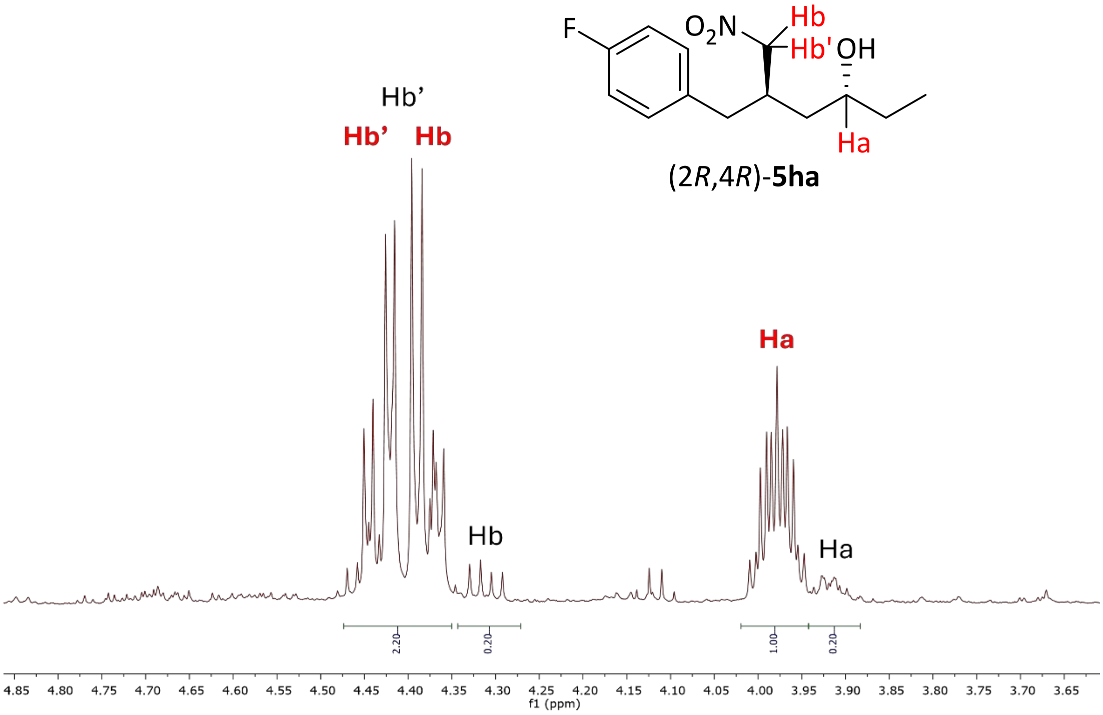

Figure S61. Detail of relevant areas in the ^{1}H NMR of (2*R*,4*R*)-**5ha** for the determination of diastereomeric and enantiomeric ratios.

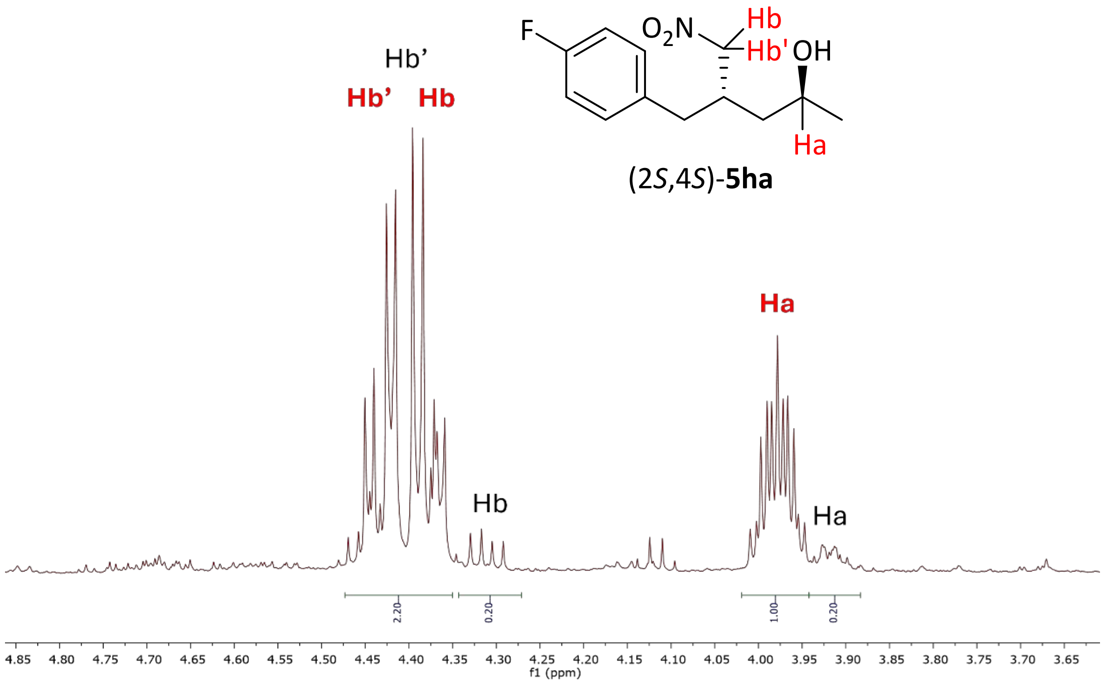

Figure S62. Detail of relevant areas in the ^{1}H NMR of (2*S*,4*S*)-**5ha** for the determination of diastereomeric and enantiomeric ratios.

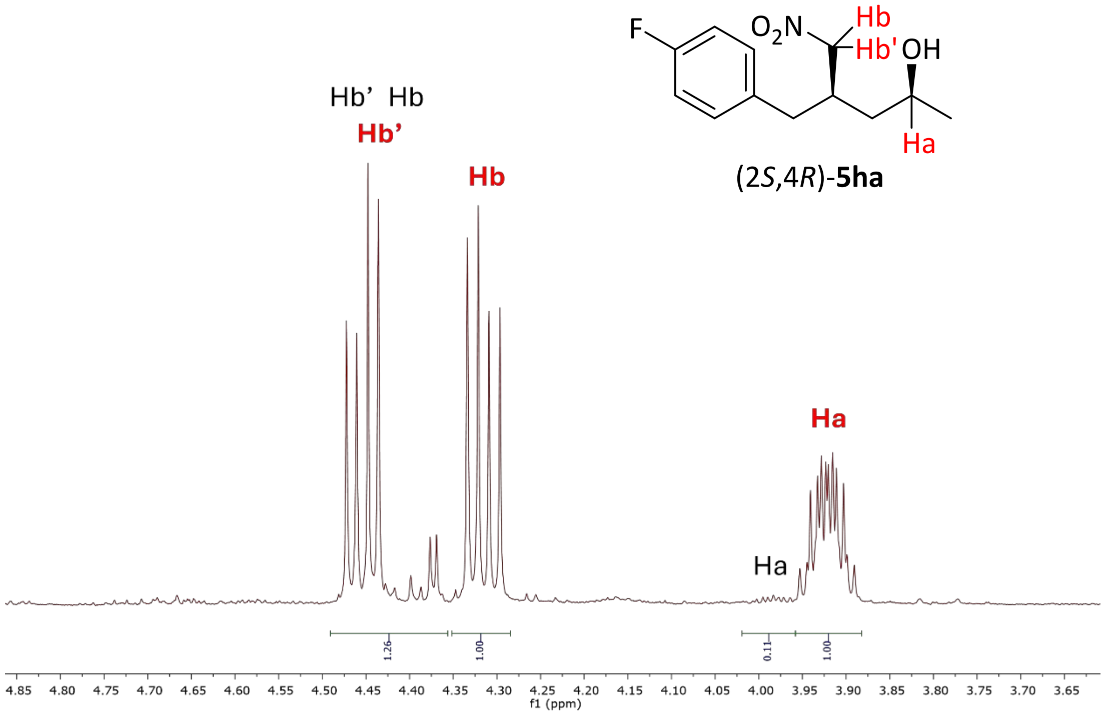

Figure S63. Detail of relevant areas in the ^{1}H NMR of (2*S*,4*R*)-**5ha** for the determination of diastereomeric and enantiomeric ratios.

15. Calibration curves for the determination of conversion in metathesis reactions by GC

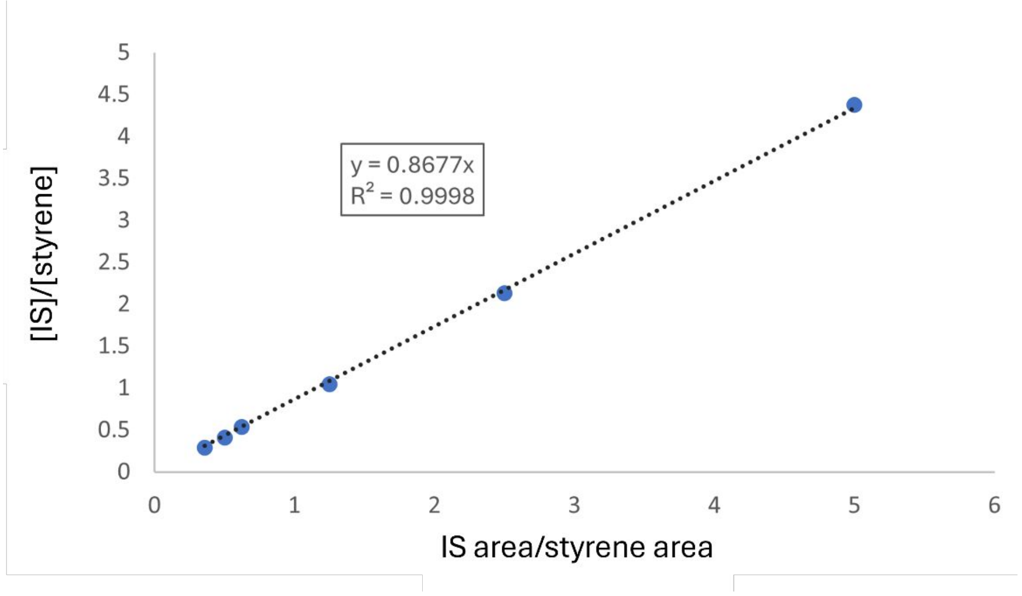

Figure S64. Calibration curve of styrene **1a** for determining conversion in olefin metathesis reactions by GC.

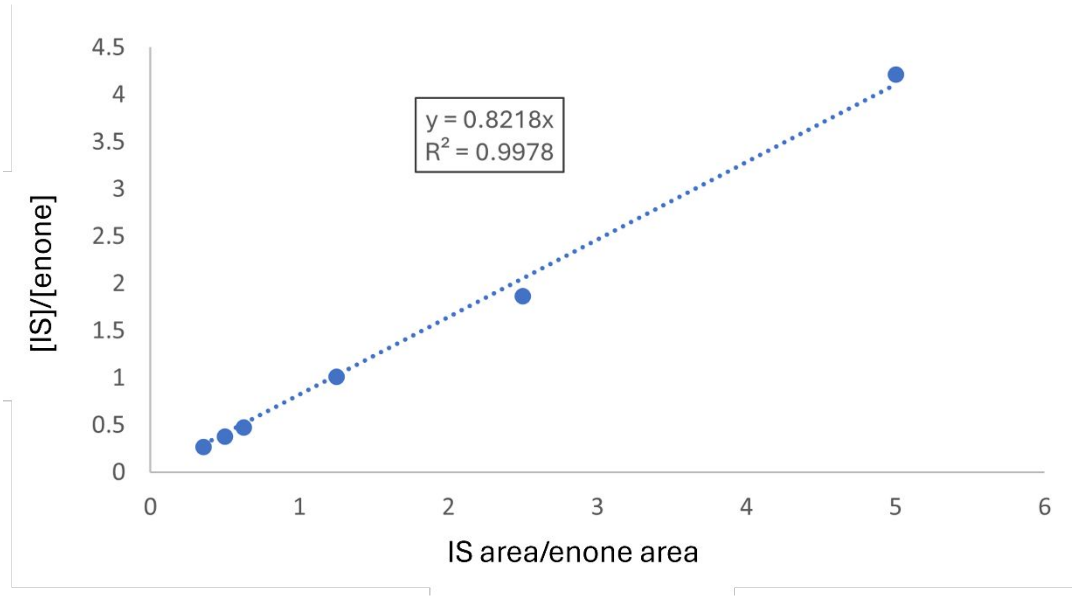

Figure S65. Calibration curve of enone **3aa** for determining conversion in olefin metathesis reactions by GC.
